# Molecular Phylogeny of the *Lactuca* Alliance (Cichorieae Subtribe Lactucinae, Asteraceae) with Focus on Their Chinese Centre of Diversity Detects Potential Events of Reticulation and Chloroplast Capture

**DOI:** 10.1371/journal.pone.0082692

**Published:** 2013-12-20

**Authors:** Ze-Huan Wang, Hua Peng, Norbert Kilian

**Affiliations:** 1 Key Laboratory for Plant Diversity and Biogeography of East Asia, Kunming Institute of Botany, Chinese Academy of Sciences, Kunming, Yunnan, People’s Republic of China; 2 University of Chinese Academy of Sciences, Beijing, People’s Republic of China; 3 Botanic Garden and Botanical Museum Berlin-Dahlem, Freie Universität Berlin, Berlin, Germany; Leibniz-Institute of Plant Genetics and Crop Plant Research (IPK), Germany

## Abstract

The first comprehensive molecular phylogenetic reconstruction of the Cichorieae subtribe Lactucinae is provided. Sequences for two datasets, one of the nuclear rDNA ITS region, the other of five concatenated non-coding chloroplast DNA markers including the *petD* region and the *psbA-trnH, 5′trnL^(UAA)^-trnF*, *rpl32-trnL^(UAG)^* and *trnQ^(UUG)^-5′rps16* spacers, were, with few exceptions, newly generated for 130 samples of 78 species. The sampling spans the entire subtribe Lactucinae while focusing on its Chinese centre of diversity; more than 3/4 of the Chinese Lactucinae species are represented. The nuclear and plastid phylogenies inferred from the two independent datasets show various hard topological incongruences. They concern the internal topology of major lineages, in one case the placement of taxa in major lineages, the relationships between major lineages and even the circumscription of the subtribe, indicating potential events of ancient as well as of more recent reticulation and chloroplast capture in the evolution of the subtribe. The core of the subtribe is clearly monophyletic, consisting of the six lineages, *Cicerbita*, *Cicerbita* II, *Lactuca*, *Melanoseris*, *Notoseris* and *Paraprenanthes*. The *Faberia* lineage and the monospecific *Prenanthes purpurea* lineage are part of a monophyletic subtribe Lactucinae only in the nuclear or plastid phylogeny, respectively. Morphological and karyological support for their placement is considered. In the light of the molecular phylogenetic reconstruction and of additional morphological data, the conflicting taxonomies of the Chinese *Lactuca* alliance are discussed and it is concluded that the major lineages revealed are best treated at generic rank. An improved species level taxonomy of the Chinese Lactucinae is outlined; new synonymies and some new combinations are provided.

## Introduction

### The *Lactuca* Alliance

Lettuce (*Lactuca sativa* L.) is the economically most important crop of the tribe Cichorieae, and *Lactuca* is one of its widest known genera. With almost all members of that tribe, *Lactuca* shares the combined presence of latex and homogamous capitula with usually ligulate 5-toothed flowers. *Lactuca* is also the namegiving member of one of the larger groups of the tribe, which is treated today as the subtribe Lactucinae [1]. In its revised circumscription the Lactucinae comprise about 230 species, distributed in Europe, Africa, Asia and North America [1], with a preference of montane habitats. Many of them are mesic tall forbs, many others are perennial herbs of other kinds, among them the only scandent herbs present in the Cichorieae, or rosette herbs and acaulescent herbs, and more rarely they are xeric subshrubs and annual herbs. This subtribe constitutes the youngest branch in the larger of the two core groups of the Cichorieae, its divergence is estimated to have taken place c. 15–4 Ma ago during the Middle Miocene to Early Pliocene [2]–[3].

The taxonomy of no other alliance of the tribe has faced so many controversies over the last 200 years than that of *Lactuca* and its presumed allies. This pertains to the circumscription and systematic position of the *Lactuca* alliance within the tribe as well as, and even much more so, to the generic classification of its members. In the 19th century, the *Lactuca* alliance, although sometimes recognised as a separate subtribe [4], was mostly included in the subtribe Crepidinae, as was done also by Hoffmann [5], whose treatment became influential and the basis for most of the 20th century flora treatments. Also in the first two important 20th century classifications of the Cichorieae, by Stebbins [6] and Jeffrey [7], the Lactuca alliance was treated as a subgroup of the Crepidinae or of a corresponding entity: the first author treated it as the *Prenanthes-Lactuca* line of subtribe Crepidinae, the second as the *Prenanthes* series of the *Crepis* group.

Only towards the end of the 20th century, the *Lactuca* alliance was recognised as a separate subtribe Lactucinae by Bremer [8], after his morphological phylogenetic analysis of the tribe had revealed the Crepidinae to be polyphyletic. Bremer therefore divided the Crepidinae into the three subtribes Lactucinae, Crepidinae s.str. and Sonchinae, which were largely maintained by Lack [9]. Based on a nrITS phylogeny of the Cichorieae, which remarkably well agrees with the results inferred from chloroplast DNA restriction site variation [10], Kilian & al. [1] maintained these three subtribes of Bremer among the 11 subtribes they recognised in the tribe, but narrowed down the circumscription of subtribe Lactucinae compared to Bremer [8] and Lack [9]. Kilian & al. [1] excluded from subtribe Lactucinae the genera *Prenanthes* s.l., which was characterised as a dust-bin of various unrelated elements by Kilian & Gemeinholzer [11], and *Faberia*, as well as *Nabalus* and *Syncalathium*, the last two having been recognised as members of subtribe Crepidinae s.str. The exclusion from subtribe Lactucinae of all elements of the polyphyletic genus *Nabalus*, which is represented in China, depending on the species concept, by four species (under *Prenanthes*) [12] or only one (plus one additionally included species) [13] and of all but one species of *Syncalathium* has been corroborated recently by Zhang & al. [2], [14].

### Subtribe Lactucinae in its Chinese Centre of Diversity

The subtribe has two centres of current diversity, one in the Mediterranean-SW Asian region, the other in China and the adjacent Himalayan region. The diversity of the subtribe in its Mediterranean-SW Asian centre came into the focus of systematic research around the middle of the 19th century and led to the description of many new species and two new genera, *Cephalorrhynchus* Boiss. in 1844 and *Steptorhamphus* Bunge in 1852. A first comprehensive treatment of the members of the Lactucinae in this centre was provided by Boissier [15] (p. 795ff, as parts of subtribe “Crepideae”). Noteworthy among the more recent publications is in particular the taxonomic revision of the *Lactuca* alliance in the Iranian Highlands and neighbouring regions by Tuisl [16].

In contrast, the actual extent of the subtribe’s diversity in its Sino-Himalayan centre remained unveiled much longer, apart from the Himalayan portion, which was covered rather early by Clarke [17] and Hooker [18], with the most recent updates by Mamgain & Rao [19] and Grierson & Long [20]. Although many species of the subtribe in the large territory of China were discovered and described already in the late 19th and early 20th century, and some of them were included in the revision of *Cicerbita* sensu lato by Beauverd [21], the subtribe in China became subject of comprehensive studies only towards the end of the 20th century. Pioneer works were done almost exclusively by Shih [12], [22]–[25], who described the new genera *Chaetoseris* C. Shih, *Faberiopsis* C. Shih & Y. L. Chen, *Notoseris* C. Shih, *Paraprenanthes* C. C. Chang ex C. Shih, *Pterocypsela* C. Shih, *Stenoseris* C. Shih to accommodate the diversity of the subtribe encountered. Shih subsequently also provided the first comprehensive floristic treatment of the entire tribe Cichorieae in China [12]. The subtribal classification applied by Shih largely conforms to that of Stebbins [6], but with corrected subtribal nomenclature. Shih’s [12] Lactucinae (corresponding to the Crepidinae s.l. of Stebbins 1953) span the four subtribes Crepidinae s.str., Hieraciinae, Lactucinae s.str. and Hyoseridinae as recognised in the current classification by Kilian & al. [1].

Recently, a reappraisal of the systematics of the *Lactuca* alliance in China, supported by our then still initial nrITS phylogeny of the subtribe including representatives of most Chinese groups, was provided in the frame of the English “Flora of China” [13]. The most striking difference to the treatment by Shih [12] concerns the generic classification: whereas the species of the Lactucinae sensu Kilian & al. [1] were classified by Shih [12] in altogether 12 genera (*Cephalorrhynchus*, *Chaetoseris*, *Cicerbita*, *Lactuca*, *Lagedium*, *Mulgedium*, *Notoseris*, *Paraprenanthes*, *Prenanthes*, *Pterocypsela*, *Scariola*, *Stenoseris*), they were placed in only five genera (*Cicerbita, Lactuca, Melanoseris, Notoseris* and *Paraprenanthes*) by Shih & Kilian [13]. Such different generic classification of the *Lactuca* alliance is symptomatic for the entire history of the systematics of this alliance. No stability in generic classification has been reached over more than 200 years, because morphological features fail to provide unanimous support for any classification proposed.

Hitherto many Chinese Lactucinae species were only known from herbarium material but never studied in the wild. The first author of the present paper, in contrast, has succeeded to study, collect and sample most Chinese species of the subtribe in the wild, in addition to herbarium studies. Consequently, our initial, sparse molecular sampling of Chinese taxa for nrITS available during the preparation of the “Flora of China” account, now has grown to include the vast majority of the species of the *Lactuca* alliance in China and the nuclear dataset has been complemented by a chloroplast dataset.

The aims of the present paper are (1) to provide the first molecular phylogeny of the Lactucinae which, although focusing on the Chinese centre of diversity, spans the entire subtribe; (2) to detect potential events of reticulation in the evolution of the subtribe by comparing corresponding nuclear and plastid datasets; (3) to test the robustness of the different taxonomies of the Chinese *Lactuca* alliance in the light of evolution as inferred from the nuclear and plastid trees; (4) to improve the taxonomy of the Chinese Lactucinae based on the molecular phylogenetic reconstruction and morphological studies of living plants and herbarium material including types.

## Materials and Methods

### Plant Material

The authors have studied herbarium material from the herbaria A, B, CAS, CDBI, E, G, GH, K, KUN, MO, NY, PE and SZ (herbarium codes following Thiers [26]) as well as from the personal herbaria of Ralf Hand (Berlin, Germany), Georg & Sabine Miehe (Marburg, Germany) and Michael Ristow (Potsdam, Germany), of almost all species of the subtribe known from China and adjacent areas, including the types, and the first author extensively studied and collected most Chinese species also in the wild (collection deposited at KUN with some duplicates at B). Besides the permissions for the nature reserves in the Chinese provinces of Chongqing, Sichuan, Xizang and Yunnan by the corresponding Provincial Forestry Departments, no specific permissions were required for material collection; the locations are not privately-owned and none of the species collected in the field are endangered or protected.

### Sampling Strategy

Our sampling for the molecular analyses aimed at a dense representation of the subtribe Lactucinae in China. This has been achieved largely so, with the only exception of a few species in North China with Central Asian relation, of which no material could be gathered for this study but which will be included in our global phylogeny of the subtribe (unpublished data). Sequences of one nuclear and five plastid markers were obtained for a total of 130 samples of 78 species. Except for 9, all of the 767 individual marker sequences involved were newly generated for this study. Among the 126 ingroup samples, there are 119 samples of Lactucinae species of China, representing 66 species and 76.7% of the total 86 species recognised by Shih [12], or 55 species and 77.5% of the total species recognised by Shih & Kilian [13], respectively. The corresponding information on the material, including the vouchers preserved, is listed in Appendix S1. Our taxon sampling includes the species providing the types of all generic names established in the subtribe that are relevant to the Lactucinae in China as based on our global phylogeny of the subtribe (unpublished data); these are the types of *Cephalorrhynchus* Boiss. (*C. glandulosus* Boiss. ≡ *C. hispidus* (DC.) Boiss.), *Chaetoseris* C. Shih (*C. lyriformis* C. Shih), *Cicerbita* Wallr. (*C. alpina* (L.) Wallr.), *Faberia* Hemsl. (*F. sinensis* Hemsl.), *Lactucella* Nazarova (*L. undulata* (Ledeb.) Nazarova), *Lagedium* Soják (*L. sibiricum* (L.) Soják), *Melanoseris* Decne. (*M. lessertiana* (DC.) Decne.), *Mulgedium Cass.* (*M. runcinatum* Cass. = *M. tataricum* (L.) DC.), *Notoseris* C. Shih (*N. psilolepis* C. Shih), *Paraprenanthes* C. C. Chang ex C. Shih (*P. sororia* (Miq.) C. Shih), *Prenanthes* L. (*P. purpurea* L.), *Parasyncalathium* J. W. Zhang & al. (*P. souliei* (Franch.) J. W. Zhang & al.), *Pterocypsela* C. Shih (*P. indica* (L.) C. Shih), *Stenoseris* C. Shih (*S. graciliflora* (Wall. ex DC.) C. Shih), *Scariola* F. W. Schmidt (*S. viminea* (L.) F. W. Schmidt) and *Steptorhamphus* Bunge (*S. tuberosus* (Jacq.) Grossh.) [27]. For many species several individuals were sampled to cover the morphological variation observed, and, wherever possible, samples were gathered from, or as close as possible to, the type locality.

As outgroup, we selected four taxa of the subtribes Crepidinae (*Crepis* and *Soroseris*), Hyoseridinae (*Launaea*) and Hypochaeridinae (*Leontodon*), which represent the decreasingly related other subtribes of the same core group of the Cichorieae according to the molecular analyses by Kilian & al. [1] and Tremetsberger & al. [3]. *Launaea sarmentosa* (subtribe Hyoseridinae) was used to root the trees.

### Dna Isolation, Amplification And Sequencing

Genomic DNA was extracted from c. 20 mg of silica-dried leaf tissue or recently collected specimens, either using a modified CTAB methods [28], or the DNeasy kit (Qiagen GmbH, Germany) or Plant Kit Rev. 03 (Macherey-Nagel GmbH & Co. KG, Germany), following the manufacturer’s protocols. The DNA amplifications were performed using T1 or T3 Thermocyclers (Biometra, Göttingen, Germany). The amplification reactions with a total volume of 25 µl were of one of the following two compositions: (A) 2 µl DNA template with a concentration of c.15 ng, 1 µl of each primer (5 pm/µl), 1.5 µl Mg^2+^ (13.9 pm/µl), 2.5 µl dNTP mix (2 pm/µl), 2.5 µl×10 Taq reaction Buffer (Chenlü, Kunming, China), 1 µl BSA (bovine serum albumin, 10 ng/µl), 0.3 µl Taq DNA polymerase (2.5 U/µl) (Chenlü, Kunming, China), H_2_O; (B) 1 µl DNA template of 20 ng/µl, 1 µl of each primer (10 pm/µl), 1.5 µl MgCl_2_ (1.25 mM), 2.5 µl dNTP mix (1.25 pm/µl), 2.5 µl 10x peqLab Taq. Buffer S, 2.5 µl Betain (1.25 mM) [or: 1.5 µl BSA (1.25 mM)], 0.15 µl peqLab HOT Taq. Polymerase (5 units/µl), H_2_O.

One nuclear and five non-coding chloroplast regions were used as markers. The nuclear ribosomal Internal Transcribed Spacer (nrITS) region (ITS1, 5.8S rDNA, ITS2) was amplified using either the primer combinations ITS4/ITS5 [29] or ITSA/ITSB [30]. Amplification conditions were as follows: an initial denaturation step at 95°C for 3 min, followed by 29 cycles of denaturation at 95°C for 30 s, annealing at 53°C for 30 s, and extension at 72°C for 45 s, then a final extension step at 72°C for 8 min.

The chloroplast markers were amplified using the following primers: (1) the *petD* intron and *petB-petD* spacer were co-amplified with the universal primers PIpetB1411F/PIpetD738R [31]; (2) the *psbA-trnH* spacer with the universal primers psbAF/trnHR [32]; (3) the *5′trnL(UAA)-trnF* spacer with the universal primers trnC/trnF [33]; (4) the *rpl32-trnL(UAG)* spacer with the primers rpl32-F/trnL(UAG) [34] and (5) the *trnQ(UUG)-5′rps16* spacer with the primers trnQ(UUG)/rps16x1 [34]. The PCR amplification conditions were identical for all five chloroplast markers: an initial denaturation step at 80°C for 5 min, followed by 29 cycles consisting of denaturation at 94°C for 45 s, annealing at 52°C for 45 s, extension at 65°C for 50 s, and a final extension step at 65°C for 7 min.

Amplification products and negative controls were visualised in a 1 or 1.2% NEEO agarose electrophorese gel and purified for sequencing using the QIAquick PCR purification Kit (BioTeke Corporation, Beijing, China or Qiagen GmbH, Germany) following the manufacturer’s instructions. The concentrations of the purified PCR products were measured with a NanoDrop spectrophotometer (ND-1000, PeqLab, Erlangen, Germany). The purified products were directly sequenced on an ABI 3730XL automated DNA sequencer (Applied Biosystems, Foster City, California, USA) or sequenced via StarSeq (Mainz, Germany) with the same primers as used for amplification.

### Sequence Alignment And Coding Of Length Mutational Events

The boundaries of the nrITS region (ITS1, 5.8S rDNA, ITS2) and the *petD* marker (*petD* intron and *petB-petD* spacer) were defined according to Goertzen & al. [35] and Borsch & al. [36], respectively. The boundaries of the other markers were taken as indicated in the complete chloroplast genome sequence of *Lactuca sativa* (EMBL/Genbank/DDBJ DQ383816) by Timme & al. [37].

The ITS sequences were aligned manually in PhyDE version 0.9971 [38], according to the Cichorieae part of the Asteraceae alignment by Goertzen & al. [35], which was based on their secondary structure analyses. The plastid sequences were first automatically aligned using Muscle [39], then adjusted manually to a motif-based alignment in PhyDE [38] following the criteria outlined by Kelchner [40], Borsch & al. [41] and Löhne & Borsch [31]. Regions of uncertain homology were excluded from the analysis and inversions were re-inverted (as documented in Appendix S2) prior to the phylogenetic reconstruction.

Indels (as documented in Appendix S3) were coded as informative characters according to the Simple Indel Coding (SIC) method [42] as implemented in the program SeqState version 1.40 [43]. SIC performs about as good as the Modified Complex Indel Coding (MCIC) [44] but has the advantage that the SIC matrix can also be easily analysed with Bayesian Inference.

Additive polymorphic sites (APS) in the nrITS sequences, indicating potential introgressive hybridisation, were detected following the criteria outlined by Fuertes Aguilar & Nieto Feliner & al. [45].

### Phylogenetic Reconstruction

Incongruence Length Difference (ILD) test [46] implemented in PAUP* version 4.0b10 [47] as the Partition Homogeneity Test, was performed to assess the congruence between the nuclear and plastid data sets. For this test, which calculates the ILD first for the original partitions and then for a series of randomized partitions of the same size, the following parameters were used: heuristic search of 10 000 replicates, each with 100 random addition searches, maxtrees set to 1 and one tree held each step. As significance threshold for congruence or homogeneity of the partitions a P value of >0.01 is considered as appropriate [48].

Phylogenetic relationships were reconstructed using Maximum Parsimony (MP) and Bayesian Inference (BI). Maximum Parsimony analyses were performed using the Parsimony Ratchet [49] with PRAP [50] in combination with PAUP* version 4.0b10 [47]. Standard ratchet settings were used: 200 ratchet iterations with 25% of the positions randomly upweighted (weight = 2) during each replicate and 10 random addition cycles. The generated command files also including the nexus data matrix were run in PAUP* version 4.0b10 [47] using heuristic search with the following parameters: all characters have equal weight, gaps are treated as ‘missing’, simple addition of sequences, TBR branching swapping, maxtrees setting to 100 and auto-increased by 100, one non-binary starting tree arbitrarily dichotomized before branch swapping, only one tree saved. A majority rule consensus tree was calculated from the most parsimonious trees received. Jackknife (JK) support values for the nodes found by the MP analysis were calculated in PAUP*version4.0b10 applying the optimal jackknife parameters according to Farris & al. [51] and Müller [52: 10 000 jackknife replicates were performed using the TBR branch swapping algorithm with 36.788% of characters deleted and one tree held during each replicate.

Bayesian Inference analyses were performed using MrBayes 3.2 [53]. Optimal nucleotide substitutions models were searched separately for each of the three partitions of the nrITS dataset (i.e. ITS1, 5.8S, ITS2) and each of the five plastid markers with MrModeltest 2.3 [54], following the Akaike Information Criterion (AIC). The optimal model chosen for ITS1 and ITS 2 was GTR+I+G, for 5.8S SYM+I, for the *petD* region GTR+I, and for the other four plastid markers GTR+G. A binary (restriction site) model was implemented for the coded indels. The datasets were partitioned in MrBayes 3.2 into three (nuclear) or five (plastid) DNA markers, respectively, and one partition for the coded indels. All analyses in MrBayes 3.2 were performed with four simultaneous runs of Metropolis-coupled Markov Chains Monte Carlo (MCMCMC), each with four parallel Markov chains. Each chain was performed for 2 million generations and, starting with a random tree, one tree was saved every 100th generation. For other parameters the default settings of the program were left unchanged. A conservative burn-in of 0.2 (i.e. discarding the first 20% of the trees) was applied after graphically checking chain convergence using the program AWTY [55]. The remaining trees were used to generate a majority rule consensus tree.

TreeGraph 2 [56] was used to assess the tree topologies and to visualise the trees with node supports.

## Results

### Molecular Datasets And Phylogenetic Analyses

#### Nuclear Ribosomal Its Region

The ITS region varied from 592 to 644 nt in our 130 (126 ingroup +4 outgroup) samples. Of a total of 667 characters in the aligned data set, 261 were parsimony informative. Simple Indel Coding increased the total number of characters to 734 and the number of parsimony informative characters to 301. With 39.1% (41.0% including coded indels) parsimony informative sites it has the highest phylogenetic performance of all markers used, but has the lowest consistency index and retention index of all individual marker trees (Table 1).

**Table 1:** Sequence and tree statistics of the six individual markers and the concatenated plastid matrix.

Data matrix	Length range total/HS^1^ excluded (nt)	No. total char.^2^/No. total char.^3^ (nt)	No. inform. sites^4^/No. inform. sites^3^ (nt)	No.MPTS^3^	TL^3^	CI^3^	RI^3^	RC^3^
**ITS region**	592–644	667/734	261(39.1%)/301(41.0%)	70	1204	0.485	0.840	0.408
***petD region***	887–922/876–906	928/948	67(7.2%)/80(8.4%)	22	171	0.877	0.972	0.853
***psbA-trnH***	171–421/131–382	464/497	62(13.4%)/79(15.9%)	1474	182	0.824	0.926	0.763
***trnL-F***	786–841/737–792	825/857	56(6.8%)/66(7.7%)	28	178	0.899	0.958	0.861
***trnQ-rps16***	929–998/928–997	1174/1221	111(9.5%)/129(10.6%)	131	339	0.861	0.951	0.819
***rpl32-trnL***	830–939/807–894	1139/1223	154(13.5%)/191(15.6%)	19	456	0.844	0.953	0.804
**combined cpDNA**	3784–4028/3619–3884	4530/4746	450(9.9%)/545(11.5%)	48	1342	0.847	0.950	0.805

1 hotspots (and exons), see Table S1;

2 number of total character;

3 with indel coding;

4 number of informative sites.

The Maximum Parsimony (MP) search resulted in 70 most parsimonious trees (L = 1204, CI = 0.485, RI = 0.840, RC = 0.408, see Table 1). The 50% majority rule MP consensus tree was essentially congruent in topology with the Bayesian Inference (BI) 50% majority rule consensus tree, apart from an incongruence in one subclade of the *Lactuca* lineage, where in the BI tree the *L. sativa*-*L. serriola* clade is sister to the *Scariola* and *Lagedium*-*Mulgedium* clades, while in the MP tree the *Lagedium*-*Mulgedium* clade is sister to the other two. We give here only the BI phylogram (Fig. 1), with the MP Jackknife support (JK) values above and the BI posterior probability (PP) values below the branches.

**Figure 1:**
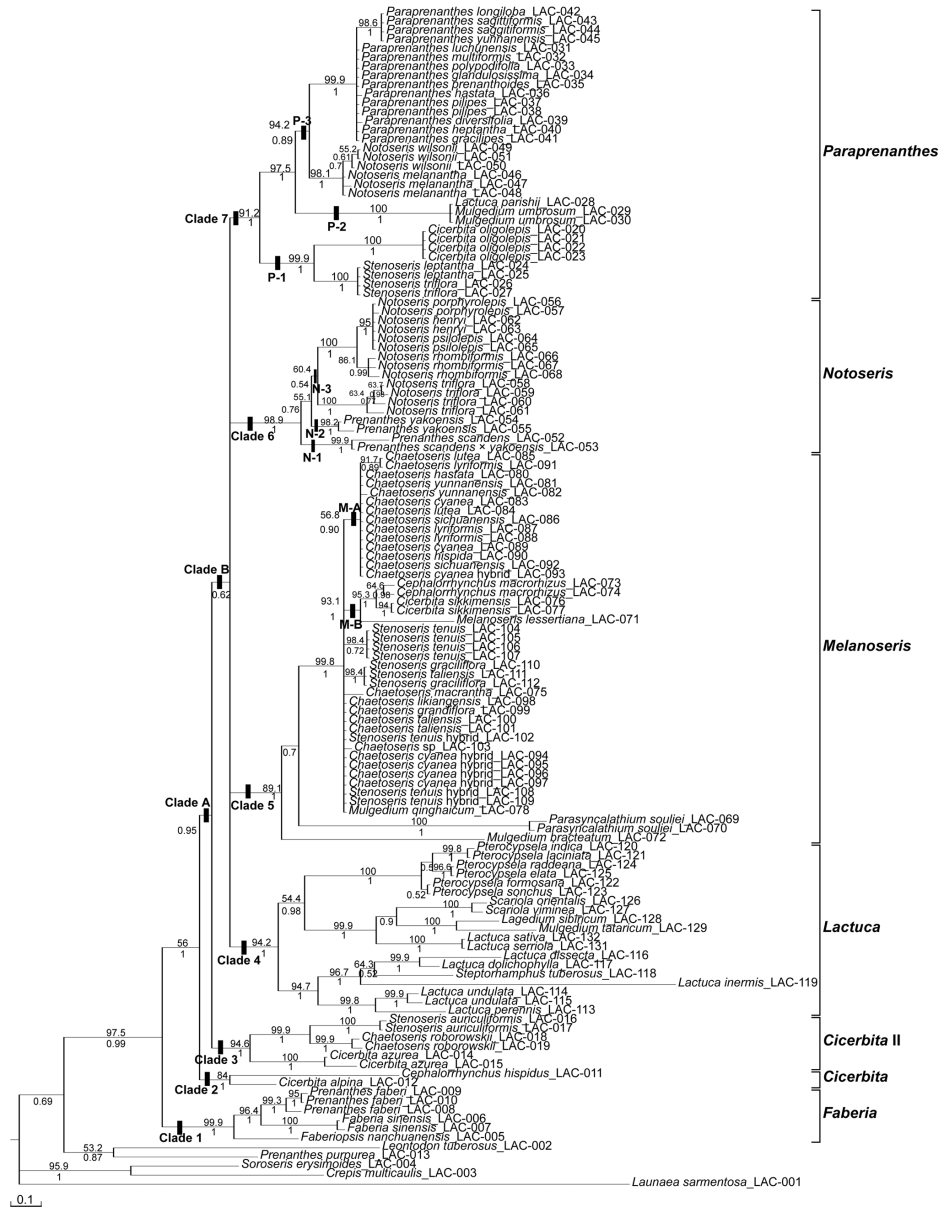
Bayesian phylogram (majority rule tree) of subtribe Lactucinae based on nrITS dataset including coded indels. Posterior probabilities (PP) are given below the branches, the jackknife support values (JK) of the corresponding Maximum Parsimony majority rule consensus tree above the branches. Reference point for the names of Chinese taxa is in general the morphology-based taxonomy of Shih (1997), whereas the clade names at the vertical bars on the right show our revised generic classification outlined in more detail and with the relevant synonymies in the Taxonomic conclusions.

#### Non-Coding Chloroplast Regions

The plastid matrix was of the same sample size and composition as the ITS region matrix. The length of the individual plastid markers ranged from 171 (with a unique large deletion in *Chaetoseris macrantha*) to 421 nt in *psbA-trnH*, to 929–998 nt in *trnQ^(UUG)^-5′rps16*. The length of the five combined plastid markers ranged from 3784–4028 nt. The full data are provided in Table 1.

Areas with uncertain homology classified as “hotspots” of sequence mutation according to Borsch & al. [41], mostly length-variable poly A/T-stretches, were excluded from the analyses. One exon (*petD*) and one hotspot were excluded from the *petD* region, five hotspots from *psbA-trnH*, one exon (*trnL*) and one hotspot from *5′trnL^(UAA)^-trnF*, eight hotspots from *rpl32-trnL^(UAG)^* and three hotspots from *trnQ^(UUG)^-5′rps16* (see Appendix S2). The length of the five combined plastid markers after exclusion of the hotspots ranged from 3619 to 3884 nt (see Table 1).

The final matrix of the *rpl32-trnL^(UAG)^* region comprised 154 parsimony informative characters without and 191 parsimony informative characters including the coded indels, having the highest phylogenetic performance among the five cp markers used (Table 1). It is followed by the *trnQ^(UUG)^-5′rps1*6 region, with 111 and including coded indels 129 parsimony informative characters. The smaller *psbA-trnH* region has a percentage of informative sites comparable to the *rpl32-trnL^(UAG)^* region, but excessive variation (even within species) rendered the alignment and homology confirmation partly difficult. *5′trnL^(UAA)^-trnF* had the lowest phylogenetic performance with 56 and including coded indels 66 parsimony informative characters. The final concatenated plastid matrix comprised 450 and including coded indels 545 parsimony informative characters.

**Figure 2:**
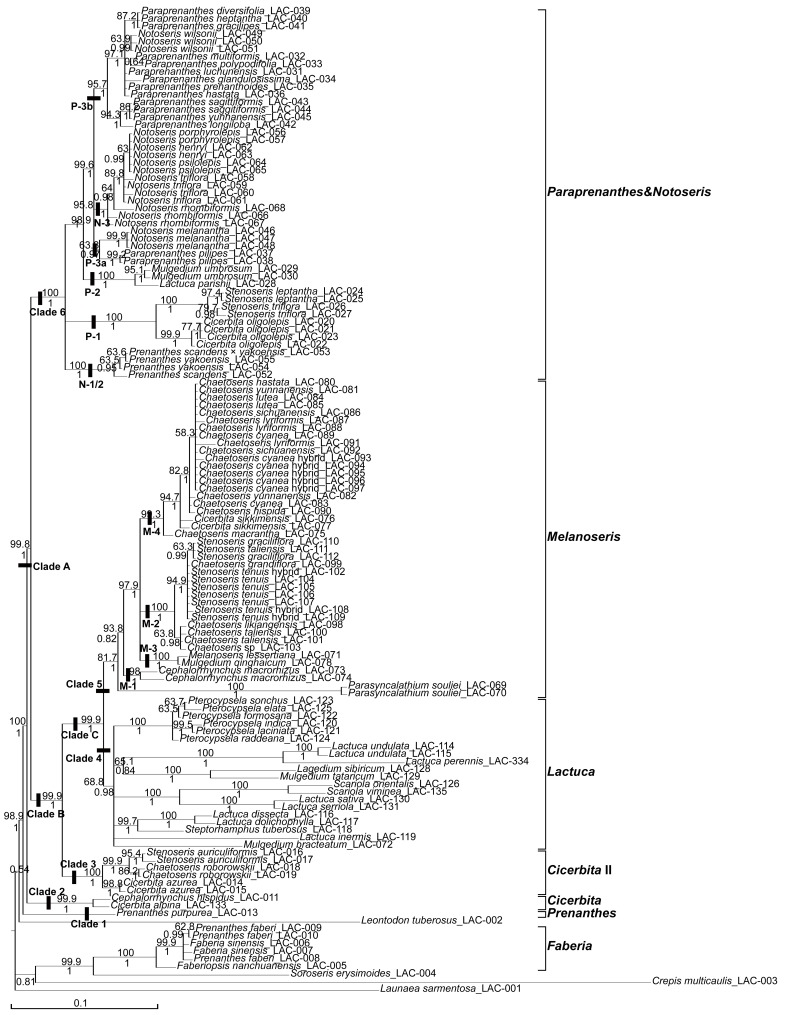
Bayesian phylogram (majority rule tree) of subtribe Lactucinae based on plastid dataset with coded indels. Posterior probabilities (PP) are given below the branches, the jackknife support values (JK) of the corresponding Maximum Parsimony majority rule consensus tree above the branches. Reference point for the names of Chinese taxa is in general the morphology-based taxonomy of Shih (1997), whereas the clade names at the vertical bars on the right show our revised generic classification outlined in more detail and with the relevant synonymies in the Taxonomic conclusions.

MP analyses were performed for both the individual cp markers and the concatenated plastid data set. The tree statistics are given in Table 1. MP analysis of the concatenated matrix resulted in 48 most parsimonious trees with L = 1342, CI = 0.847, RI = 0.950, RC = 0.805 (see Table 1). The resulting 50% MP consensus tree is congruent with the corresponding BI tree, apart from (a) two cases where smaller crown clades recognised in the MP tree collapsed in the BI tree, and (b) an incongruence in the relationship within the outgroup, where *Faberia* clustered in the BI tree with the two members of subtribe Crepidinae (i.e. *Crepis and Soroseris*), following *Leontodon* (Hypochaeridinae) as the nearest sister to the subtribe Lactucinae (incl. *P. purpurea*), while in the MP tree *Faberia* clustered only with *Soroseris*, in the closest position to Lactucinae, followed by *Leontodon* and *Crepis* as the successive sisters. We give here the BI phylogram (Fig. 2), with the MP Jackknife support (JK) values above and the BI posterior probability (PP) values below the branches.

### Phylogenetic Relationships

#### Its Phylogeny

Maximum Parsimony (MP) and Bayesian Inference (BI) analyses based on the ITS matrix both depict seven major lineages within a well supported (JK = 97.5, PP = 0.99) subtribe Lactucinae, which all have high statistical support, whereas deeper node have low or lack statistical support. **Clade 1** (JK = 99.9, PP = 1) comprises the genus *Faberia* in the circumscription of Shih & Kilian [13], thus including *Faberiopsis* and *Prenanthes faberi*; this *Faberia* clade is sister (JK = 56, PP = 1) to the remainder of the subtribe. **Clade 2** (JK = 84, PP = 1) is restricted to and unites the non-Chinese species providing the types of *Cephalorrhynchus* and *Cicerbita*; it is sister to a large clade A (JK<50, PP = 0.95) including clades 3–7 of the subtribe. The large **clade A** in turn comprises the clades 3 and B. **Clade 3** (JK = 94.6, PP = 1) included three species placed by Shih & Kilian [13] in *Cicerbita.* **Clade B** forms with low support (JK<50, PP = 0.62) the polytomous backbone of the Lactucinae, including clades 4–7. **Clade 4** (JK = 94.2, PP = 1) represents *Lactuca*, the type genus of the subtribe, among which the former *Lactuca* segregates *Pterocypsela, Steptorhamphus*, *Mulgedium* and *Lactucella* are nested. **Clade 5** (JK = 89.1, PP = 1) includes the types and most other species of the genera *Chaetoseris* and *Stenoseris,* among which the species providing the type of the old generic name *Melanoseris* is nested, and also the recently erected genus *Parasyncalathium.* **Clade 6** (JK = 98.9, PP = 1) comprises the Chinese near-endemic genus *Notoseris,* but excluding two of its members in the sense of Shih [12] or Shih & Kilian [13], which cluster in clade 7 with *Paraprenanthes.* **Clade 7** (JK = 91.2, PP = 1), finally, includes the Chinese endemic genus *Paraprenanthes* plus a few more species not considered by Shih [12] and Shih & Kilian [13] as members of that genus.

#### Plastid Phylogeny

MP and BI analyses of the combined plastid data set fully agree with respect to the phylogenetic relationships between and in the circumscription of the major lineages. They yielded six major lineages with mostly high statistical support, which are not all identical with those in the ITS phylogeny. Their relationships to each other also received high statistical support. The *Faberia* clade (JK = 100, PP = 1) is identical in circumscription to the corresponding clade in the ITS phylogeny, but here placed among the outgroup taxa clustered with *Soroseris* (JK = 99.9, PP = 1) and then *Crepis* (JK<50, PP = 0.81), which are members of the subtribe Crepidinae. The Lactucinae ingroup has high support (JK = 98.9, PP = 1), comprising clades 1–6. **Clade 1**, only comprising *Prenanthes purpurea*, is sister (JK = 100, PP = 1) to the remainder of the ingroup. **Clade 2** (JK = 99.9, PP = 1) is congruent to clade 2 of the ITS tree and is sister to a large **clade A** (JK = 99.8, PP = 1), which includes the remaining major lineages 3–6. Clades 3–5 are parts of a **clade B** (JK = 99.9, PP = 1), to which clade 6 is sister. **Clade 3** (JK = 100, PP = 1) is congruent to clade 3 of the ITS tree and sister to **clade C** (JK = 99.9, PP = 1), which comprises clades 4 and 5. **Clade 4** (JK = 68.8, PP = 0.98), comprising *Lactuca*, is congruent in circumscription but less so in internal topology with clade 4 of the ITS tree. **Clade 5** (JK = 81.7, PP = 1) is congruent in circumscription to clade 5, including *Melanoseris*, *Chaetoseris*, *Parasyncalathium* and *Stenoseris*, of the ITS tree, but has a somewhat different internal topology. **Clade 6** (JK = 100, PP = 1) finally, is congruent in circumscription to clades 6 *Notoseris* plus 7 *Paraprenanthes*, of the ITS tree, merging the taxa of these two clades in a different internal topology.

### Incongruences Between Nuclear And Plastid Phylogenies

The ILD test detected incongruence with high significance (P = 0.001) between the entire nuclear and plastid data sets as well as, in the calculation for the single clades, for the *Notoseris*, *Paraprenanthes* and *Melanoseris* clades. Therefore no analyses of a combined data set have been performed. While the ILD test is known to be overly sensitive in indicating conflicts between datasets [47], and alone therefore no sufficient proof for incongruence, its result in our case is fully corroborated by the high statistical branch support for the incongruent tree topologies (see MP Jackknife support values above and the BI posterior probability values below the branches in Fig. 1 and 2).

Incongruences between the two phylogenies with good to high branch support concern (1) the circumscription of the Lactucinae, (2) the relationships between major lineages, (3) assignment of taxa to major lineages, and (4) the internal topology of major lineages.

1. The circumscription of the subtribe Lactucinae is incongruent between the ITS and plastid trees: (a) the *Faberia* clade is sister to the remainder of the subtribe in the ITS tree (JK = 56, PP = 1, and JK = 97.5, PP = 0.99 for the Lactucinae including *Faberia*) but nested within the outgroup in the plastid tree (JK = 99.1, PP = 1 for the sister group relationship with *Soroseris*); (b) *Prenanthes purpurea* is nested in the outgroup in the ITS tree (JK = 97.5, PP = 0.99 for the ingroup without *P. purpurea*) but forms the first diverging branch of the Lactucinae in the plastid tree (JK = 98.9, PP = 1 for the sister group relationship with the core Lactucinae). Disregarding these two lineages, the Lactucinae are monophyletic in both phylogenies.
2. The most obvious incongruence in the relationships between the major lineages is that the *Notoseris* lineage (clade 6) and the *Paraprenanthes* lineage (clade 7) of the ITS phylogeny (JK = 98.9, PP = 1 for clade 6 and JK = 91.2, PP = 1 for clade 7) are represented in the plastid phylogeny by a single clade 6 (JK = 100, PP = 1) of different internal topology. In contrast, the topological incongruences in the relationships of these lineages to the other major lineages as well as in the relationships among these other major lineages are without statistical support, because the most major lineages are found along the polytomous backbone in the core of the subtribe in the ITS phylogeny.
3. In the single case of *Mulgedium bracteatum* (≡ *Melanoseris bracteata*), the assignment of a species to the major lineages is incongruent between the ITS and the plastid phylogeny. In our ITS tree focusing on Chinese Lactucinae this species is included in the *Melanoseris* clade with good statistical support (JK = 89.1, PP = 1) but in a fairly isolated position as the first diverging branch of that clade. In our plastid tree, in contrast, this species is nested in the *Lactuca* clade with moderate support (JK = 68.8, PP = 0.98), clustering therein with SW to E Asian members of *Lactuca* in a polytomous clade.
4. Incongruences in the internal topology occur (a) in the *Notoseris* and *Paraprenanthes* clades (clades 6 and 7 in the ITS phylogeny, parts of the single clade 6 in the plastid phylogeny), (b) in the *Lactuca* clade (clade 4 in both the ITS and plastid phylogeny), and (c) in the *Melanoseris* clade (clade 5 in both the ITS and plastid phylogeny). These are addressed in more detail in the Discussion.

## Discussion

Our phylogenetic reconstruction of the Lactucinae by molecular techniques is based on the most extensive sampling published for the subtribe to date. Our sampling, although focusing on the Chinese centre of diversity, spans the entire subtribe, including not only all genera present in China but also non-Chinese species providing the types of relevant generic names in the subtribe. We provide the first comprehensive phylogeny of this taxonomically difficult and controversial group and use this together with morphological data as basis for a revised generic classification of its members in the Chinese centre of diversity.

### Possible Causes Of Incongruence Between The Nuclear And Plastid Phylogenies

Technical causes, such as insufficient taxon sampling, long-branch attraction, sequencing errors, for the statistically well supported and thus “hard” topological incongruences [57] between our nuclear and plastid phylogenies, appear excludable in the light of our dense sampling, frequently with more than one sample per species, and the similar topologies obtained from both MP and BI analyses. Causes for these incongruences are judged with confidence therefore as essentially biological.

The nrITS sequences of our dataset appeared reliable (no pseudogenes) but we cannot exclude the possibility of divergent alleles among the multiple ITS copies within a nucleus [58]. In a few cases, additive polymorphism [45] seems in fact present among sequences of closely related taxa (see Table 2) and supports the hypothesis that nuclear introgression has taken place.

**Table 2:** Additive Polymorphic Sites (APS*) in the nrITS region sequences in four exemplar cases of putative introgressive reticulation.

Sample name in the tree	Positions of Additive Polymorphic Sites (APS) in the nrITS region sequence																
**1. ** ***Prenanthes scandens*** ** × ** ***yakoensis***	40	41	50	53	57	73	82	127	129	155	199	202	210	456	525	603	621
*Prenanthes scandens*_LAC-052	T	A	C	T	A	C	T	A	G	T	C	T	T	C	T	T	T
*Prenanthes scandens* × *yakoensis*_LAC-053	Y	W	Y	Y	R	Y	Y	R	R	Y	M	Y	K	Y	Y	Y	Y
*Prenanthes yakoensis*_LAC-054	C	T	T	C	G	T	C	A	A	C	A	C	G	T	C	T	C
*Prenanthes yakoensis*_LAC-055	C	T	T	C	G	T	C	R	A	C	A	C	G	T	C	C	C
**2. ** ***Stenoseris tenuis*** ** hybrid**	26	53	64	86	125	199	202	231	236	443	446	450	528	534	576	579	628
*Stenoseris tenuis*_LAC-104	C	T	G	C	T	T	T	T	T	C	T	A	T	C	C	C	T
*Stenoseris tenuis*_LAC-105	C	T	G	C	T	T	T	T	T	C	T	A	T	C	C	C	T
*Stenoseris tenuis* hybrid_LAC-108	Y	Y	R	Y	Y	Y	Y	Y	Y	M	Y	R	Y	Y	Y	Y	Y
*Stenoseris tenuis* hybrid_LAC-109	Y	Y	R	Y	Y	Y	Y	Y	Y	M	Y	R	Y	Y	Y	Y	Y
*Chaetoseris cyanea*_LAC-083	T	C	A	T	C	C	C	C	C	A	C	G	C	T	T	T	C
*Chaetoseris lyriformis*_LAC-088	T	C	A	T	C	C	C	C	C	A	C	G	C	T	T	T	C
**3. ** ***Chaetoseris cyanea*** ** hybrid**	14	26	64	86	123	443	446	450	554	565	579						
*Chaetoseris taliensis*_LAC-100	G	C	G	C	A	C	T	A	C	A	C						
*Chaetoseris taliensis*_LAC-101	G	C	G	C	A	C	T	A	C	A	C						
*Chaetoseris cyanea* hybrid_LAC-094	K	Y	R	Y	M	M	Y	R	Y	M	Y						
*Chaetoseris cyanea* hybrid_LAC-095	K	Y	R	Y	M	M	Y	R	Y	M	Y						
*Chaetoseris cyanea* hybrid_LAC-096	K	Y	R	Y	M	M	Y	R	Y	M	Y						
*Chaetoseris cyanea* hybrid_LAC-097	K	Y	R	Y	M	M	Y	R	Y	M	Y						
*Chaetoseris cyanea*_LAC-083	T	T	A	T	C	A	C	G	T	C	T						
*Chaetoseris lyriformis*_LAC-088	T	T	A	T	C	A	C	G	T	C	T						
**4. ** ***Notoseris melanantha***	26	53	55	82	120	164	195	200	462	596							
*Paraprenanthes pilipes*_LAC-037	C	C	T	C	G	G	G	G	T	G							
*Paraprenanthes pilipes*_LAC-038	C	C	T	C	G	G	G	G	T	G							
*Notoseris melanantha*_LAC-046	Y	Y	W	Y	R	R	R	R	K	R							
*Notoseris melanantha*_LAC-047	Y	Y	W	Y	R	R	R	R	K	R							
*Notoseris wilsonii*_LAC-049	T	C	A	C	G	G	G	G	T	G							
*Notoseris wilsonii*_LAC-051	T	C	A	C	G	G	G	G	T	G							

* An APS is recorded when at least one of the bases involved in a polymorphic site occurs separately at the same position in samples of putative parents.

doi:10.1371/journal.pone.0082692.t002

The sequences of the exclusively maternally inherited and thus non-recombining chloroplast genome come along with another drawback. This is the relatively high potential for interspecific cytoplasmic (chloroplast) gene flow, or chloroplast capture, also in absence of any nuclear gene flow, due to introgressive hybridisation [59]–[62] or even due to horizontal gene flow between sexually incompatible species [63]. Chloroplast capture is known from the Cichorieae even at intergeneric level [64] and is with or without incomplete lineage sorting [65]–[66] an important cause for incongruence between nuclear and plastid phylogenies in general.

#### Putative Cases Of Ancient Reticulation And Chloroplast Capture

The *Notoseris and Paraprenanthes* lineages, which form the well supported clades 6 and 7 along the polytomous backbone of the larger part of the subtribe in our nuclear phylogeny (Fig. 1), in contrast form the single joined clade 6 in the plastid phylogeny (Fig. 2). Notably, the first basally diverging branches of both lineages in the nuclear tree (N-1+ N-2 and P-1) appear as subclades N-1/2 and P-1 in the basal polytomy of the common clade in the plastid tree, while the core clades of both lineages in the nuclear tree appear as subclades of a second, later diverging polytomy in the plastid tree (compare Fig. 1 and 2). Only a few chromosome counts are known from species of the core clades of the two lineages, all indicating them to be diploids with 2n = 18 [67]. The only plausible explanation for this incongruence appears to us the assumption of an event of intergeneric reticulation with chloroplast capture already between ancestors of the current lineages. Early divergence of the basally branching subclades, along with geographical isolation and ecological separation through flowering time, may have led them accumulate sufficient chloroplast gene variation to be well distinguished from the remainder. The inner polytomous topology of both core clades of *Paraprenanthes* and *Notoseris* sensu Shih [12], in combination with their morphological homogeneity in each clade, may probably be ascribed to recent rapid radiation in a similar distributional area and ecological niche. The *Paraprenanthes umbrosa* subclade (P-2, represented in the trees by *Lactuca parishii* and *Mulgedium umbrosum*), is sister to the core *Paraprenanthes* clade P-3 in the ITS tree, but sister to the polytomous mixed *Paraprenanthes-Notoseris* core clade (including N-3 and P-3a+P-3b with different internal topology) in the plastid tree. This topology makes it likely that between the ancestors of the two core clades N-3 and P-3 further events of reticulation and cytoplasmic introgression may have taken place. With respect to the generic classification, we consider the nuclear phylogeny, which places *Notoseris* and *Paraprenanthes* in separate lineages, a better estimate of the taxon phylogeny because it is more in line with morphology.

A second putative case of ancient reticulation and chloroplast capture is exemplified by the entire genus *Faberia,* which appears in different subtribal placements in both trees (see under *Faberia* lineage., below). *Faberia* is alloploid with 2n = 34 [68]–[69], cytoplasmic gene flow was thus evidently accompanied by nuclear gene flow.

A third putative case of ancient reticulation constitutes the diploid *Prenanthes purpurea*. From morphological and cytological evidence it appears in this case very unlikely that the ITS tree represents the actual species phylogeny, whereas much more so that the plastid tree does (see under *Prenanthes purpurea* lineage, below).

#### Putative Cases Of Introgressive Hybridisation Between Extant Species

**Figure 3:**
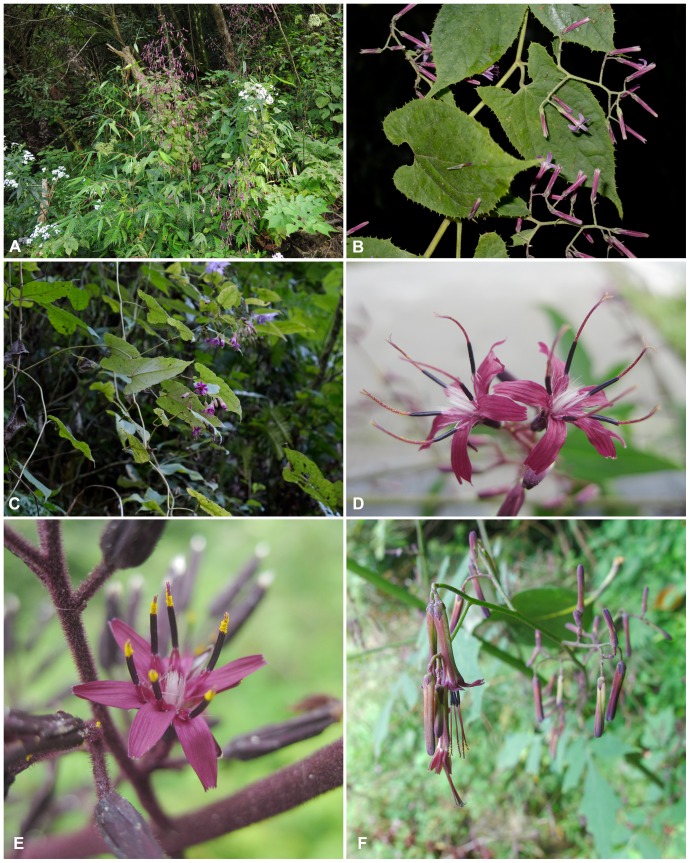
Selected species of *Notoseris* (A–C) and *Paraprenanthes* (D–F) in situ. A, *Notoseris henryi* (Sichuan, 9 Sep. 2013, photo by H. J. Dong; voucher: *H. J. Dong & al. 870* (KUN)), B. *N. scandens* (Yunnan, 11 Nov. 2011, photo by Y. Tang; voucher: *Z. H. Wang, L. Chen & Y. Tang 457* (KUN)), C. *N. yakoensis* (Yunnan, 11 Nov. 2011, photo by Y. Tang; voucher: *Z. H. Wang, L. Chen & Y. Tang 458* (KUN)), D. *Paraprenanthes wilsonii* (Sichuan, 25 Jun. 2011, photo by Z. H. Wang; voucher: *Z. H. Wang & L. Chen 344* (B, KUN)), E, *P. melanantha* (Sichuan, 2 Aug. 2011, photo by Z. H. Wang; voucher: *Z. H. Wang & L. Chen 489* (B, KUN)), F, *P. oligolepis* (Yunnan, 22 Sep. 2011, photo by G. X. Hu; voucher: *H. J. Dong & al. 416* (KUN)).

A rather clear example for incongruence indicating reticulation and cytoplasmic gene flow among extant species concerns the scandent species *Notoseris scandens* and *N. yakoensis* (see Fig. 3B–C; as *Prenanthes scandens and P. yakoensis* in the trees) in the *Notoseris* clade of the ITS tree and the joint *Notoseris-Paraprenanthes* clade in the plastid tree. The two species form a clade of their own in the ITS tree and consecutive sister clades in the plastid tree, but the morphologically intermediate accession designated as *Prenanthes scandens* × *yakoensis* clusters with *P. scandens* in the ITS tree (JK = 99.9, PP = 1), whereas with *P. yakoensis* in the plastid tree (JK = 63.6, PP = 0.95). Additive polymorphism [45] is present in the ITS sequence of the accession *P. scandens* × *yakoensis* (Table 2) and the putative hybrid population is morphologically clearly intermediate in the number and length of the inner phyllaries, the flower number per capitulum and the anthertube length. Since divergent ITS paralogues merged in a genome after a hybridisation event become homogenised by concerted evolution, the occurrence of a number of additive polymorphic sites (APS) supports a rather recent (as opposed to an ancient) introgression event. Based on these evidences, we hypothesise the formation of a natural hybrid population between *P. yakoensis* and *P. scandens*, with the former as its male parent and the latter as female parent, involving both plastid and nuclear introgression. The two scandent species typically grow at edges of montane forests to tall forb communities, e.g. along rivers, but found new habitats along roads through montane forests, which eventually helped formerly isolated populations of the two species to meet.

A second case is *Paraprenanthes melanantha* (Fig. 3E; as *Notoseris melanantha* in the trees). This species clusters with the morphologically closely allied *P. wilsonii* (Fig. 3D; ≡ *N. wilsonii*) with strong support (JK = 98.1, PP = 1) in the ITS tree, but with the widely distributed *P. sororia* (represented in the tree by its glandular hairy form that was treated as *P. pilipes* by Shih [12]), with lesser support (JK = 63.8, PP = 0.97) in our plastid tree. Additive polymorphism [45] in the ITS sequence of *P. melanantha* compared to *P. wilsonii* and *P. sororia* (Table 2) supports that cytoplasmic gene flow was accompanied in this case also by nuclear introgression. This could indicate that *P. melanantha* is hybridogenous with *P. wilsonii* as paternal and *P. sororia* as maternal parent. However, besides clearly additive polymorphic sites, we notice also polymorphic sites in *P. melanantha* that are not additive with respect to *P. sororia* and *P. wilsonii.* Moreover, and in contrast to the preceding case, morphologically, *P. melanantha* is not intermediate between the putative parental species but close to the paternal one, as both share an involucre with only 5 inner phyllaries (8 in *P. sororia*) and anther tubes without appendages >3 mm (not exceeding 1.6 mm in *P. sororia*). Presumably, in this case a more complex pattern of reticulation might have taken place and further studies are necessary to shed some light on it.

Other cases are addressed under the *Lactuca* (*Pterocypsela sonchus* and *P. elata*) and *Melanoseris* (*M. bracteata, M. graciliflora* and *M. tenuis, M. cyanea* group) lineages below.

### Monophyly And Circumscription Of Subtribe Lactucinae

Considering the joint evidence produced by the nuclear and the plastid phylogeny, subtribe Lactucinae is monophyletic only if the *Faberia* and *Prenanthes purpurea* lineages are disregarded. Otherwise its circumscription as a monophyletic entity depends on whether the nuclear or chloroplast phylogeny is followed.

***Faberia*** **lineage.** The genus *Faberia*, endemic to SW China, was included in *Prenanthes* and treated as a member of subtribe Lactucinae by Bremer [8] and Lack [9], but excluded by Kilian & al. [1] from the Lactucinae, in absence of DNA sequence data for morphological grounds only. Later Shih & Kilian [13] included *Faberia* (merged again with its former segregate *Faberiopsis*) in subtribe Lactucinae, based on our initial phylogenetic analysis of nrITS sequences. The position of *Faberia* in the nuclear tree, where it is placed with moderate statistical support (JK = 56, PP = 1) as sister to all other members of subtribe Lactucinae, and were the Lactucinae including *Faberia* received high support (JK = 97.5, PP = 0.99), is incongruent with its position in our plastid tree, where it is nested in the outgroup, with relative low support (JK<50, PP = 0.81) among the Crepidinae and within them as sister to the single *Soroseris* sample included (JK = 100, PP = 1). Liu & al. [68] have shown that *Faberia* has the chromosome number of 2n = 34, which is unusual in the Cichorieae and indicates an alloploid origin of the genus from parents with x = 8 and x = 9. Its incongruent positions in the nuclear and plastid trees make a reticulation with a maternal ancestor of the genus from the Crepidinae and a paternal ancestor from the Lactucinae the most likely scenario, but a plastid phylogeny with a much more extensive sampling would be necessary to asses its potential maternal ancestor. Whether the nuclear or chloroplast phylogeny provide the better phylogenetic estimate for the genus is difficult to assess, because morphology is little decisive in this case. The assumed sudden and rapid diversification of tribe Cichorieae in its evolutionary history [1],[3], might be an explanation that clear synapomorphies are frequently missing for the major lineages recognised as subtribes [8]. This applies especially to subtribes Lactucinae and Crepidinae, and certainly is the major reason for their late recognition as separate lineages. Bremer [8] identified for the Crepidinae an involucre distinctly differentiated between inner and outer phyllary series (typically so in e.g. *Youngia* and *Ixeris*) as a possible synapomorphy. In fact, in the Lactucinae often the outer phyllary series grade into the inner ones (e.g. often so in *Lactuca* and *Melanoseris*), but *Notoseris* and *Paraprenanthes*, e.g., have distinctly separated inner and outer phyllary series, as present also in *Faberia*. Morphological reasons for both placements of *Faberia* can be found according to our current knowledge. For classification purposes, we follow, for the time being, the nuclear DNA phylogeny and hence treat *Faberia* as a member of subtribe Lactucinae.

The revised circumscription of *Faberia* as a genus of seven species endemic to China given by Shih & Kilian [13], with re-inclusion of the former segregate *Faberiopsis* and inclusion of *Prenanthes faberi,* is fully corroborated by both our nuclear and plastid phylogenies.

#### Prenanthes Purpurea Lineage

*Prenanthes purpurea* L., a chiefly European species, provides the type of the generic name *Prenanthes.* Kilian & Gemeinholzer [11] and Kilian & al. [1] stated that this genus should probably be considered as monospecific, because the many other species formerly included seem unrelated to *P. purpurea*. This hold true also for the seven Chinese species maintained as members of *Prenanthes* by Shih [12],[22]: four of them (*P. angustiloba*, *P. leptantha*, *P. macrophylla* and *P. tatarinowii*) were found to belong actually to subtribe Crepidinae [2],[13]–[14]; among the three remaining species, one, *P. faberi*, is nested in our analyses in *Faberia*, and two, *P. scandens* and *P. yakoensis,* in the *Notoseris* clade of the ITS tree or the *Notoseris-Paraprenanthes* clade of the plastid tree, respectively (Fig. 1–2).

In the nrITS trees published, *Prenanthes purpurea* is placed far distant from the Lactucinae [70] and clusters instead with the subtribe Hypochaeridinae [1],[71] as in our ITS tree. This placement is meanwhile supported by ITS sequences of four different accessions but is surprising because *P. purpurea* and the Hypochaeridinae are morphologically entirely unrelated: *P. purpurea* has cyanic flowers (instead of always yellow or, rarely, white flowers in the Hypochaeridinae), pendent (instead of usually erect) flowering capitula, a pappus of scabrid (instead of almost always stiffly fimbriately plumose) bristles. They also do not agree cytologically. *P. purpurea* is diploid with x = 9 [72], while the basic chromosome numbers in the Hypochaeridinae range from x = 3 to x = 7 with a single exception of x = 11 [71].

In contrast to the ITS phylogeny, *Prenanthes purpurea* has a basally branching position in our plastid phylogeny, being sister with full support (JK = 100, PP = 1) to all other genera of the Lactucinae except *Faberia.* The same has been indicated, but without statistical support, in a previous *matK* tree [1]. Both from morphology and cytology, *P. purpurea* would in fact best fit into subtribe Lactucinae. Pending further studies to elucidate the causes for the incongruent molecular results, it would be appropriate either to include it, with reservations, in the Lactucinae, or else to leave *Prenanthes* unassigned to a subtribe, instead of placing it into the Hypochaeridinae.

#### Core Lactucinae

Our analyses, which include (a) all major lineages of the subtribe Lactucinae, (b) all species groups present in China, and (c) also the species providing the types of the relevant generic names established in the subtribe, revealed congruently in the nuclear and plastid phylogenies a core of the subtribe comprising six (five in the plastid phylogeny) major lineages, of which five (four) are present in its Chinese centre of diversity (Fig. 1–2): (1) the *Cicerbita* lineage, (2) the *Cicerbita* II lineage, (3) the *Lactuca* lineage, (4) the *Melanoseris* lineage, (5) the *Notoseris* lineage, and (6) the *Paraprenanthes* lineage, the last two revealed as a single clade in the plastid phylogeny.

Relationships of the major lineages within the core Lactucinae can be inferred from our analyses with some caution only, because of the lacking resolution for the deeper nodes in the ITS tree. Good support, however, is received for the sister group relationship of the *Cicerbita* lineage to the remainder of the core Lactucinae in both phylogenies (JK<50, PP = 0.95 in the ITS tree; JK = 99.8, PP = 1 in the plastid tree, see Fig. 1–[2]). The relationship of the *Cicerbita* II lineage is incongruent in both datasets: in the ITS phylogeny it is sister with low support in the BI tree (JK <50, PP = 0.62, relationship unresolved in the MP tree) to the unresolved remainder of the core Lactucinae, whereas in the plastid phylogeny it is sister to a clade comprising the *Lactuca* and *Melanoseris* lineages with almost full support (JK = 99.9, PP = 1). Considering the weak support through the ITS dataset, this incongruence should be regarded as soft and rather the sister group relationship of the *Cicerbita* II lineage to the *Lactuca + Melanoseris* lineages favoured as hypothesis. Inferred from the plastid tree, the *Notoseris* and *Paraprenanthes* lineages may be regarded as sisters, which are in turn sister to the *Cicerbita II* + *Lactuca + Melanoseris* lineages.

Hence, the following hypothesis on the evolution of the subtribe Lactucinae may be proposed: the ancestors of the mesic European-SW Asian *Cicerbita* lineage have, on the one hand, migrated towards eastern Asia giving rise to the mesic *Notoseris* and *Paraprenanthes* lineages and, on the other hand, migrated north- and northeastwards across Eurasia to North America as well as south- and southeastwards into Africa and S Asia, giving rise to the mesic to xeric *Cicerbita* II, *Lactuca* and *Melanoseris* lineages.

Molecular clock calculations estimate the age of the most recent common ancestor of subtribe Lactucinae, as the youngest branch of the core group of tribe Cichorieae (clades 4 and 5 according to Kilian & al. [1] and [3]), to be c. 15–4 Ma [2]–[3],[14], thus spanning the Middle Miocene to Early Pliocene. This period is characterised by significant tectonic events, such as the uplift of the Qinghai-Xizang Plateau in Asia, the southern part of which reached its present elevation by c. 15 Ma [73] with larger impact on climate and vegetation.

### Phylogeny Of The Major Lineages Of The Core Lactucinae

#### Cicerbita Lineage

The *Cicerbita* lineage, in our study represented by the type species of the generic names *Cicerbita* and *Cephalorrhynchus* (both species with a chromosome number of 2n = 18 [72]), constitutes the oldest diverging branch of the core Lactucinae. Since *Cephalorrhynchus* is part of this lineage, it can be treated as congeneric with *Cicerbita*. None of the Chinese members of the subtribe included in our study is part of this clade. Altogether twelve species have been classified in the two genera by Shih [12] or in *Cicerbita* by Shih & Kilian [13], respectively. Four of them, from N China, are not included in the present study (compare Shih & Kilian pp214–215 [13]), but the eight species included are all nested either in the *Cicerbita* II (CII) clade, the *Melanoseris* (M) clade or the *Paraprenanthes* clade (P); these are: *Cephalorrhynchus albiflorus*, *C. macrorhizus* and *C. saxatilis* (M), *Cicerbita azurea* (CII), *C. sikkimensis* (M) and *C. oligolepis* (P) of Shih [12], and *Cicerbita auriculiformis*, *C. azurea* and *C. roborowskii* (CII) of Shih & Kilian [13].

*Cicerbita*, established as early as 1822 by Wallroth, appeared vaguely defined right from the beginning, including eight, partly very different species, and soon came in competition with *Mulgedium,* established for a similar heterogenous assemblage of species by Cassini in 1824, which then displaced the name *Cicerbita* during the 19th century. Through the revision by Beauverd [21], where the name *Cicerbita* was taken up again, it received its widest circumscription in the history of Lactucinae systematics, diagnosed solely by a pappus composed of an outer row of minute hairs and an inner row of bristles. Later, this feature was characterised by Stebbins [74] as similar useless for generic delimitation as the presence or absence of an achene beak, because it separates species that are closely allied beyond any doubt. It was, however, still employed, e.g. by Tuisl [16] to delimit the genera *Cephalorrhynchus*, *Cicerbita* and *Steptorhamphus* with an outer row of minute hairs from *Lactuca, Mulgedium* and *Scariola* without such an outer row (see below). Stebbins [74], in an initial attempt to redefine *Cicerbita,* in contrast established the narrowest circumscription of the genus, containing only three species, *C. alpina*, *C. pancicii* (Vis.) Beauverd and *C. abietina* (Boiss.) Stebbins, that all have columnar achenes with 5 equal main ribs, coarse pappus hairs and a *C. alpina* habit. A revised concept of the genus will be provided by Kilian & al. (unpublished data).

#### *Cicerbita* Ii Lineage

Based on our initial ITS phylogeny with largely unresolved relationships of the major lineages, Shih & Kilian [13] assigned an assemblage of seven, mainly N Chinese species, comprising one species with certain affinity and three very little known species with assumed affinity to *Chaetoseris roborowskii* (*≡ Cicerbita roborowskii*), plus *Cicerbita azurea* and *C. tianschanica*, tentatively to *Cicerbita*. It is clear from our analyses, which represents three species of this assemblage (the species pair *Cicerbita auriculiformis* and *C. roborowskii*, plus *C. azurea*), that they constitute a separate lineage clearly distant from *Cicerbita*. Whether the remaining species of that assemblage share this positions, has still to be seen. Study of the type material of *Chaetoseris rhombiformis*, treated as a member of *Melanoseris* by Shih & Kilian [13], revealed that it is actually conspecific with *C. roborowskii*. The phylogeny of this predominantly Central Asian lineage, as well as its circumscription, nomenclature and classification will be treated in a consecutive paper on the global phylogeny and systematics of subtribe Lactucinae (unpublished data).

#### *Lactuca* Lineage

*Lactuca* is not only the name-giving genus of the Lactucinae, its circumscription and delimitation is also crucial for the generic classification of the subtribe. Its circumscription varied extraordinarily in the history of the systematics of the *Lactuca* alliance. An extremely broad concept of *Lactuca* was introduced by Bentham [75] and maintained by Hoffmann [5], not only spanning most of the known diversity of the entire present-day subtribe but even including genera and species today placed into subtribes Crepidinae and Hyoseridinae. Very narrow concepts, in contrast, were advocated, in particular, by Tuisl [16] and Shih [23]–[24], who generically separated a number of elements from the core of *Lactuca.* Moderately wide concepts were established by Stebbins [74],[76]–[77] and Ferákova [78].

The genus has never been revised in its entirety, and all four last mentioned authors only dealt with regional subsets of the genus. Because of its economic importance, many studies and also the first molecular studies [70],[79]–[80] focused on the lettuce “gene pool” [81], which constitutes the core of *Lactuca.* Koopman & al. [70] provides the only molecular phylogeny of the genus available to date and is based on nrITS1. The results of their analysis are corroborated by our phylogeny based on the entire nrITS region and a small but representative sampling of *Lactuca.* Three well supported major clades are revealed: (1) One (JK = 99.9, PP = 1) comprises the lettuce, *Lactuca sativa*, which provides the type of the generic name, as well as its primary, secondary and tertiary gene pool [70]. Their distribution is centred in Europe, the Mediterranean and SW Asia and all are diploids with 2n = 18. This clade includes the type species of the segregates *Scariola* (*S. viminea* ≡ *Lactuca viminea*), *Mulgedium* (*M. runcinatum = Lactuca tatarica*) and *Lagedium* (*L. sibiricum* ≡ *Lactuca sibirica*). (2) The second (JK = 100, PP = 1) clade comprises the E Asian *Lactuca indica* and its relatives, which were generically separated from *Lactuca* by Shih [23] as *Pterocypsela*. This clade is the dominant representative of the genus in E Asia and replaces the first clade there. Its entire species are likewise diploid with 2n = 18. Both clades together form a clade with less statistical support (JK = 54.4 PP = 98) than the individual clades have themselves. (3) The third clade (JK = 94.7, PP = 1) in turn is sister to the former two clades and has the highest number of species, of which only few are represented in our study. In contrast to the first two clades, it comprises subclades with chromosome numbers of 2n = 18, 2n = 16 and 2n = 34, the last one apparently by alloploidisation. Its members have a pappus with an outer ring of minute hairs or not, while all members of the first two clades uniformly lack such an outer ring. It includes the type species of the segregates *Steptorhamphus* (*S. tuberosus* ≡ *Lactuca tuberosa*) and *Lactucella* (*L. undulata* ≡ *Lactuca undulata*), the Asian *L. dissecta* and *L. dolichophylla*, both also present in China, the widespread African *L. inermis* Forssk. ( = *L. capensis*), *L. perennis* L. and, not shown here, other European, Mediterranean and SW Asian species as well as the group of native North American species with a chromosome number of 2n = 34 ([72]; unpublished data).

Our plastid tree, which is the first one with a selection of *Lactuca* species published, reveals a polytomy of six clades. Differences to the topology of the ITS tree are: (a) the *L. sibirica-L. tatarica* subclade clusters with the *L. perennis* clade, although with weak support (JK = 65.1, PP = 0.84), but not with the *L. sativa* clade. This is less consistent with the hybridisation experiments reviewed by Koopman & al. [70], which place *L. tatarica* into the secondary lettuce gene pool because it produces fertile hybrids when somatically hybridised with *L. sativa*, but place *L. perennis* outside the lettuce gene pool because it is not crossable with *L. sativa* primary gene pool species. (b) *L. inermis* is not nested in the *Steptorhamphus tuberosus-L. dissecta* clade but constitutes a branch of its own. (c) As noted already above, *Mulgedium bracteatum* of the *Melanoseris* lineage in the ITS tree is nested here in the *Lactuca* lineage as a further separate branch.

While the ITS tree is inconclusive with respect to the relationship of the *Lactuca* lineage with other major lineages in the core Lactucinae, the plastid tree indicates a highly supported sister group relationship (JK = 99.9, PP = 1) between the *Lactuca* and the *Melanoseris* lineages. The two lineages themselves only receive moderate support, *Lactuca* (JK = 68.8, PP = 0.98) still less than *Melanoseris* (JK = 81.7, PP = 1). Exclusion of *M. bracteatum* from the analysis does not affect the statistical support of either lineage and since *M. bracteatum* is a diploid species (2n = 16 [72]), the reason for its incongruent position may perhaps be chloroplast capture through introgressive hybridisation. A sister group relationship with *Melanoseris* is also supported by morphology, where differences between the two lineages are particularly difficult to define.

*Lactuca* is a suitable example to elucidate the shortcomings of the previous classification attempts in the *Lactuca* alliance with the molecular phylogenetic results. Although it is evident that the achene as dispersal unit faces a particularly strong exposure to selection pressure and corresponding morphological changes affecting their functionality [82], a very static, sometimes even typological, understanding of achene features has often enough pervaded the taxonomy of the *Lactuca* alliance. Absence of a true achene beak and a weakly compressed achene body were the main features for the separation of *Mulgedium* (*L. tatarica* and *L. sibirica* [16], somewhat altered concept by Shih [23]) or *Lagedium* (including *L. tatarica* and *L. sibirica* [83] or *L. sibirica* only [23]); the combination of a compressed achene body, winged lateral ribs and a beak justified the separation of the E Asian *Lactuca* lineage as *Pterocypsela* [23], and the apomorphy of two rod-like, pendent basal appendages at the long-beaked achene apex justified separation of *L. undulata* as monotypic genus *Lactucella* [84]. A relict of 19th classification, where schematically pappus features were in use for classification at generic and higher ranks, is the use in the *Lactuca* alliance of the absence of an outer row of minute hairs in the pappus to delimit *Lactuca* from *Steptorhamphus* as well as from, in particular, *Cephalorrhynchus* and *Cicerbita* [5],[16],[21],[23],[25],[78]. The *Steptorhamphus tuberosus-L. dissecta* clade is an example, where even a single, well supported clade, revealed both in the nuclear and plastid phylogenies, unites members having a pappus with (*S. tuberosus*) and without (*L. dissecta, L. dolichophylla*) an outer ring of minute hairs. The segregation of the *L. viminea-L. orientalis* species group as *Scariola* for the low number of 4 or 5 flowers per capitulum along with white stems and adnately decurrent leaves, in contrast, appears morphologically much more plausible, yet is equally unsubstantiated in the light of the molecular phylogenetic results. All these former segregates are deeply nested in *Lactuca* according to both the nuclear and plastid phylogenies.

Among the E Asian *Lactuca indica* species group, different species concepts, which depend on the evaluation of conspicuous leaf shape differences found, have been applied recently and consequently different numbers of species recognised. Whereas Shih [12],[23] recognised seven species (under *Pterocypsela*), Shih & Kilian [13] reduced them to only four, considering the otherwise similar plants with entire-leafy and pinnately lobed leaves only as extremes of infraspecific ranges of variation. The latter authors therefore sunk *L. elata* (with entire leaves) into *L. raddeana* (with lyrately or pinnately lobed leaves), *L. laciniata* (with strongly pinnately lobed leaves) into *L. indica* (with mostly entire leaves) and *L. sonchus* (with entire leaves) into *L. formosana* (with strongly pinnately lobed leaves). Using the narrower species concepts in our analyses, which includes all species of the group but *L. triangularis*, both phylogenies link with high support *L. laciniata* and *L. indica* (JK = 99.8, PP = 1 in ITS tree; JK = 99.5, PP = 1 in plastid tree). The ITS phylogeny also links with high support *L. elata* and *L. raddeana* (JK = 96.6, PP = 1), only *L. sonchus* and *L. formosana* are linked with weak support (JK <50, PP = 0.52). The plastid phylogeny in contrast links *L. elata* with *L. sonchus* and *L. formosana* with weak support (JK = 63.5, PP = 1). These results in combination with the low amount of sequence variation involved among the six *Pterocypsela* samples (12 variable sites, 11 informative in the nuclear data set; 10 and 6 informative in the plastid data set) can be seen as an additional support for the hypothesis of wide ranges of infraspecific leaf shape variation and consequently wider species concepts at least in the first case, while the other cases deserve further studies because of and also with respect to the ambiguous position of *L. elata.*

#### Melanoseris Lineage

The genus *Melanoseris* (for exemplar species see Fig. 4) was established by Decaisne in 1843 to include two species from the Himalayas, which are now treated as a single species, *M. lessertiana*. It was considered to differ from *Cicerbita* (then under the name *Mulgedium*) because of its beaked achenes and from *Lactuca*, because of its pappus with an outer series of minute hairs. Edgeworth [85] added a few more Himalayan species, which we confirm to belong to this lineage, but afterwards the use of the name *Melanoseris* was abandoned. The name was only recently revived by Shih & Kilian [13] for this lineage, based on our initial ITS phylogeny, through which it became evident that the types of the newly established genera *Chaetoseris* and *Stenoseris* by Shih [25] are part of one lineage with *M. lessertiana*, which provides the type of the name *Melanoseris* and was treated by Shih [12] under *Mulgedium*. Shih’s genera *Chaetoseris* and *Stenoseris* are, moreover, shown in our analyses to be actually bi- and triphyletic, respectively (Fig. 1–2). Apart from the bulk of the *Chaetoseris* species nested in the *Melanoseris* lineage, one species, *C. roborowskii* (including also *C. rhombiformis*), is nested in the *Cicerbita* II lineage. *Chaetoseris* was circumscribed and delimited from *Lactuca* and *Cicerbita* by the combination of beaked achenes, an achene corpus with broad, thickened lateral ribs and a pappus with an outer series of minute hairs [25]. Shih’s six *Stenoseris* species are distributed among the *Melanoseris* lineage (*S. graciliflora*, *S. taliensis*, *S. tenuis*), the *Paraprenanthes* lineage (*S. leptantha, S. triflora*) and the *Cicerbita* II lineage (*S. auriculiformis*). *Stenoseris* was circumscribed by the combination of narrowly cylindrical, 3-flowered capitula, an achene corpus with broad, thickened lateral ribs and a pappus with an outer series of minute hairs [25]. All features used to circumscribe the two genera are clearly shown to be homoplastic. It is therefore not surprising that, compared to Shih [12],[25], the *Melanoseris* lineage, moreover, includes all species of *Cephalorrhynchus* (distinguished by Shih through the achene corpus lacking thick, broadened lateral ribs), one of *Cicerbita* (*C. sikkimensis*) and three species of *Mulgedium* (distinguished by Shih through the pappus lacking an outer row of minute hairs, *M. bracteatum*, *M. lessertianum* and *M. monocephalum*).

**Figure 1:**
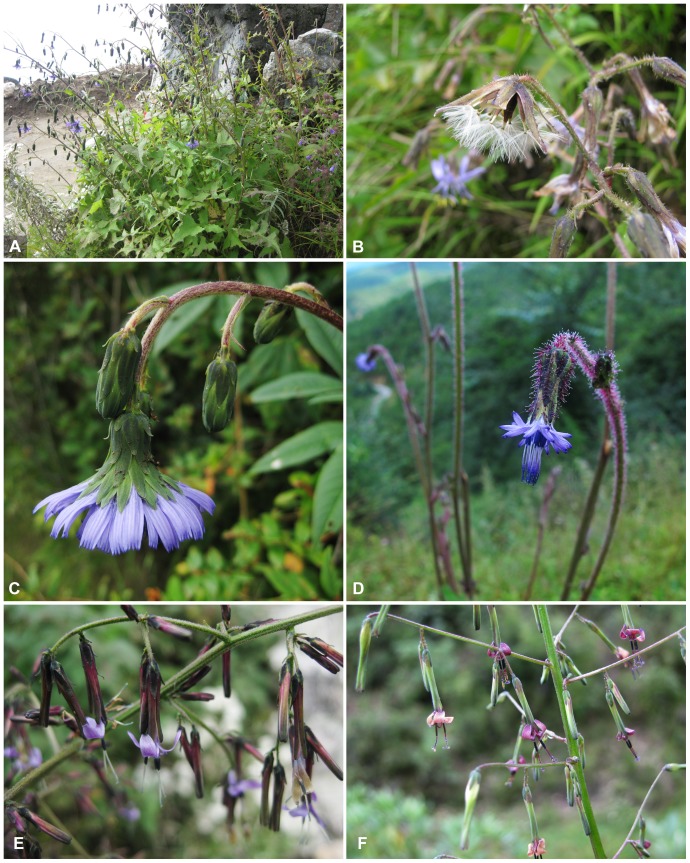
Selected species of *Melanoseris* in situ. A and C, *Melanoseris atropurpurea* (Yunnan, 9 Sep. 2009, photo by Z. J. Yin; voucher: *Z. J. Yin & al. 1970* (KUN)), B. *M. likiangensis* (Sichuan, 23 Aug. 2012, photo by N. Kilian; voucher: *N. Kilian & al. 10808* (B, KUN), D. *M. cyanea* (Yunnan, 22 Sep. 2011, photo by G. X. Hu; voucher: *H. J. Dong & al. 446* (KUN), E, *M. tenuis* (Yunnan, 10 Sep. 2009, photo by Z. J. Yin; voucher: *Z. J. Yin & al. 1969* (KUN)), F, *M. graciliflora* (Sichuan, 19 Aug. 2012, photo by N. Kilian; voucher: *N. Kilian & al. 10509* (B, KUN).

*Melanoseris* constitutes a large, well supported lineage (JK = 89.1, PP = 1 in the ITS phylogeny, JK = 81.7, PP = 1 in the plastid phylogeny). Most species, in particular all its Sino-Himalayan members, are diploid with 2n = 16 ([72]; under *Chaetoseris* and *Stenoseris* [86]), otherwise a number of species also has 2n = 18 (unpublished data). Besides the Sino-Himalayan species, *Melanoseris* also includes S, SW and Middle Asian as well as African species (unpublished data), but our present sampling is restricted chiefly to the species occurring in China. *Mulgedium bracteatum*, which appears in the ITS tree of the global sampling (unpublished data, there also with a second sample) within a further basally branching clade of SW and Middle Asian species, therefore takes an isolated, basally branching position in the present ITS tree. The strikingly incongruent position in the plastid phylogeny as a member of the *Lactuca* lineage deserves further investigation. From the morphological evidence we consider the nuclear phylogeny as the better estimate for the species phylogeny.

The next following branch, congruently revealed by the nuclear and plastid phylogeny, is sister with robust support (JK = 99.8, PP = 1 in the ITS phylogeny; JK = 93.8, PP = 0.82 in the plastid phylogeny) to all other Sino-Himalayan species of the lineage and consists of *Parasyncalathium souliei* only. Originally described as *Lactuca souliei* in 1895, the attractive bright blue-flowered acaulescent alpine species was placed together with habitually and ecologically strikingly similar species in *Lactuca* sect. *Aggregatae*, which later became the separate genus *Syncalathium*. Stebbins (pp47–50 [87]) inferred from achene morphology, Zhang & al. [88] from karyology, and Kilian & al. (pp348–350 [1]) and Zhang & al. [2],[14] from molecular phylogeny, all provided evidence that *L. souliei* is entirely unrelated to the other species of *Syncalathium* and that their overall similarity is hence a result of convergent evolution, presumably in response to the environmental changes following the uplift of the Qinghai-Xizang Plateau. Kilian & al. [1] recognised the species as a member of subtribe Lactucinae rather than of Crepidinae, to which S*yncalathium* belongs to, and Shih & Kilian [13] later placed it into *Melanoseris*, while Zhang & al. [14], arguing with its peculiar morphology, accommodated it in their newly established genus *Parasyncalathium*. Our analyses presented here do not provide unambiguous support for either classification. For the time being, we prefer to maintain its inclusion in *Melanoseris.*

The bulk of the Sino-Himalayan species all appear in a large polytomy in the ITS phylogeny, with only two subclades that comprise samples of more than one species (Fig. 1: M-A and M-B). The plastid phylogeny provides higher resolution for the lineage and shows four major subclades with well support (Fig. 2: M-1 to M-4). None of the subclades that comprise samples of more than one species, however, is fully congruent with either subclade revealed in the ITS tree.

1. *Melanoseris cyanea* group: The larger of the two subclades of the ITS tree (clade M-A) includes all but four samples (as *Chaetoseris cyanea* hybrid_LAC094-097 in the tree) that belong to the *M. cyanea* group of clade M-4 in the plastid phylogeny. The core of the group congruently revealed in both phylogenies contains a number of taxa, morphologically clearly allied to *M. cyanea* (Fig. 4D). Morphological variation within this group of robust tall forbs in particular regards indumentum, leaf shape, size of capitula and number of flowers per capitulum, flower colour, and length of the anther tube. Delimitation of taxa is very problematic due to a lack of clear morphological discontinuities. These may, however, be the results of previous areal changes with subsequent events of hybridisation and introgression, processes that are apparently still ongoing. Notably, the aforementioned four sympatric samples (as *Chaetoseris cyanea* hybrid_LAC094–097 in the tree) of the *M. cyanea* group, which fall into the large polytomy of the Sino-Himalayan species in the ITS phylogeny, have a number of additive polymorphic sites in their ITS sequences (Table 2), indicating the occurrence of still divergent, non-homogenised ITS paralogues likely as a result of nuclear gene flow, and this finding corresponds to the presence of intermediate morphological characters states, because of which these samples do not match either of the species distinguished and were therefore designated as putative hybrids. Even the already widened species concepts by Shih & Kilian [13], compared to Shih [12], do not work when confronted with the variation actually encountered in the field across the distribution area of the *M. cyanea* group in China. The lacking molecular resolution within this group thus corresponds well to the lack of morphological discontinuities and makes further taxonomic adjustments necessary (see Taxonomic conclusion).
2. *Melanoseris macrorhiza* group: In the ITS phylogeny *M. macrorhiza* (≡ *Cephalorrhynchus macrorhizus* in Shih 1997) clusters together with *M. violifolia* ( = *Cicerbita sikkimensis* in Shih 1997) and *M. lessertiana* (providing the type of *Melanoseris*) in a well supported (JK = 93.1, PP = 1) clade (Fig. 1: clade M-B). In the plastid phylogeny, in contrast, this clade does not exist at all but the three species occur in three different clades (Fig. 2: M-1, M-3, M-4). *M. lessertiana* instead forms a clade with full support (JK = 100, PP = 1) together with *M. qinghaica* (≡ *Mulgedium qinghaicum* [89]). *M. qinghaica* actually represents *Mulgedium lessertianum* in the sense of Shih [12] and the Chinese populations of *Melanoseris lessertiana* in the sense of Shih & Kilian [13], and replaces entirely the latter species in China. In the ITS tree *M. qinghaica* forms a separate branch within the large polytomy of the Sino-Himalayan species. Morphologically *M. lessertiana* and *M. qinghaica* have apparent close affinities to each other and are mainly distinguished by the distinctly longer achene beak and very short anthertube of *M. qinghaica*. It thus appears that the plastid phylogeny in this respect is more in line with morphology. Inferred from morphology, however, all four aforementioned species are considered to be more closely related to each other, as is revealed in the ITS tree for three of them. They are all rather low growing herbs usually without a dominant main stem.
3. *Melanoseris graciliflora* group: The morphologically closely allied, few-flowered species pair *M. graciliflora* (Fig. 4F; *Stenoseris graciliflora* [12]) and *M. tenuis* (Fig. 4E; *Stenoseris tenuis* [12]) is nested in the plastid phylogeny (Fig. 2: clade M-2) in a clade together with several species having capitula with many to numerous (*M. atropurpurea*, Fig. 4A+C) flowers and usually clasping stem leaves. All are robust tall forbs with cyanic flowers. In the ITS tree the members of this clade all form separate branches in the large polytomy except for the multiple samples of *M. graciliflora* and *M. tenuis*. Morphology makes this clade in the plastid tree neither obvious nor unlikely, at least if we accept also more drastic changes in the flower number per capitula as a common trend in character evolution, what we certainly have to do. We may hence accept the inferred relationship as a hypothesis for further studies, but also taking into consideration that relationships in Sino-Himalayan *Melanoseris* may be blurred by events of hybridisation and introgression. A number of well detected additive polymorphic sites in the ITS sequences of *Stenoseris tenuis* hybrid_LAC-108 and 109 (Table 2), plus the intermediate morphological characters (especiall the number of inner phyllaries), in combination with the first author’s observation in the field that some typical plants of *M. cyanea*, *M. tenuis* and *M. atropurpurea* co-occurred in the same habitat, all indicates introgressive hybridisation between populations of these taxa, which accounts for the incongruent positions of these two hybrid individuals in the ITS and plastid phylogenies.

*Melanoseris* is not only the largest lineage of Lactucinae in China, but we have experienced it also taxonomically as particularly difficult. It comprises, on the one hand, elements that are morphologically so diverse that their affinities let alone relationships are far from obvious, on the other hand elements that constitute rather uniform groups in which the differences are predominantly gradual rather than clear-cut or of qualitative nature, and delimitations thus are often difficult to establish. Our molecular phylogenetic analyses provide the first indications that hybridisation and reticulate evolution could be one cause of this situation.

#### Notoseris Lineage

The genus *Notoseris* (for exemplar species see Fig. 3A–C) was established by Shih [22] to accommodate a number of tall forb species endemic to SW China, which share a combination of morphological features that set them apart from both the genera *Prenanthes* and *Nabalus*. These features were: nodding 3–5-flowered capitula; slender cylindrical involucres with purplish red phyllaries; purplish red flowers; purplish red, fusiform, unbeaked, somewhat compressed achenes (with 5 main ribs and 2 secondary ribs in between); pappus without an outer row of minute hairs. In his revised treatment, Shih [12] accepted 11 (plus two doubtful) species of *Notoseris*. Shih & Kilian [13] reduced this number to seven by changing several species concepts and transferring one species to *Paraprenanthes*. As a conclusion from our then initial ITS phylogeny, Shih & Kilian [13] added the two scandent species (Fig. 3B–C), formerly treated as *Prenanthes scandens* and *P. yakoensis* to *Notoseris,* which extended the original circumscription of the genus to capitula with up to 12 flowers and also non-purplish red but pale brown achenes. Their inclusion is corroborated by the present extended analysis. The scandent *Notoseris* species are not related to the scandent species of the subtribe in Africa and Indonesia, evolution of the scandent habit in subtribe Lactucinae thus has apparently occurred independently three times from montane tall forb ancestors (unpublished data).

Our phylogenetic analysis revealed that *Notoseris* in the revised sense of Shih & Kilian [13] is still not monophyletic. Two species, *N. melanantha* and *N. wilsonii*, are nested instead in the *Paraprenanthes* clade of the ITS phylogeny, or in the *Paraprenanthes* subclades of the joined *Notoseris-Paraprenanthes* clade of the plastid tree, and have thus to be excluded from *Notoseris* and transferred to the genus *Paraprenanthes* as *P. melanantha* and *P. wilsonii* (Fig. 3D–E; see Taxonomic conclusions). Possible causes of the topological incongruences were discussed above.

Additional evidences gathered in the present study from the taxonomic revision of all types and extensive studies of the species in the field, which support the molecular results, urge us to a further revision of the species concepts compared to both Shih [12] and Shih & Kilian [13]. It became obvious that discontinuities, of leaf features especially, inferred from the herbarium material of these tall forbs by Shih [12],[22] and used for the delimitation of species, frequently break down when variation is studied in the field. As herbarium specimen preservation of tall forbs (often exceeding 2 m in height) was in the past usually done highly selectively, even intraindividual variation of leaf shapes from the base to the top of the main axis was rarely documented to a sufficient extent, while leaf shape played an important role in the taxonomic treatments by Shih [12],[22]. Consequently, four other species compared to the last treatment by Shih & Kilian [13] are sunken in the synonymy here, leaving *Notoseris* with a total of six species only. So far known, all species are diploids with 2n = 18 [67]. The genus has its centre of diversity in SW China, where all six species occur. Four of them are endemic to China, the other two species also touch neighbouring countries.

#### Paraprenanthes Lineage

The genus *Paraprenanthes* (for exemplar species see Fig. 3D–F) was formally established by Shih [24], based on an earlier proposal by C. C. Chang, segregating species from *Lactuca* that are morphologically allied to *L. sororia*, which he designated as the type of the name *Paraprenanthes*. These species are usually tall forbs, they have usually nodding capitula with 3 (in the revised circumscription established here, 4 according to Shih [12])–15 cyanic flowers, slender cylindrical involucres, fusiform, somewhat compressed unbeaked dark brown to blackish achenes with 5 main ribs and 2 rather similar secondary ribs in between, and a pappus without an outer row of minute hairs. Formerly 11 species were distinguished by Shih [24], most of which newly described, the number increased to 15 finally [12]. Shih’s circumscription of the genus was maintained by Shih & Kilian [13], apart from the transfer of one species from *Notoseris* and the addition of a second one, following Sennikov [90], from *Mulgedium,* but somewhat wider species concepts were established, reducing the species number to 12.

Inferred from our analysis, the recent additions to the genus by Sennikov [90] and Shih & Kilian [13] are corroborated, but as hitherto circumscribed, *Paraprenanthes* is clearly paraphyletic. One group of species previously placed in *Notoseris* (*N. melanantha/wilsonii* group) and a second group of two species (*Cicerbita oligolepis/Stenoseris triflora* group) formerly placed in *Stenoseris* and *Cicerbita* [12],[25] or *Melanoseris* [13], respectively, must also be transferred to *Paraprenanthes* according to the evidence from both the nuclear and the chloroplast phylogeny. Although *Notoseris* and *Paraprenanthes* form a joined clade in the latter (see discussion, above), both groups clearly cluster with the respective *Paraprenanthes* subclades. The consequences for the morphological circumscription of the genus are, however, less significant, owing to the anyway shallow morphological divisions between the major lineages of the subtribe, and mainly concern the achenes, which can also be shortly beaked and pale brown. The case of putative introgressive hybridisation involving *Paraprenanthes melanantha* (as *Notoseris melanantha* in the trees), *P. wilsonii* (as *N. wilsonii* in the trees) and *P. sororia*, is discussed above.

Similar to the situation in *Notoseris,* the core of *Paraprenanthes* forms a polytomy in the ITS tree with the terminal taxa in most cases found individually on short or very short branches, reflecting the few character state differences in this marker sequence, whereas somewhat more resolution is provided by the plastid tree. The molecular evidence is in good accordance with the phenetic evidence, in so far as (a) speciation among the core of *Paraprenanthes* has not yet, in most cases, led to more conspicuous discontinuities, and (b) that even the wider species concepts applied by Shih & Kilian [13] compared to Shih [12] are still too narrow for quite similar reasons as stated for *Notoseris*. Supported by the taxonomic revision of all types, extensive studies of the species in the field, our revised taxonomy of *Paraprenanthes* halves the number of its species recognised by Shih & Kilian [13] to six. Adding the species newly to be transferred to this genus, we now recognise 10 species in *Paraprenanthes*, eight of which are endemic to China while two, *P. sororia* and *P. umbrosa,* extend to Vietnam and Japan, and Myanmar(?) and Thailand, respectively. So far known, all species are diploids with 2n = 18 [67]. The single exception of a chromosome count of 2n = 16 by Deng & al. [86] for *Stenoseris leptantha*, which is a synonym of *Paraprenanthes triflora,* vouchered by the specimen *Nie 1159* (KUN!), actually represents *Melanoseris tenuis*.

### Taxonomic Conclusions

Concluding from our molecular and morphological analyses, the latter also including the study of the type material of the names involved, we outline here a new classification of the genera *Notoseris*, *Paraprenanthes* and *Melanoseris* in their Chinese centre of diversity. It revises the recent classification of these genera by Shih & Kilian [13]. Full synonymies and further data are available through the Cichorieae Portal [27]. Monographic treatments of these genera are in preparation and will be the subject of consecutive publications.

#### 1. Notoseris

C. Shih in Acta Phytotax. Sin. 25: 196. 1987. – Type: *Notoseris psilolepis* C. Shih [ = *N. macilenta*].

6 species, all in China, 3 endemic ( = *).

Distribution: China (Chongqing, Guangdong, Guangxi, Guizhou, Hubei, Hunan, Jiangxi, Sichuan, Taiwan, Xizang, Yunnan) and E Himalaya region.

(1) ***Notoseris yakoensis*** (Jeffrey) N. Kilian in Wu & al., Fl. China 20–21: 231. 2011 ≡ *Prenanthes yakoensis* Jeffrey in Notes Roy. Bot. Gard. Edinburgh 5: 203. 1912.

 = *Prenanthes volubilis* Merr.

(2) ***Notoseris scandens*** (Hook. f.) N. Kilian in Wu & al., Fl. China 20–21: 231. 2011 ≡ *Prenanthes scandens* Hook. f. in Bentham & Hooker, Gen. Pl. 2: 527. 1873.

(*3) ***Notoseris triflora*** (Hemsl.) C. Shih in Acta Phytotax. Sin. 25: 202. 1987 ≡ *Lactuca triflora* Hemsl. in J. Linn. Soc., Bot. 23: 485. 1888.

(4) ***Notoseris khasiana*** (C. B. Clarke) N. Kilian in Wu & al., Fl. China 20–21: 233. 2011 ≡ *Prenanthes khasiana* C. B. Clarke, Comp. Ind.: 273. 1876.

 = *Notoseris rhombiformis* C. Shih, **syn. nov.**

(*5) ***Notoseris macilenta*** (Vaniot & H. Lév.) N. Kilian in Wu & al., Fl. China 20–21: 231. 2011 ≡ *Prenanthes macilenta* Vaniot & H. Lév. in Bull. Soc. Bot. France 53: 550. 1906.

 = *Notoseris psilolepis* C. Shih

 = *Notoseris formosana* (Kitam.) C. Shih

 = *Notoseris nanchuanensis* C. Shih, **syn. nov.**

 = *Notoseris guizhouensis* C. Shih, **syn. nov.**

 = *Notoseris yunnanensis* C. Shih, **syn. nov.**

(*6) ***Notoseris henryi*** (Dunn) C. Shih in Acta Phytotax. Sin. 25: 202. 1987 ≡ *Prenanthes henryi* Dunn in J. Linn. Soc., Bot. 35: 514. 1903.

 = *Notoseris porphyrolepis* C. Shih, **syn. nov.**

Excluded species:

*Notoseris melanantha* (Franch.) C. Shih in Acta Phytotax. Sin. 25: 198. 1987 ≡ ***Paraprenanthes melanantha*** (Franch.) Z. H. Wang

*Notoseris wilsonii* (C. C. Chang) C. Shih in Acta Phytotax. Sin. 25: 202. 1987 ≡ ***Paraprenanthes wilsonii*** **(**C. C. Chang) Z. H. Wang

#### 2. Paraprenanthes

C. C. Chang ex C. Shih in Acta Phytotax. Sin. 26: 418. 1988. – Type: *Paraprenanthes sororia* (Miq.) C. Shih.

 = *Lactuca* sect. *Prenanthesiae* Franch. in J. Bot. (Morot) 9: 291. 1895. – Lectotype (**here designated**): *Lactuca melanantha* Franch.

10 species, all in China, 8 endemic ( = *).

Distribution: China (Anhui, Chongqing, Fujian, Guangdong, Guangxi, Guizhou, Hainan, Hubei, Hunan, Jiangsu, Jiangxi, Shanxi, Sichuan, Taiwan, Xizang, Yunnan, Zhejiang), the E Himalayan region, Myanmar, Thailand, Vietnam and Japan.

(*1) ***Paraprenanthes oligolepis*** (C. C. Chang ex C. Shih) Z. H. Wang, **comb. nov.** ≡ *Cicerbita oligolepis* C. C. Chang ex C. Shih in Acta Phytotax. Sin. 29: 398. 1991 ≡ *Melanoseris oligolepis* (C. C. Chang ex C. Shih) N. Kilian, **syn. nov.**

(*2) ***Paraprenanthes triflora*** (C. C. Chang & C. Shih) Z. H. Wang & N. Kilian, **comb. nov.** ≡ *Stenoseris triflora* C. C. Chang & C. Shih in Acta Phytotax. Sin. 29: 413. 1991 ≡ *Melanoseris triflora* (C. C. Chang & C. Shih) N. Kilian, **syn. nov.**

 = *Stenoseris leptantha* C. Shih ≡ *Melanoseris leptantha* (C. Shih) N. Kilian, **syn. nov.**

(3) ***Paraprenanthes umbrosa*** (Dunn) Sennikov in Bot. Zhurn. 82(5): 111. 1997 ≡ *Lactuca umbrosa* Dunn in J. Linn. Soc., Bot. 35: 513. 1903 ≡ *Mulgedium umbrosum* (Dunn) C. Shih

 = ? *Lactuca parishii* Craib in Kew Bull. 1911: 403. 1911, **syn. nov.**

(4) ***Paraprenanthes sororia*** (Miq.) C. Shih in Acta Phytotax. Sin. 26: 422. 1988 ≡ *Lactuca sororia* Miq. in Ann. Mus. Bot. Lugduno-Batavi 2: 189. 1866

 = *Paraprenanthes pilipes* (Migo) C. Shih

 = *Paraprenanthes multiformis* C. Shih, **syn. nov.**

(*5) ***Paraprenanthes diversifolia*** (Vaniot) N. Kilian in Wu & al., Fl. China 20–21: 229. 2011 ≡ *Lactuca diversifolia* Vaniot in Bull. Acad. Int.Geogr. Bot. 12: 245. 1903

 = *Paraprenanthes sylvicola* C. Shih

 = *Paraprenanthes heptantha* C. Shih & D. J. Liou, **syn. nov.**

 = *Paraprenanthes gracilipes* C. Shih

(*6) ***Paraprenanthes yunnanensis*** (Franch.) C. Shih in Acta Phytotax. Sin. 26: 421. 1988 ≡ *Lactuca yunnanensis* Franch. in J. Bot. (Morot) 9: 264. 1895

 = *Paraprenanthes sagittiformis* C. Shih

 = *Paraprenanthes longiloba* Y. Ling & C. Shih, **syn. nov.**

 = *Paraprenanthes auriculiformis* C. Shih, **syn. nov.**

(*7) ***Paraprenanthes prenanthoides*** (Hemsl.) C. Shih in Acta Phytotax. Sin. 26: 423. 1988 ≡ *Crepis prenanthoides* Hemsl. in J. Linn. Soc., Bot. 23: 477. 1888

 = *Paraprenanthes glandulosissima* (C. C. Chang) C. Shih, **syn. nov.**

 = *Paraprenanthes polypodiifolia* (Franch.) C. Shih, **syn. nov.**

 = *Paraprenanthes thirionnii* (H. Lév.) C. Shih

 = *Paraprenanthes luchunensis* C. Shih**, syn. nov.**

(*8) ***Paraprenanthes meridionalis*** (C. Shih) Sennikov in Bot. Zhurn. 82(5): 111. 1997 ≡ *Mulgedium meridionale* C. Shihin Acta Phytotax. Sin. 26: 392. 1988

 = *Paraprenanthes hastata* C. Shih**, syn. nov.**

(*9) ***Paraprenanthes melanantha*** (Franch.) Z. H. Wang, **comb. nov.** ≡ *Lactuca melanantha* Franch. in J. Bot. (Morot) 9: 291. 1895 ≡ *Notoseris melanantha* (Franch.) C. Shih

Note: This species was misinterpreted by Shih & Kilian [13], where it was treated, under *Notoseris*, in a wide sense, merged with actually unrelated other species that have similar pinnately divided leaves. In the sense of its type, in contrast, it is a species endemic to Sichuan and Chongqing, well characterised by the combination of (a) a strikingly narrow, paniculiform, densely glandular synflorescence, (b) leaves with a large triangular to ovate or rhombic, basally cordate to cuneate terminal segment and 0–3(–6) pairs of lateral segments on a winged rachis, and (c) achenes with a ± truncate to attenuate apex.

(*10) ***Paraprenanthes wilsonii*** **(**C. C. Chang) Z. H. Wang, **comb. nov.** ≡ *Prenanthes wilsonii* C. C. Chang in Bull. Fan Mem. Inst. Biol., Bot. 5: 322. 1934 ≡ *Notoseris wilsonii* (C. C. Chang) C. Shih

 = *Notoseris gracilipes* C. Shih

 = *Paraprenanthes dolichophylla* (C. Shih) N. Kilian & Z. H. Wang in Wu & al., Fl. China 20–21: 229. 2011, **syn. nov.** ≡ *Notoseris dolichophylla* C. Shih

Note: *Paraprenanthes dolichophylla* is apparently very closely related to *P. wilsonii* and pending further assessmnent, is tentatively considered as conspecific here.

#### 3. Melanoseris

Decne. in Jacquemont, Voy. Inde 4: 101. 1843. – Lectotype (designated by Pfeiffer, Nomencl. Bot. 2: 259. 1874): *Melanoseris lessertiana* (DC.) Decne.

 = *Chaetoseris* C. Shih in Acta Phytotax. Sin. 29: 398. 1991. – Type: *Chaetoseris lyriformis* C. Shih [ = *Melanoseris cyanea* s.l.]

 = *Stenoseris* C. Shih in Acta Phytotax. Sin. 29: 411. 1991. – Type: *Stenoseris graciliflora* (DC.) C. Shih [≡ *Melanoseris graciliflora*]

 = *Parasyncalathium* J. W. Zhang & al. in Taxon 60: 1680. 2011. – Type: *Parasyncalathium souliei* (Franch.) J. W. Zhang & al. [≡ *Melanoseris souliei*]

Some 70 species in total, 17 species in China, 9 endemic ( = *).

Distribution: China (Chongqing, Guizhou, Sichuan, Xizang, Yunnan); Himalayas and adjacent areas, SW and Central Asia, sub-Saharian Africa.

Notes: In the Himalayan territories the following seven species of *Melanoseris* are distributed but not known to occur in China: *M. brunoniana* (Wall. ex DC.) N. Kilian & Z. H. Wang, **comb. nov.** ≡ *Prenanthes brunoniana* Wall. ex DC., Prodr. 7(1): 195. 1838; *M. decipiens* (Hook. f. & Thomson ex C. B. Clarke) N. Kilian & Z. H. Wang, **comb. nov.** ≡ *Lactuca decipiens* Hook. f. & Thomson ex C. B. Clarke, Compos. Ind.: 266. 1876; *M. filicina* (Stebbins) N. Kilian, **comb. nov.** ≡ *Lactuca filicina* Duthie ex Stebbins in Indian Forest Rec., Bot. 1: 241. 1939; *M. kashmiriana* (Mamgain & R. R. Rao) N. Kilian, **comb. nov.** ≡ *Lactuca kashmiriana* Mamgain & R. R. Rao in J. Bombay Nat. Hist. Soc. 83: 406–408. 1986; *M. lahulensis* (Mamgain & R. R. Rao) N. Kilian, **comb. nov.** ≡ *Lactuca lahulensis* Mamgain & R. R. Rao in Bull. Bot. Surv. India 27: 120–122. 1987; *M. polyclada* (Boiss.) Akhani, N. Kilian & Sennikov, **comb. nov.** ≡ *Zollikoferia polyclada* Boiss., Fl. Orient. 3: 827. 1875; *M. rapunculoides* (DC.) Edgeworth.

(1) ***Melanoseris bracteata*** (C. B. Clarke) N. Kilian in Wu & al., Fl. China 20–21: 225. 2011 ≡ *Lactuca bracteata* C. B. Clarke, Compos. Ind.: 270. 1876 ≡ *Mulgedium bracteatum* (C. B. Clarke) C. Shih

(*2) ***Melanoseris souliei*** (Franch.) N. Kilian in Wu & al., Fl. China 20–21: 225. 2011 ≡ *Lactuca souliei* Franch. in J. Bot. (Morot) 9: 257. 1895 ≡ *Syncalathium souliei* (Franch.) Y. Ling ≡ *Parasyncalathium souliei* (Franch.) J. W. Zhang & al.

 = *Syncalathium orbiculariforme* C. Shih

(3) ***Melanoseris*** ** ***qinghaica*** (S. W. Liu & T. N. Ho) N. Kilian & Z. H. Wang, **comb. nov.** ≡ *Mulgedium qinghaicum* S. W. Liu & T. N. Ho in Acta Phytotax. Sin. 39: 556. 2001

Note: Tentatively included by Shih & Kilian [13] in a rather widely circumscribed *Melanoseris lessertiana*, our analyses since have revealed that all reports of *M. lessertiana* from China are actually referable to *M. qinghaica,* which is mainly distinguished by the distinctly longer achene beak and a very short anthertube. *M. lessertiana* is restricted to the Himalayas.

(4) ***Melanoseris cyanea*** (D. Don) Edgew. in Trans. Linn. Soc. London 20: 81. 1846 ≡ *Sonchus cyaneus* D. Don, Prodr. Fl. Nepal. 164. 1825 ≡ *Chaetoseris cyanea* (D. Don) C. Shih

 = *Melanoseris beesiana* (Diels) N. Kilian, **syn. nov.** ≡ *Chaetoseris beesiana* (Diels) C. Shih

 = *Chaetoseris hastata* (DC.) C. Shih ≡ *Melanoseris hastata* (DC.) Edgew.

 = *Chaetoseris hispida* C. Shih

 = *Chaetoseris lyriformis* C. Shih

 = *Melanoseris sichuanensis* (C. Shih) N. Kilian, **syn. nov.** ≡ *Chaetoseris sichuanensis* C. Shih

Tentatively included:

*Chaetoseris lutea* (Hand.-Mazz.) C. Shih in Acta Phytotax. Sin. 29: 409. 1991 ≡ *Cicerbita cyanea* var. *lutea* Hand.-Mazz., Symb. Sin. 7: 1180. 1936.

*Melanoseris yunnanensis* (C. Shih) N. Kilian & Z. H. Wang in Wu & al., Fl. China 20–21: 219. 2011 ≡ *Chaetoseris yunnanensis* C. Shih [ = *Chaetoseris teniana* (Beauverd) C. Shih ≡ *Cicerbita cyanea* var. *teniana* Beauverd]

*Melanoseris pectiniformis* (C. Shih) N. Kilian & J. W. Zhang in Wu & al., Fl. China 20–21: 222. 2011 ≡ *Chaetoseris pectiniformis* C. Shih

Note: *Melanoseris cyanea* is a widespread species and polymorphic especially with respect to indumentum features. The wider concept of the species, compared to Shih [12], used by Shih & Kilian [13] is still too narrow: (1) The delimitation towards *M. beesiana* ( = *Chaetoseris lyriformis* C. Shih) as well as towards *M. sichuanensis* breaks, when considering besides leaf shape features also relevant capitula and flower features. (2) The status of the yellow-flowered plants and populations treated by Shih & Kilian [13] under *Melanoseris yunnanensis* ( = *Chaetoseris lutea = C. teniana*) is still not fully clear, their very close relationship to *M. cyanea* is proven, however, by the molecular analysis. (3) The status and assignment of *M. pectiniformis* are still not beyond doubt.

(*5) ***Melanoseris ciliata*** (C. Shih) N. Kilian in Wu & al., Fl. China 20–21: 219. 2011 ≡ *Chaetoseris ciliata* C. Shih in Acta Phytotax. Sin. 29: 403. 1991

(*6) ***Melanoseris macrocephala*** (C. Shih) N. Kilian & J. W. Zhang in Wu & al., Fl. China 20–21: 221. 2011 ≡ *Chaetoseris macrocephala* C. Shih in Acta Phytotax. Sin. 29: 404. 1991

(7) ***Melanoseris macrorhiza*** (Royle) N. Kilian in Wu & al., Fl. China 20–21: 224. 2011 ≡ *Mulgedium macrorhizum* Royle, Ill. Bot. Himal. Mts. 1: 251. 1835 ≡ *Cephalorrhynchus macrorhizus* (Royle) Tuisl

 = *Cephalorrhynchus albiflorus* C. Shih

(8) ***Melanoseris violifolia*** (Decne.) N. Kilian in Wu & al., Fl. China 20–21: 225. 2011 ≡ *Prenanthes violifolia* Decne. in Jacquemont, Voy. Inde 4. 1843

 = *Cicerbita sikkimensis* (Hook. f.) C. Shih

(9) ***Melanoseris macrantha*** (C. B. Clarke) N. Kilian & J. W. Zhang in Wu & al., Fl. China 20–21: 219. 2011 ≡ *Lactuca macrantha* C. B. Clarke, Compos. Ind.: 267. 1876 ≡ *Chaetoseris macrantha* (C. B. Clarke) C. Shih

(*10) ***Melanoseris likiangensis*** (Franch.) N. Kilian & Z. H. Wang in Wu & al., Fl. China 20–21: 222. 2011 ≡ *Lactuca likiangensis* Franch. in J. Bot. (Morot) 9: 259. 1895 ≡ *Chaetoseris likiangensis* (Franch.) C. Shih

(*11) ***Melanoseris bonatii*** (Beauverd) Z. H. Wang, **comb. nov.** ≡ *Cicerbita bonatii* Beauverd in Bull. Soc. Bot. Genève 2: 126. 1910 ≡ *Chaetoseris bonatii* (Beauverd) C. Shih

(12) ***Melanoseris atropurpurea*** (Franch.) N. Kilian & Z. H. Wang in Wu & al., Fl. China 20–21: 221. 2011 ≡ *Lactuca atropurpurea* Franch. in J. Bot. (Morot) 9: 260. 1895 ≡ *Chaetoseris grandiflora* (Franch.) C. Shih, nom. illeg.

 = *Melanoseris taliensis* (C. Shih) N. Kilian & Z. H. Wang, **syn. nov.** ≡ *Chaetoseris taliensis* C. Shih

(*13) ***Melanoseris leiolepis*** (C. Shih) N. Kilian & J. W. Zhang in Wu & al., Fl. China 20–21: 222. 2011 ≡ *Chaetoseris leiolepis* C. Shih in Acta Phytotax. Sin. 29: 402. 1991

(*14) ***Melanoseris dolichophylla*** (C. Shih) Z. H. Wang, **comb. nov.** ≡ *Chaetoseris dolichophylla* C. Shih in Acta Phytotax. Sin. 29: 401. 1991

Note: Included in the synonymy of *Melanoseris atropurpurea* by Shih & Kilian [13], herbarium work by the first author revealed it to be a separate species, consistently distinguished by the absence of a main stem, long rosette leaves and subscapose stems with 1–2 capitula only.

(*15) ***Melanoseris monocephala*** (C. C. Chang) Z. H. Wang, **comb. nov.** ≡ *Lactuca monocephala* C. C. Chang in Contr. Biol. Lab. Sci. Soc. China, Bot. Ser. 9: 132. 1934 ≡ *Mulgedium monocephalum* (C. C. Chang) C. Shih

Note: This fairly rare species was, with doubts, considered by Shih & Kilian [13] as conspecific with *Melanoseris lessertiana,* it is, however, unrelated and clearly distinct.

(16) ***Melanoseris graciliflora*** (DC.) N. Kilian in Wu & al., Fl. China 20–21: 223. 2011 ≡ *Lactuca graciliflora* DC., Prodr. 7: 139. 1838 ≡ *Stenoseris graciliflora* (DC.) C. Shih

 = *Stenoseris taliensis* (Franch.) C. Shih

(*17) ***Melanoseris tenuis*** (C. Shih) N. Kilian in Wu & al., Fl. China 20–21: 223. 2011 ≡ *Stenoseris tenuis* C. Shih in Acta Phytotax. Sin. 29: 412. 1991

Excluded species:

*Melanoseris oligolepis* (C. C. Chang ex C. Shih) N. Kilian in Wu & al., Fl. China 20–21: 224. 2011 ≡ ***Paraprenanthes oligolepis*** (C. C. Chang ex C. Shih) Z. H. Wang

*Melanoseris triflora* (C. C. Chang & C. Shih) N. Kilian in Wu & al., Fl. China 20–21: 223. 2011 ≡ ***Paraprenanthes triflora*** (Chang & C. Shih) Z. H. Wang & N. Kilian

Note: See under *Paraprenanthes.*

*Melanoseris rhombiformis* (C. Shih) N. Kilian & Z. H. Wang in Wu & al., Fl. China 20–21: 219. 2011 ≡ *Chaetoseris rhombiformis* C. Shih. = ***“Cicerbita” roborowskii***

Note: Analysis of the type of the name *Chaetoseris rhombiformis* by the first author made it evident that this yellow-flowered species is actually referable to *“Cicerbita” roborowskii.* This appears surprising because the latter species has always been considered to be blue-flowered (with occasional white forms). However, yellow-flowered individuals that are clearly conspecific with *C. roborowskii,* as inferred from both morphological and molecular analysis, have been collected from Sichuan (*Kilian & al. 10809* at B, KUN; see also images in [27] under that species).

#### 4. Species Of Uncertain Status And Placement

*Melanoseris hirsuta* (C. Shih) N. Kilian in Wu & al., Fl. China 20–21: 220. 2011 ≡ *Chaetoseris hirsuta* C. Shih ≡ *Lactuca hirsuta* Franch. 1895 [non Nutt. 1818]

*Melanoseris henryi* (Dunn) N. Kilian in Wu & al., Fl. China 20–21: 221. 2011 ≡ *Lactuca henryi* Dunn

*Lactuca scandens* C. C. Chang in Contr. Biol. Lab. Sci. Soc. China, Bot. Ser. 9: 133. 1934

## Supporting Information

Appendix S1 **Plant material used.** The data are arranged in the following order: accepted taxon name in bold and synonyms used in the phylograms (Fig. 1–2) in square brackets; unique sample identifier also used in the phylograms and, in square brackets where applicable, unit ID in the GGBN data portal [91] of stored DNA sample; abbreviated voucher data (country, locality, collecting date, collectors and collecting number, herbarium code according to Thiers [26]), full data can be obtained from the specimen labels; EMBL/Genbank/DDBJ accession numbers in the following sequence: ITS, *petD, psbA-trnH, 5′trnL^(UAA)^-trnF, rpl32-trnL^(UAG)^, trnQ^(UUG)^-5′rps16.* In the few cases, where already published sequences were used, only the EMBL/Genbank/DDBJ accession number preceded by an asterisk is given.
(PDF)

Appendix S2 **Positions of mutational hotspots ( = HS) and exons in the individual chloroplast marker sequences excluded from phylogenetic analysis.** The position within each marker sequence is calculated without gap; a dash denotes the absence of this sequence portion in the corresponding samples.
(PDF)

Appendix S3 **Indels coded in the phylogenetic analysis.** For each marker, position, length [nt] and description of the coded indels are given according to the sequences alignment matrix.
(PDF)
