# Supplementary material for: Molecular Phylogeny of the Lactuca Alliance (Cichorieae Subtribe Lactucinae, Asteraceae) with Focus on Their Chinese Centre of Diversity Detects Potential Events of Reticulation and Chloroplast Capture
Source: PLoS One. 2013 Dec 20;8(12):e82692. doi: 10.1371/journal.pone.0082692 (PMC3871690; doi:10.1371/journal.pone.0082692)
Supplement: Appendix S1 — Plant material used. The data are arranged in the following order: accepted taxon name in bold and synonyms used in the phylograms (Fig. 1–2) in square brackets; unique sample identifier also used in the phylograms and, in square brackets where applicable, unit ID in the GGBN data portal [91] of stored DNA sample; abbreviated voucher data (country, locality, collecting date, collectors and collecting number, herbarium code according to Thiers [26]), full data can be obtained from the specimen labels; EMBL/Genbank/DDBJ accession numbers in the following sequence: ITS, petD, psbA-trnH, 5′trnL(UAA)-trnF, rpl32-trnL(UAG), trnQ(UUG)-5′rps16. In the few cases, where already published sequences were used, only the EMBL/Genbank/DDBJ accession number preceded by an asterisk is given. (PDF) [file pone.0082692.s001.pdf]

Appendix S1. Plant material used. The data are arranged in the following order: accepted taxon name in bold and synonyms used in the phylograms (Fig. 1–2) in square brackets; unique sample identifier also used in the phylograms and, in square brackets where applicable, unit ID in the GGBN data portal [91] of stored DNA sample; abbreviated voucher data (country, locality, collecting date, collectors and collecting number, herbarium code according to Thiers [26]), full data can be obtained from the specimen labels; EMBL/Genbank/ DDBJ accession numbers in the following sequence: ITS, *petD, psbA-trnH, 5'trnL$^{(UAA)}$-trnF, rpl32-trnL$^{(UAG)}$, trnQ$^{(UUG)}$-5'rps16.* In the few cases, where already published sequences were used, only the EMBL/Genbank/DDBJ accession number preceded by an asterisk is given.

Outgroups: ***Crepis multicaulis*** Ledeb.: LAC-003: Russia, S Siberia, Altay Mts, 22 Jun 2002, *E. von Raab-Straube 020302* (B), KF485539, KF485665, KF485794, KF486050, KF485922, KF486178. — ***Launaea sarmentosa*** (Willd.) Kuntze: LAC-001: Sri Lanka, S coast, Dikwella, 25 Dec 2000, *N. Kilian 7001* (B), KF485537, KF485663, KF485792, KF486048, KF485920, KF486176. — ***Leontodon tuberosus*** L.: LAC-002 [DB4947]: Greece, Insel Rhodos, Salakos, 23 Mar 2009, *D. Lauterbach, T. Böhmer & A. Rolf RH2-182* (B), KF485538, KF485664, KF485793, KF486049, KF485921, KF486177. — ***Soroseris erysimoides*** (Hand.-Mazz.) C. Shih: LAC-004: China, Sichuan, Ganzi, 2 Aug 2009, *Y. S. Chen & Z. H. Wang 9270* (KUN), KF485540, KF485666, KF485795, KF486051, KF485923, KF486179.

Faberia faberi (Hemsley) N. Kilian [= *Prenanthes faberi* Hemsley]: LAC-008: China, Chongqing, Nanchuan, 18 Sep 2010, *Z. H. Wang & L. Chen 245* (KUN), KF485544, KF485670, KF485799, KF486055, KF485927, KF486183; LAC-009: China, Yunnan, Qiaojia, 21 Jul 2009, *Y. S. Chen & Z. H. Wang 9034* (KUN), KF485545, KF485671, KF485800, KF486056, KF485928, KF486184; LAC-010: China, Guizhou, Hezhang, 1 Sep 2012, *Z. H. Wang & L. Chen 485* (KUN), KF485546, KF485672, KF485801, KF486057, KF485929, KF486185. ***F. nanchuanensis*** C. Shih [= *Faberiopsis nanchuanensis* (C. Shih) C. Shih & Y. L. Chen]: LAC-005: China, Chongqing, Nanchuan, 14 Jul 2010, *Z. H. Wang & L. Chen 131* (KUN), KF485541, KF485667, KF485796, KF486052, KF485924, KF486180. ***F. sinensis*** Hemsley: LAC-006 [DB3264]: China, Sichuan, Emei, 6 Jul 1997, *C. H. Li 590* (MO 04513521), KF485542, KF485668, KF485797, KF486053, KF485925, KF486181; LAC-007: China, Sichuan, Emei, 8 Jul 2010, *Z. H. Wang & L. Chen 63* (KUN), KF485543, KF485669, KF485798, KF486054, KF485926, KF486182. — ***Prenanthes purpurea*** L.: LAC-013: cult. Bot. Garten Berlin-Dahlem, Acc. 211-48-74-80, 19 Aug 2010, *M. Cubr 47381* (B), KF485548, KF485675, KF485804, KF486059, KF485931, KF486187.

Lactucinae **core group:** ***Cicerbita alpina*** (L.) Wallr.: LAC-012: *AJ633340; LAC-133 [DB7934]: Norway, Hordaland, Hardangarvidda, 15 Aug 2008, *T. Dürbye 4350* (B), —, KF485674, KF485803, KF486058, KF485930, KF486186. ***C. hispida*** (DC.) Beauverd [= *Cephalorrhynchus hispidus* (DC.) Boiss.]: LAC-011 [DB0050]: Armenia, Vayotsdzor, Vajk, 24 Jun 2002, *C. Oberprieler 10145* (B), KF485547, KF485673, KF485802, —, —, —. — ***Cicerbita*** **II:** ***C. auriculiformis*** (C. Shih) N. Kilian [= *Stenoseris auriculiformis* C. Shih]: LAC-

016: China, Qinghai, Huzhu, 7 Jun 1991, *T. N. Ho 1730* (CAS 934678), KF485551, KF485678, —, KF486062, KF485934, KF486190; LAC-017: China, Gansu, Yongdeng, 5 Aug 2006, *Z. G. Sun & al. 06367* (PE), KF485552, KF485679, KF485807, KF486063, KF485935, KF486191. ***C. azurea*** (Ledeb.) Beauverd: LAC-014: Russia, S Siberia, Altay Mts, 25 Jul 2002, *E. von Raab-Straube 020364* (B), KF485549, KF485676, KF485805, KF486060, KF485932, KF486188; LAC-015: Russia, S Siberia, Altay Mts, 27 Jul 2002, *E. von Raab-Straube 020408* (B), KF485550, KF485677, KF485806, KF486061, KF485933, KF486189. ***C. roborowskii*** (Maxim.) Beauverd [= *Chaetoseris roborowskii* (Maxim.) C. Shih]: LAC-018: China, Sichuan, Xiaojin, 29 Jul 2009, *Y. S. Chen & Z. H. Wang 9184* (KUN), KF485553, KF485680, KF485808, KF486064, KF485936, KF486192; LAC-019: China, Sichuan, Xiangcheng, 12 Aug 2010, *E. D. Liu & al. 2688* (KUN), KF485554, KF485681, KF485809, KF486065, KF485937, KF486193. — ***Lactuca dissecta*** D. Don: LAC-116: Tadshikistan, Warob gorge, 30 Jul 1991, *N. Kilian 2547* (B), KF485649, KF485777, KF485905, KF486161, KF486033, KF486289. ***L. dolichophylla*** Kitam.: LAC-117: China, Yunnan, Deqin, 20 Sep 2011, *H. J. Dong & al. 615* (KUN), KF485650, KF485778, KF485906, KF486162, KF486034, KF486290. ***L. formosana*** Maxim. [= *Pterocypsela formosana* (Maxim.) C. Shih, *P. sonchus* (H. Lév. & Vaniot) C. Shih]: LAC-122: China, Shanxi, Yongji, 20 Aug 2009, *H. Peng & al. 417* (KUN), KF485655, KF485783, KF485911, KF486167, KF486039, KF486295; LAC-123: China, Chongqing, Nanchuan, 26 Aug 2009, *H. Peng & al. 603* (KUN), KF485656, KF485784, KF485912, KF486168, KF486040, KF486296. ***L. indica*** L. [= *Pterocypsela indica* L., *P. laciniata* (Houtt.) C. Shih]: LAC-120: China, Chongqing, Nanchuan, 17 Sep 2010, *Z. H. Wang & L. Chen 241* (KUN), KF485653, KF485781, KF485909, KF486165, KF486037, KF486293; LAC-121: China, Shanxi, Yuanqu, 20 Aug 2009, *H. Peng & al. 500* (KUN), KF485654, KF485782, KF485910, KF486166, KF486038, KF486294. ***L. inermis*** Forssk.: LAC-119: Togo, 1980, *J. F. Brunel 7281* (B), KF485652, KF485780, KF485908, KF486164, KF486036, KF486292. ***L. orientalis*** (Boiss.) Boiss. [= *Scariola orientalis* (Boiss.) Soják]: LAC-126: Iran, Isphahan, 30 Sep 1998, *Weber* (B), KF485659, KF485787, KF485915, KF486171, KF486043, KF486299. ***L. perennis*** L.: LAC-113: *AJ633334; LAC-334 [DB5188]: Italy, Piemont. Cuneo, 12 Jul 2009, *M. Ristow & al. MiRi 578/09* (B), —, KF485774, KF485902, KF486158, KF486030, KF486286. ***L. raddeana*** Maxim. [= *Pterocypsela raddeana* (Maxim.) C. Shih, *P. elata* (Hemsl.) C. Shih]: LAC-125: China, Sichuan, Dayi, 25 Jul 2009, *Y. S. Chen & Z. H. Wang 9101* (KUN), KF485658, KF485786, KF485914, KF486170, KF486042, KF486298; LAC-124: China, Sichuan, Luding, 6 Aug 2010, *E. D. Liu & al. 2608* (KUN), KF485657, KF485785, KF485913, KF486169, KF486041, KF486297. ***L. sativa*** L.: LAC-130: *AJ633337; LAC-132: cp markers *DQ383816. ***L. serriola*** L.: LAC-131: China, Yunnan, Kunming, 1 May 2011, *Z. H. Wang & L. Chen 487* (KUN), KF485662, KF485791, KF485919, KF486175, KF486047, KF486303. ***L. sibirica*** (L.) Maxim. [= *Lagedium sibiricum* (L.) Soják]: LAC-128: Russia, Siberia, 2004, *M. Ristow 991-04* (herb. Ristow), KF485660, KF485789, KF485917, KF486173, KF486045, KF486301. ***L. tatarica*** (L.) C. A. Mey. [= *Mulgedium tataricum* (L.) DC.]: LAC-129: China, Hebei, 2009, *H. Peng & al. 584* (KUN), KF485661, KF485790, KF485918, KF486174,

KF486046, KF486302. ***L. tuberosa*** Jacq. [= *Steptorhamphus tuberosus* (Jacq.) Grossh.]: LAC-118: Cyprus, Kannavious, 5 May 1998, *R. Hand 2410* (herb. Hand), KF485651, KF485779, KF485907, KF486163, KF486035, KF486291. ***L. undulata*** Ledeb.: LAC-114: Iran, Kavir, 1974, *K. H. Rechinger 46203* (B), KF485647, KF485775, KF485903, KF486159, KF486031, KF486287; LAC-115: China, Xinjiang, Shawan, 11 Jul 1957, *S. Xin 509* (KUN), KF485648, KF485776, KF485904, KF486160, KF486032, KF486288. ***L. viminea*** (L.) J. Presl & C. Presl [= *Scariola viminea* (L.) F. W. Schmidt]: LAC-127: *AJ633333; LAC-135 [DB0126]: Italy, Marche, Camerino, 29 Sep 1999, *A. Ueckert & al. CHO 10025* (B), —, KF485788, KF485916, KF486172, KF486044, KF486300. — ***Melanoseris atropurpurea*** (Franch.) N. Kilian & Z. H. Wang [= *Chaetoseris grandiflora* (Franch.) C. Shih, *C. taliensis* C. Shih]: LAC-099: China, Xizang, Milin, 11 Aug 2009, *Y. S. Chen & Z. H. Wang 9402* (KUN), KF485633, KF485760, KF485888, KF486144, KF486016, KF486272; LAC-100: China, Yunnan, Dali, 17 Oct 2010, *Z. J. Yin & al. 3267* (KUN), KF485634, KF485761, KF485889, KF486145, KF486017, KF486273; LAC-101: China, Yunnan, Dali, 10 Sep 2009, *Z. J. Yin & al. 1971* (KUN), KF485635, KF485762, KF485890, KF486146, KF486018, KF486274. ***M. bracteata*** (C. B. Clarke) N. Kilian [= *Mulgedium bracteatum* (C. B. Clarke) C. Shih]: LAC-072: China, Xizang, Cuona, 7 Sep 2012, *G. X. Hu & al. 1209024* (KUN), KF485607, KF485734, KF485862, KF486118, KF485990, KF486246. ***M. cyanea*** (D. Don) Edgew. [= *Chaetoseris cyanea* (D. Don) C. Shih, *C. hastata* (DC.) C. Shih, *C. hispida* C. Shih, *C. lutea* (Hand.-Mazz.) C. Shih, *C. lyriformis* C. Shih, *C. sichuanensis* C. Shih, *C. yunnanensis* C. Shih]: LAC-083: China, Xizang, Chayu, 17 Sep 2012, *G. X. Hu & al. 1209075* (KUN), KF485617, KF485744, KF485872, KF486128, KF486000, KF486256; LAC-089: China, Yunnan, Dali, 17 Sep 2009, *Z. J. Yin & al. 2362* (KUN), KF485623, KF485750, KF485878, KF486134, KF486006, KF486262; LAC-080: China, Yunnan, Dali, 11 Sep 2009, *Z. J. Yin & al. 2135* (KUN), KF485614, KF485741, KF485869, KF486125, KF485997, KF486253; LAC-090: China, Yunnan, Eryuan, 22 Sep 2011, *H. J. Dong & al. 640* (KUN), KF485624, KF485751, KF485879, KF486135, KF486007, KF486263; LAC-084: China, Yunnan, Dali, 13 Sep 2009, *Z. J. Yin & al. 2214* (KUN), KF485618, KF485745, KF485873, KF486129, KF486001, KF486257; LAC-085: China, Yunnan, Eryuan, 22 Sep 2011, *H. J. Dong & al. 632* (KUN), KF485619, KF485746, KF485874, KF486130, KF486002, KF486258; LAC-087: China, Yunnan, 2009, *J. Y. Xiang 09-112* (KUN), KF485621, KF485748, KF485876, KF486132, KF486004, KF486260; LAC-088: China, Yunnan, Dali, 17 Sep 2009, *Z. J. Yin & al. 2394* (KUN), KF485622, KF485749, KF485877, KF486133, KF486005, KF486261; LAC-091: China, Yunnan, Eryuan, 22 Sep 2011, *H. J. Dong & al. 634* (KUN), KF485625, KF485752, KF485880, KF486136, KF486008, KF486264; LAC-086: China, Yunnan, Lijiang, 17 Sep 2011, *H. J. Dong & al. 494* (KUN), KF485620, KF485747, KF485875, KF486131, KF486003, KF486259; LAC-092: China, Yunnan, Lijiang, 1 Sep 2012, *H. Peng & al. 2012-368* (KUN), KF485626, KF485753, KF485881, KF486137, KF486009, KF486265; LAC-081: China, Yunnan, Lanping, 3 Oct 2009, *H. Peng & al. 715* (KUN), KF485615, KF485742, KF485870, KF486126, KF485998, KF486254; LAC-082: China, Xizang, Bomi, 21 Sep 2012, *G. X. Hu & al. 1209094* (KUN), KF485616, KF485743, KF485871,

KF486127, KF485999, KF486255. ***M. cyanea* hybrid** [= *Chaetoseris cyanea* hybrid]: LAC-093: China, Yunnan, Eryuan, 22 Sep 2011, *H. J. Dong & al. 643* (KUN), KF485627, KF485754, KF485882, KF486138, KF486010, KF486266; LAC-094: China, Yunnan, Dali, 7 Nov 2012, *Z. H. Wang & L. Chen 479* (KUN), KF485628, KF485755, KF485883, KF486139, KF486011, KF486267; LAC-095: China, Yunnan, Dali, 7 Nov 2012, *Z. H. Wang & L. Chen 481* (KUN), KF485629, KF485756, KF485884, KF486140, KF486012, KF486268; LAC-096: China, Yunnan, Dali, 7 Nov 2012, *Z. H. Wang & L. Chen 480_1* (KUN), KF485630, KF485757, KF485885, KF486141, KF486013, KF486269; LAC-097: China, Yunnan, Dali, 7 Nov 2012, *Z. H. Wang & L. Chen 480_2* (KUN), KF485631, KF485758, KF485886, KF486142, KF486014, KF486270. ***M. graciliflora*** (DC.) N. Kilian [= *Stenoseris graciliflora* (DC.) C. Shih, *S. taliensis* (Franch.) C. Shih]: LAC-110: China, Sichuan, Tianquan, 8 Sep 2010, *Z. H. Wang & L. Chen 159* (KUN), KF485644, KF485771, KF485899, KF486155, KF486027, KF486283; LAC-112: China, Sichuan, Luding, 8 Sep 2010, *Z. H. Wang & L. Chen 188* (KUN), KF485646, KF485773, KF485901, KF486157, KF486029, KF486285; LAC-111: China, Sichuan, Luding, 8 Sep 2010, *Z. H. Wang & L. Chen 186* (KUN), KF485645, KF485772, KF485900, KF486156, KF486028, KF486284. ***M. lessertiana*** (DC.) Decne. [= *Mulgedium lessertianum* DC.]: LAC-071: Pakistan, Nanga Parbat, 28 Jul 1993, *M. Nüsser 157* (B), KF485606, KF485733, KF485861, KF486117, KF485989, KF486245. ***M. likiangensis*** (Franch.) N. Kilian & Z. H. Wang [= *Chaetoseris likiangensis* (Franch.) C. Shih]: LAC-098: China, Yunnan, Lijiang, 1 Sep 2012, *H. Peng & al. 2012-396* (KUN), KF485632, KF485759, KF485887, KF486143, KF486015, KF486271. ***M. macrantha*** (C. B. Clarke) N. Kilian & J. W. Zhang [= *Chaetoseris macrantha* (C. B. Clarke) C. Shih]: LAC-075: Bhutan, 4200 m, 2000, *S. Miehe & G. Miehe 00-223-12* (herb. Miehe), KF485610, KF485737, KF485865, KF486121, KF485993, KF486249. ***M. macrorhiza*** (Royle) N. Kilian [= *Cephalorrhynchus macrorhizus* (Royle) Tuisl]: LAC-073: Nepal, Mustang, 2001, *S. Miehe, G. Miehe & K. Koch 01-120-03* (herb. Miehe), KF485608, KF485735, KF485863, KF486119, KF485991, KF486247; LAC-074: China, Xizang, Milin, 22 Sep 2012, *G. X. Hu & al. 1209101* (KUN), KF485609, KF485736, KF485864, KF486120, KF485992, KF486248. ***M. qinghaica*** (S. W. Liu & T. N. Ho) N. Kilian & Z. H. Wang [= *Mulgedium qinghaicum* S. W. Liu & T. N. Ho]: LAC-078: Bhutan, 4200-4300 m, 2001, *S. Miehe & G. Miehe 00-346-01* (herb. Miehe), KF485613, KF485740, KF485868, KF486124, KF485996, KF486252. ***M. souliei*** (Franch.) N. Kilian [= *Parasyncalathium souliei* (Franch.) J. W. Zhang & al.]: LAC-069: China, Xizang, Bomi, 7 Aug 2009, *Y. S. Chen & Z. H. Wang 9315* (KUN), KF485604, KF485731, KF485859, KF486115, KF485987, KF486243; LAC-070: China, Sichuan, Dege, 3 Aug 2009, *Y. S. Chen & Z. H. Wang 9292* (KUN), KF485605, KF485732, KF485860, KF486116, KF485988, KF486244. ***M.* sp.** [= *Chaetoseris* sp.]: LAC-103: China, Yunnan, Zhanyi, 7 Nov 2012, *Z. H. Wang & L. Chen 486* (KUN), KF485637, KF485764, KF485892, KF486148, KF486020, KF486276. ***M. tenuis*** (C. Shih) N. Kilian [= *Stenoseris tenuis* C. Shih]: LAC-104: China, Yunnan, Dali, 7 Nov 2012, *Z. H. Wang & L. Chen 482_2* (KUN), KF485638, KF485765, KF485893, KF486149, KF486021, KF486277; LAC-105: China, Yunnan, Dali, 7 Nov 2012, *Z. H. Wang & L. Chen 482_1* (KUN), KF485639, KF485766,

KF485894, KF486150, KF486022, KF486278; LAC-106: China, Yunnan, Dali, 17 Oct 2010, *Z. J. Yin & al. 3275* (KUN), KF485640, KF485767, KF485895, KF486151, KF486023, KF486279; LAC-107: China, Yunnan, Dali, 10 Sep 2009, *Z. J. Yin & al. 1969* (KUN), KF485641, KF485768, KF485896, KF486152, KF486024, KF486280. ***M. tenuis* hybrid** [= *Stenoseris tenuis* hybrid]: LAC-102: China, Yunnan, Dali, 17 Oct 2010, *Z. J. Yin & al. 3277* (KUN), KF485636, KF485763, KF485891, KF486147, KF486019, KF486275; LAC-108: China, Yunnan, Dali, 7 Nov 2012, *Z. H. Wang & L. Chen 476_1* (KUN), KF485642, KF485769, KF485897, KF486153, KF486025, KF486281; LAC-109: China, Yunnan, Dali,7 Nov 2012, *Z. H. Wang & L. Chen 476_3* (KUN), KF485643, KF485770, KF485898, KF486154, KF486026, KF486282. ***M. violifolia*** (Decne.) N. Kilian [= *Cicerbita sikkimensis* (Hook. f.) C. Shih]: LAC-076: China, Xizang, Cuona, Mama, 19 Sep 2009, *Ze-Long Nie 806* (KUN), KF485611, KF485738, KF485866, KF486122, KF485994, KF486250; LAC-077: China, Xizang, Cuona, 7 Sep 2012, *G. X. Hu & al. 1209027* (KUN), KF485612, KF485739, KF485867, KF486123, KF485995, KF486251. — ***Notoseris henryi*** (Dunn) C. Shih [= *N. porphyrolepis* C. Shih]: LAC-056: China, Chongqing, Nanchuan, 18 Sep 2010, *Z. H. Wang & L. Chen 255* (KUN), KF485591, KF485718, KF485846, KF486102, KF485974, KF486230; LAC-057: China, Chongqing, Nanchuan, 18 Sep 2010, *Z. H. Wang & L. Chen 249* (KUN), KF485592, KF485719, KF485847, KF486103, KF485975, KF486231; LAC-062: China, Chongqing, Nanchuan, 18 Sep 2010, *Z. H. Wang & L. Chen 259* (KUN), KF485597, KF485724, KF485852, KF486108, KF485980, KF486236; LAC-063: China, Chongqing, Nanchuan, 18 Sep 2010, *Z. H. Wang & L. Chen 260* (KUN), KF485598, KF485725, KF485853, KF486109, KF485981, KF486237. ***N. khasiana*** (C. B. Clarke) N. Kilian [= *N. rhombiformis* C. Shih]: LAC-066: China, Yunnan, Dali, 17 Oct 2010, *Z. J. Yin & al. 3263* (KUN), KF485601, KF485728, KF485856, KF486112, KF485984, KF486240; LAC-067: China, Yunnan, Midu, 17 Apr 2010, *Z. H. Wang 10* (KUN), KF485602, KF485729, KF485857, KF486113, KF485985, KF486241; LAC-068: China, Yunnan, Yunlong, 23 Sep 2011, *Z. H. Wang 473* (KUN), KF485603, KF485730, KF485858, KF486114, KF485986, KF486242. ***N. macilenta*** (Vaniot & H. Lév.) N. Kilian [= *N. psilolepis* C. Shih]: LAC-064: China, Chongqing, Nanchuan, 18 Sep 2010, *Z. H. Wang & L. Chen 235* (KUN), KF485599, KF485726, KF485854, KF486110, KF485982, KF486238; LAC-065: China, Chongqing, Nanchuan, 17 Sep 2010, *Z. H. Wang & L. Chen 243* (KUN), KF485600, KF485727, KF485855, KF486111, KF485983, KF486239. ***N. scandens*** (Hook. f.) N. Kilian [= *Prenanthes scandens* Hook. f.]: LAC-052: China, Yunnan, Gongshan, 11 Nov 2011, *Z. H. Wang, L. Chen & Y. Tang 458* (KUN), KF485587, KF485714, KF485842, KF486098, KF485970, KF486226. ***N. scandens × yakoensis*** [= *Prenanthes scandens* × *yakoensis*]: LAC-053: China, Yunnan, Gongshan, 11 Nov 2011, *Z. H. Wang, L. Chen & Y. Tang 459* (KUN), KF485588, KF485715, KF485843, KF486099, KF485971, KF486227. ***N. triflora*** (Hemsl.) C. Shih: LAC-058: China, Chongqing, Nanchuan, 18 Sep 2010, *Z. H. Wang & L. Chen 262* (KUN), KF485593, KF485720, KF485848, KF486104, KF485976, KF486232; LAC-059: China, Chongqing, Nanchuan, 26 Aug 2009, *H. Peng & al. 574* (KUN), KF485594, KF485721, KF485849, KF486105, KF485977, KF486233; LAC-060: China, Yunnan, Qiaojia,

21 Jul 2009, *Y. S. Chen & Z. H. Wang 9038* (KUN), KF485595, KF485722, KF485850, KF486106, KF485978, KF486234; LAC-061: China, Sichuan, Emei, 4 Aug 2011, *E. D. Liu & al. 3028* (KUN), KF485596, KF485723, KF485851, KF486107, KF485979, KF486235. ***N. yakoensis*** (Jeffrey) N. Kilian [= *Prenanthes yakoensis* Jeffrey]: LAC-054: China, Yunnan, Gongshan, 10 Nov 2011, *Z. H. Wang, L. Chen & Y. Tang 463* (KUN), KF485589, KF485716, KF485844, KF486100, KF485972, KF486228; LAC-055: China, Yunnan, Gongshan, 11 Nov 2011, *Z. H. Wang, L. Chen & Y. Tang 457* (KUN), KF485590, KF485717, KF485845, KF486101, KF485973, KF486229. — ***Paraprenanthes diversifolia*** (Vaniot) N. Kilian [= *P. gracilipes* C. Shih, *P. heptantha* C. Shih & D. J. Liou]: LAC-041: China, Sichuan, Dayi, 24 Jun 2011, *Z. H. Wang & L. Chen 403* (KUN), KF485576, KF485703, KF485831, KF486087, KF485959, KF486215; LAC-040: China, Sichuan, Dayi, 24 Jun 2011, *Z. H. Wang & L. Chen 419* (KUN), KF485575, KF485702, KF485830, KF486086, KF485958, KF486214; LAC-039: China, Sichuan, Dayi, 25 Jul 2009, *Y. S. Chen & Z. H. Wang 9106* (KUN), KF485574, KF485701, KF485829, KF486085, KF485957, KF486213. ***P. melanantha*** (Franch.) Z. H. Wang [= *Notoseris melanantha* (Franch.) C. Shih]: LAC-046: China, Sichuan, Mianning, 2 Aug 2011, *Z. H. Wang & L. Chen 489A* (KUN), KF485581, KF485708, KF485836, KF486092, KF485964, KF486220; LAC-047: China, Sichuan, Mianning, 2 Aug 2011, *Z. H. Wang & L. Chen 489B* (KUN), KF485582, KF485709, KF485837, KF486093, KF485965, KF486221; LAC-048: China, Sichuan, Mianning, 2 Aug 2011, *Z. H. Wang & L. Chen 489C* (KUN), KF485583, KF485710, KF485838, KF486094, KF485966, KF486222. ***P. meridionalis*** (C. Shih) Sennikov [= *P. hastata* C. Shih]: LAC-036: China, Sichuan, Emei, 8 Jul 2010, *Z. H. Wang & L. Chen 57* (KUN), KF485571, KF485698, KF485826, KF486082, KF485954, KF486210. ***P. oligolepis*** (C. C. Chang ex C. Shih) Z. H. Wang [= *Cicerbita oligolepis* C. C. Chang ex C. Shih]: LAC-020: China, Yunnan, Dali, 17 Oct 2010, *Z. J. Yin & al. 3268* (KUN), KF485555, KF485682, KF485810, KF486066, KF485938, KF486194; LAC-021: China, Yunnan, Dali, 17 Oct 2010, *Z. J. Yin & al. 3264* (KUN), KF485556, KF485683, KF485811, KF486067, KF485939, KF486195; LAC-022: China, Yunnan, Dali, 14 Sep 2011, *H. J. Dong & al. 416* (KUN), KF485557, KF485684, KF485812, KF486068, KF485940, KF486196; LAC-023: China, Yunnan, Yunlong, 23 Sep 2011, *Z. H. Wang & L. Chen 472* (KUN), KF485558, KF485685, KF485813, KF486069, KF485941, KF486197. ***P. prenanthoides*** (Hemsl.) C. Shih [= *P. glandulosissima* (C. C. Chang) C. Shih, *P. luchunensis* C. Shih, *P. polypodifolia* (Franch.) C. Shih]: LAC-034: China, Sichuan, Emei, 8 Jul 2010, *Z. H. Wang & L. Chen 67* (KUN), KF485569, KF485696, KF485824, KF486080, KF485952, KF486208; LAC-031: China, Sichuan, Emei, 7 Jul 2010, *Z. H. Wang & L. Chen 43* (KUN), KF485566, KF485693, KF485821, KF486077, KF485949, KF486205; LAC-033: China, Sichuan, Emei, 7 Jul 2010, *Z. H. Wang & L. Chen 44* (KUN), KF485568, KF485695, KF485823, KF486079, KF485951, KF486207; LAC-035: China, Sichuan, Emei, 7 Jul 2010, *Z. H. Wang & L. Chen 52* (KUN), KF485570, KF485697, KF485825, KF486081, KF485953, KF486209. ***P. sororia*** (Miq.) C. Shih [= *P. multiformis* C. Shih, *P. pilipes* (Migo) C. Shih]: LAC-032: China, Sichuan, Emei, 7 Jul 2010, *Z. H. Wang & L. Chen 39* (KUN), KF485567, KF485694, KF485822, KF486078,

KF485950, KF486206; LAC-037: China, Jiangxi, Jinggangshan, 22 May 2011, *H. J. Dong & Z. J. Yin 126* (KUN), KF485572, KF485699, KF485827, KF486083, KF485955, KF486211; (b) LAC-038: China, Jiangxi, Dayu, 24 May 2011, *H. J. Dong & Z. J. Yin 163* (KUN), KF485573, KF485700, KF485828, KF486084, KF485956, KF486212. ***P. triflora*** (Chang & C. Shih) Z. H. Wang & N. Kilian [= *Stenoseris triflora* Chang & C. Shih, *S. leptantha* C. Shih]: LAC-024: China, Yunnan, Jingdong, 27 Nov 2010, *Z. H. Wang & L. Chen 276* (KUN), KF485559, KF485686, KF485814, KF486070, KF485942, KF486198; LAC-025: China, Yunnan, Jingdong, 27 Nov 2010, *Z. H. Wang & L. Chen 272* (KUN), KF485560, KF485687, KF485815, KF486071, KF485943, KF486199; LAC-026: China, Yunnan, Gongshan, 10 Nov 2011, *Z. H. Wang, L. Chen & Y. Tang 462* (KUN), KF485561, KF485688, KF485816, KF486072, KF485944, KF486200; LAC-027: China, Yunnan, Eryuan, 22 Sep 2011, *H. J. Dong & al. 648* (KUN), KF485562, KF485689, KF485817, KF486073, KF485945, KF486201. ***P. umbrosa*** (Dunn) Sennikov [= *Mulgedium umbrosum* (Dunn) C. Shih, *Lactuca parishii* Craib]: LAC-028: Thailand, Chiang Mai, Doi Sutep, 27 Jan 2004, *N. Kilian 10242* (B), KF485563, KF485690, KF485818, KF486074, KF485946, KF486202; LAC-029: China, Yunnan, Longling, 1 May 2011, *Z. H. Wang wzh10* (KUN), KF485564, KF485691, KF485819, KF486075, KF485947, KF486203; LAC-030: China, Yunnan, Jingdong, 1 May 2011, *Z. H. Wang wzh09* (KUN), KF485565, KF485692, KF485820, KF486076, KF485948, KF486204. ***P. wilsonii*** (C. C. Chang) Z. H. Wang [= *Notoseris wilsonii* (C. C. Chang) C. Shih]: LAC-049: China, Sichuan, Dayi, 26 Jun 2011, *Z. H. Wang & L. Chen 344A* (KUN), KF485584, KF485711, KF485839, KF486095, KF485967, KF486223; LAC-050: China, Sichuan, Dayi, 25 Jun 2011, *Z. H. Wang & L. Chen 344B* (KUN), KF485585, KF485712, KF485840, KF486096, KF485968, KF486224; LAC-051: China, Sichuan, Dayi, 25 Jun 2011, *Z. H. Wang & L. Chen 344C* (KUN), KF485586, KF485713, KF485841, KF486097, KF485969, KF486225. ***P. yunnanensis*** (Franch.) C. Shih [= *P. longiloba* Y. Ling & C. Shih, *P. sagittiformis* C. Shih]: LAC-042: China, Yunnan, Kunming, 17 Aug 2010, *Z. H. Wang wzh01* (KUN), KF485577, KF485704, KF485832, KF486088, KF485960, KF486216; LAC-043: China, Yunnan, Kunming, 1 Jul 2010, *C. L. Xiang & al. 429* (KUN), KF485578, KF485705, KF485833, KF486089, KF485961, KF486217; LAC-044: China, Yunnan, Kunming, 1 Jul 2010, *C. L. Xiang & al. 432* (KUN), KF485579, KF485706, KF485834, KF486090, KF485962, KF486218; LAC-045: China, Yunnan, Lanping, 26 Jul 2010, *H. Peng & al. 1006* (KUN), KF485580, KF485707, KF485835, KF486091, KF485963, KF486219
