# Supplementary material for: Molecular Phylogeny of the Lactuca Alliance (Cichorieae Subtribe Lactucinae, Asteraceae) with Focus on Their Chinese Centre of Diversity Detects Potential Events of Reticulation and Chloroplast Capture
Source: PLoS One. 2013 Dec 20;8(12):e82692. doi: 10.1371/journal.pone.0082692 (PMC3871690; doi:10.1371/journal.pone.0082692)
Supplement: Appendix S2 — Positions of mutational hotspots ( = HS) and exons in the individual chloroplast marker sequences excluded from phylogenetic analysis. The position within each marker sequence is calculated without gap; a dash denotes the absence of this sequence portion in the corresponding samples. (PDF) [file pone.0082692.s002.pdf]

Appendix S2. Positions of mutational hotspots (= HS) and exons in the individual chloroplast marker sequences excluded from phylogenetic analysis. The position within each marker sequence is calculated without gap; a dash denotes the absence of this sequence portion in the corresponding samples.

**Sample name in the tree**	***petD* region: exon**	***petD* region: HS1**	***psbA-trnH*: HS1**	***psbA-trnH:* HS2**	***psbA-trnH*: HS3**	***psbA-trnH*: HS4**	***psbA-trnH*: HS5**	***trnL-F*: exon**	***trnL-F*: HS1**	***rpl32-trnL*: HS1**
Launaea sarmentosa_LAC-001	193-200	——	94-101	——	357-365	——	400-417	442-490	——	128-128
Leontodon tuberosua_LAC-002	193-200	——	93-99	——	329-342	——	377-394	431-479	——	132-132
Crepis multicaulis_LAC-003	193-200	——	93-99	——	338-345	——	380-387	440-488	——	132-132
Soroseris erysimoides_LAC-004	188-195	——	94-101	——	331-337	——	372-389	396-444	——	——
Faberiopsis nanchuanensis_LAC-005	200-207	——	94-101	——	331-337	——	372-389	442-490	——	132-132
Faberia sinensis_LAC-006	200-207	——	94-100	——	330-336	——	371-388	442-490	——	132-132
Faberia sinensis_LAC-007	200-207	——	94-101	——	331-337	——	372-389	442-490	——	132-132
Prenanthes faberi_LAC-008	200-207	——	94-101	——	331-337	——	372-389	442-490	——	132-132
Prenanthes faberi_LAC-009	200-207	——	94-101	——	331-336	——	371-388	442-490	——	132-132
Prenanthes faberi_LAC-010	200-207	——	94-101	——	331-336	——	371-388	442-490	——	132-132
Cephalorrhynchus hispidus_LAC-011	193-200	——	94-101	——	327-337	——	372-373	missing	missing	missing
Cicerbita alpina_LAC133	193-200	546-562	94-100	——	326-334	——	369-381	442-490	——	132-132
Prenanthes purpurea_LAC013	193-200	552-568	94-101	——	331-339	——	374-388	442-490	——	132-134
Cicerbita azurea_LAC014	193-200	——	94-101	——	327-339	——	374-385	442-490	——	132-132
Cicerbita azurea_LAC015	193-200	——	94-101	——	327-337	——	372-389	442-490	——	132-132
Stenoseris auriculiformis_LAC-016	193-200	——	missing	missing	missing	missing	missing	442-490	——	132-132
Stenoseris auriculiformis_LAC-017	193-200	——	94-101	——	327-337	——	372-389	442-490	——	132-132
Chaetoseris roborowskii_LAC-018	193-200	——	94-101	——	327-339	——	374-391	442-490	——	132-132
Chaetoseris roborowskii_LAC-019	193-200	——	94-101	——	327-337	——	372-389	442-490	——	132-132
Cicerbita oligolepis_LAC-020	193-200	——	94-101	——	332-344	——	379-396	442-490	——	——
Cicerbita oligolepis_LAC-021	193-200	——	94-101	——	332-344	——	379-396	442-490	——	——
Cicerbita oligolepis_LAC-022	193-200	544-560	94-101	——	332-344	——	379-396	442-490	——	——
Cicerbita oligolepis_LAC-023	193-200	——	94-101	——	332-344	——	379-396	442-490	——	——
Stenoseris leptantha_LAC-024	193-200	——	95-103	——	3-5-316	——	351-358	442-490	——	——
Stenoseris leptantha_LAC-025	193-200	——	95-103	——	305-316	——	351-358	442-490	——	——
Stenoseris triflora_LAC-026	193-200	——	95-103	——	305-317	——	352-359	442-490	——	——
Stenoseris triflora_LAC-027	193-200	——	95-103	——	305-317	——	352-359	442-490	——	——
Lactuca parishii_LAC028	193-200	——	94-101	——	327-334	——	369-386	442-490	——	132-132

Sample name in the tree	*petD* region: exon	*petD* region: HS1	*psbA-trnH*: HS1	*psbA-trnH:* HS2	*psbA-trnH*: HS3	*psbA-trnH*: HS4	*psbA-trnH*: HS5	*trnL-F*: exon	*trnL-F*: HS1	*rpl32-trnL*: HS1
Mulgedium umbrosum_LAC-029	193-200	——	94-101	——	327-334	——	369-396	442-490	——	132-132
Mulgedium umbrosum_LAC-030	193-200	——	94-101	——	327-334	——	369-396	442-490	——	132-132
Paraprenanthes luchunensis_LAC-031	193-200	544-560	93-98	——	324-334	——	369-386	442-490	——	132-132
Paraprenanthes multiformis_LAC-032	193-200	544-560	93-98	——	324-332	——	367-384	442-490	——	132-132
Paraprenanthes polypodifolia_LAC-033	193-200	544-560	93-98	——	324-334	——	369-386	442-490	——	132-132
Paraprenanthes glandulosissima_LAC-034	193-200	544-560	93-98	——	324-334	——	369-386	442-490	——	132-132
Paraprenanthes prenanthoides_LAC-035	193-200	544-560	93-98	——	324-334	——	369-386	442-490	——	132-132
Paraprenanthes hastata_LAC-036	193-200	544-560	93-98	——	324-334	——	369-386	442-490	——	132-132
Paraprenanthes pilipes_LAC-037	193-200	544-560	94-101	——	327-336	——	371-401	442-490	——	132-132
Paraprenanthes pilipes_LAC-038	193-200	544-560	94-101	——	327-335	——	370-421	442-490	——	132-132
Paraprenanthes diversifolia_LAC-039	193-200	544-560	93-98	——	324-334	——	369-386	442-490	——	132-132
Paraprenanthes heptantha_LAC-040	193-200	544-560	93-98	——	324-334	——	369-386	442-490	——	132-132
Paraprenanthes gracilipes_LAC-041	193-200	544-560	missing	missing	missing	missing	missing	442-490	——	132-132
Paraprenanthes longiloba_LAC-042	193-200	544-560	94-101	——	327-336	——	371-388	442-490	——	99-99
Paraprenanthes sagittiformis_LAC-043	193-200	544-560	94-101	——	327-336	——	371-388	442-490	——	99-99
Paraprenanthes sagittiformis_LAC-044	193-200	544-560	94-101	——	327-336	——	371-388	442-490	——	99-99
Paraprenanthes yunnanensis_LAC-045	193-200	544-560	94-101	——	327-337	——	372-389	442-490	——	99-99
Notoseris melanantha_LAC-046	193-200	544-560	94-100	——	353-360	——	395-412	442-490	——	132-132
Notoseris melanantha_LAC-047	193-200	544-560	94-100	——	353-360	——	395-412	442-490	——	132-132
Notoseris melanantha_LAC-048	193-200	544-560	94-100	——	353-360	——	395-412	442-490	——	132-132
Notoseris gracilipes_LAC-049	193-200	544-560	93-100	——	336-345	——	380-397	442-490	——	132-132
Notoseris gracilipes_LAC-050	193-200	544-560	93-100	——	336-345	——	380-397	442-490	——	132-132
Notoseris gracilipes_LAC-051	193-200	544-560	93-100	——	336-345	——	380-397	442-490	——	132-132
Prenanthes scandens_LAC-052	193-200	536-552	94-101	——	327-339	——	374-391	442-490	——	153-153
Prenanthes scandens * yakoensis_LAC-053	193-200	536-552	94-101	——	327-340	——	375-392	442-490	——	153-153
Prenanthes yakoensis_LAC-054	193-200	536-552	94-101	——	327-340	——	375-392	442-490	——	153-153
Prenanthes yakoensis_LAC-055	193-200	536-552	94-101	——	327-342	——	377-394	442-490	——	153-153
Notoseris porphyrolepis_LAC-056	193-200	546-562	94-100	——	326-337	——	372-389	442-490	——	132-132
Notoseris porphyrolepis_LAC-057	193-200	546-562	94-100	——	326-337	——	372-389	442-490	——	132-132
Notoseris triflora_LAC-058	193-200	546-562	94-100	——	326-338	——	373-390	442-490	——	132-132

Sample name in the tree	*petD* region: exon	*petD* region: HS1	*psbA-trnH*: HS1	*psbA-trnH:* HS2	*psbA-trnH*: HS3	*psbA-trnH*: HS4	*psbA-trnH*: HS5	*trnL-F*: exon	*trnL-F*: HS1	*rpl32-trnL*: HS1
Notoseris triflora_LAC-059	193-200	546-562	94-100	——	326-337	——	372-389	442-490	——	132-132
Notoseris triflora_LAC-060	193-200	546-562	94-100	——	326-337	——	372-389	442-490	——	132-132
Notoseris triflora_LAC-061	193-200	546-562	94-100	——	326-337	——	372-389	442-490	——	132-132
Notoseris henryi_LAC-062	193-200	546-562	94-100	——	326-337	——	372-389	442-490	——	132-132
Notoseris henryi_LAC-063	193-200	546-562	94-100	——	326-337	——	372-389	442-490	——	132-132
Notoseris psilolepis_LAC-064	193-200	546-562	94-100	——	326-336	——	371-388	442-490	——	132-132
Notoseris psilolepis_LAC-065	193-200	546-562	94-100	——	326-337	——	372-389	442-490	——	132-132
Notoseris rhombiformis_LAC-066	193-200	544-560	94-100	——	326-337	——	372-389	442-490	——	132-132
Notoseris rhombiformis_LAC-067	193-200	544-560	94-100	——	326-338	——	373-390	442-490	——	132-132
Notoseris rhombiformis_LAC-068	193-200	544-560	94-100	——	326-335	——	370-387	442-490	——	132-132
Parasyncalathium souliei_LAC-069	193-200	——	94-101	——	318-332	——	367-384	442-490	38-40	133-133
Parasyncalathium souliei_LAC-070	193-200	——	94-101	——	327-341	——	376-393	442-490	38-40	133-133
Melanoseris lessertiana_LAC071	193-200	544-560	94-100	——	333-343	——	378-408	442-490	38-40	132-132
Mulgedium bracteatum_LAC-072	193-200	549-565	94-101	——	327-337	——	372-389	442-490	42-42	132-132
Cephalorrhynchus macrorhizus_LAC-073	193-200	544-560	94-101	——	327-335	——	370-400	442-490	38-39	132-132
Cephalorrhynchus macrorhizus_LAC-074	193-200	544-560	94-101	——	327-335	——	370-390	443-491	38-40	121-121
Chaetoseris macrantha_LAC075	193-200	544-560	94-101	——	106-106	——	141-171	442-490	38-40	132-132
Cicerbita sikkimensis_LAC-076	193-200	544-560	94-100	——	326-335	——	370-390	442-490	38-39	132-132
Cicerbita sikkimensis_LAC-077	193-200	544-560	94-100	——	326-335	——	370-390	442-490	38-39	132-132
Mulgedium qinghaicum_LAC-078	193-200	544-560	94-100	——	333-341	——	376-406	442-490	38-40	132-132
Mulgedium qinghaicum_LAC-079	193-200	544-560	94-100	——	333-341	——	376-406	442-490	38-40	132-132
Chaetoseris hastata_LAC-080	193-200	544-560	94-100	——	326-336	——	371-391	442-490	38-40	132-132
Chaetosris yunnanensis_LAC-081	193-200	544-560	94-100	——	326-336	——	371-391	442-490	38-40	132-132
Chaetosris yunnanensis_LAC-082	193-200	544-560	94-100	——	326-336	——	371-391	442-490	38-40	132-132
Chaetoseris cyanea_LAC-083	193-200	544-560	94-100	——	326-336	——	371-391	442-490	38-40	132-132
Chaetosris lutea_LAC-084	193-200	544-560	94-100	——	326-336	——	371-391	442-490	38-40	132-132
Chaetosris lutea_LAC-085	193-200	544-560	94-100	——	326-336	——	371-391	442-490	38-40	132-132
Chaetoseris sichuanensis_LAC-086	193-200	544-560	94-100	——	326-336	——	371-391	442-490	38-40	132-132
Chaetoseris lyriformis_LAC-087	193-200	544-560	94-100	——	326-336	——	371-391	442-490	38-40	132-132
Chaetoseris lyriformis_LAC-088	193-200	544-560	94-100	——	326-336	——	371-391	442-490	38-40	132-132

Sample name in the tree	*petD* region: exon	*petD* region: HS1	*psbA-trnH*: HS1	*psbA-trnH:* HS2	*psbA-trnH*: HS3	*psbA-trnH*: HS4	*psbA-trnH*: HS5	*trnL-F*: exon	*trnL-F*: HS1	*rpl32-trnL*: HS1
Chaetoseris cyanea_LAC-089	193-200	544-560	94-100	——	326-336	——	371-391	442-490	38-40	132-132
Chaetoseris hispida_LAC-090	193-200	544-560	94-100	——	326-337	——	372-392	442-490	38-40	132-132
Chaetoseris lyriformis_LAC-091	193-200	544-560	94-100	——	326-336	——	371-391	442-490	38-40	132-132
Chaetoseris sichuanensis_LAC-092	193-200	544-560	94-100	——	326-336	——	371-391	442-490	38-40	132-132
Chaetoseris *sp2*_LAC093	193-200	544-560	94-100	——	326-336	——	371-391	442-490	38-40	132-132
Chaetoseris *sp4*_LAC094	193-200	544-560	94-100	——	326-336	——	371-391	442-490	38-40	132-132
Chaetoseris *sp5*_LAC095	193-200	544-560	94-100	——	326-336	——	371-391	442-490	38-40	132-132
Chaetoseris *sp6*_LAC096	193-200	544-560	94-100	——	326-336	——	371-391	442-490	38-40	132-132
Chaetoseris *sp7*_LAC097	193-200	544-560	94-100	——	326-336	——	371-391	442-490	38-40	132-132
Chaetoseris likiangensis_LAC-098	193-200	544-560	94-101	——	327-335	——	370-400	442-490	38-40	132-132
Chaetoseris grandiflora_LAC-099	193-200	544-560	94-101	——	327-335	——	370-400	442-490	38-40	132-132
Chaetoseris taliensis_LAC-100	193-200	544-560	94-101	——	327-336	——	371-401	442-490	38-40	132-132
Chaetoseris taliensis_LAC-101	193-200	544-560	94-101	——	327-336	——	371-401	442-490	38-40	132-132
Chaetoseris *sp1*_LAC102	193-200	544-560	94-101	——	327-335	——	370-400	442-490	38-40	132-132
Chaetoseris *sp3*_LAC103	193-200	544-560	94-101	——	327-335	——	370-400	442-490	38-40	132-132
Stenoseris tenuis_LAC-104	193-200	544-560	94-101	——	327-335	——	370-400	442-490	38-40	132-132
Stenoseris tenuis_LAC-105	193-200	544-560	94-101	——	327-335	——	370-400	442-490	38-40	132-132
Stenoseris tenuis_LAC-106	193-200	544-560	94-101	——	327-335	——	370-400	442-490	38-40	——
Stenoseris tenuis_LAC-107	193-200	544-560	94-101	——	327-335	——	370-400	442-490	38-40	132-132
Stenoseris *sp1*_LAC-108	193-200	544-560	94-101	——	327-335	——	370-400	442-490	38-40	132-132
Stenoseris *sp2*_LAC-109	193-200	544-560	94-101	——	327-335	——	370-400	442-490	38-40	132-132
Stenoseris graciliflora_LAC-110	193-200	544-560	94-101	——	327-335	——	370-400	442-490	38-40	132-132
Stenoseris taliensis_LAC-111	193-200	544-560	94-101	——	327-335	——	370-400	442-490	38-40	132-132
Stenoseris graciliflora_LAC-112	193-200	544-560	94-101	——	327-335	——	370-400	442-490	38-40	132-132
Lactuca perennis_LAC334	192-199	——	94-101	——	311-320	——	349-356	442-490	38-39	——
Lactuca undulata_LAC-114	193-200	——	94-102	——	310-321	——	incomplete	442-490	38-39	94-96
Lactuca undulata_LAC-115	193-200	——	94-101	——	311-322	——	357-374	442-490	38-39	94-96
Lactuca dissecta_LAC-116	193-200	——	94-101	301-308	335-347	——	382-389	442-490	38-40	132-132
Lactuca dolichophylla_LAC-117	193-200	——	94-101	301-308	335-345	——	380-392	442-490	38-40	132-132
Steptorhamphus tuberosus_LAC-118	193-200	——	94-102	——	328-338	355-355	374-381	442-490	38-42	132-132

Sample name in the tree	*petD* region: exon	*petD* region: HS1	*psbA-trnH*: HS1	*psbA-trnH:* HS2	*psbA-trnH*: HS3	*psbA-trnH*: HS4	*psbA-trnH*: HS5	*trnL-F*: exon	*trnL-F*: HS1	*rpl32-trnL*: HS1
Lactuca inermis_LAC-119	193-200	544-560	95-102	——	339-349	366-370	389-396	442-490	38-39	123-123
Pterocypsela indica_LAC-120	193-200	——	94-101	301-315	342-353	370-374	392-409	438-486	38-42	132-132
Pterocypsela laciniata_LAC-121	193-200	——	94-101	301-315	342-354	371-375	394-411	438-486	38-42	132-132
Pterocypsela formosana_LAC-122	193-200	——	94-101	——	327-342	359-362	381-398	442-490	38-42	132-132
Pterocypsela sonchus_LAC-123	193-200	——	94-101	301-315	342-357	374-378	397-414	442-490	38-42	132-132
Pterocypsela raddeana_LAC-124	193-200	——	94-101	301-315	342-355	372-375	394-411	442-490	38-42	132-132
Pterocypsela elata_LAC-125	193-200	——	94-101	301-315	342-355	372-376	395-412	442-490	38-42	153-153
Scariola orientalis_LAC126	193-200	544-560	94-101	300-303	338-346	——	380-387	442-490	38-43	132-132
Scariola viminea_LAC-135	193-200	544-560	94-101	296-299	334-344	——	incomplete	442-490	38-42	132-132
Mulgedium sibiricum_LAC-128	193-200	——	94-101	——	327-335	——	370-387	442-490	26-27	132-132
Mulgedium tataricum_LAC-129	188-195	——	94-101	——	327-340	——	375-392	442-490	26-30	132-132
Lactuca sativa_LAC-132	193-200	545-561	94-102	302-305	340-348	——	382-389	442-490	38-41	——
Lactuca serriola_LAC-131	193-200	544-560	94-101	301-304	339-346	——	380-387	442-490	38-41	——

Sample name in the tree	*rpl32-trnL*: HS2	*rpl32-trnL*: HS3	*rpl32-trnL*: HS4	*rpl32-trnL*: HS5	*rpl32-trnL*: HS6	*rpl32-trnL*: HS7	*rpl32-trnL*: HS8	*trnQ-rps16*: HS1	*trnQ-rps16*: HS2	*trnQ-rps16*: HS3
Launaea sarmentosa_LAC-001	160-167	——	377-387	——	697-697	——	777-777	102-103	——	——
Leontodon tuberosua_LAC-002	164-173	190-190	406-416	738-744	——	——	839-839	102-102	——	——
Crepis multicaulis_LAC-003	——	148-148	379-388	——	698-698	——	——	96-96	——	908-917
Soroseris erysimoides_LAC-004	160-167	177-177	393-401	——	——	——	809-809	102-102	——	——
Faberiopsis nanchuanensis_LAC-005	161-167	177-177	393-402	——	——	——	830-831	102-102	——	——
Faberia sinensis_LAC-006	161-167	177-178	394-403	——	——	——	831-832	102-102	——	——
Faberia sinensis_LAC-007	161-167	177-178	394-402	——	——	——	830-831	102-102	——	——
Prenanthes faberi_LAC-008	161-167	177-178	394-404	——	——	——	832-833	102-102	——	——
Prenanthes faberi_LAC-009	161-167	177-178	394-403	——	——	——	831-832	102-102	——	——
Prenanthes faberi_LAC-010	161-167	177-178	394-403	——	——	——	831-832	102-102	——	——
Cephalorrhynchus hispidus_LAC-011	missing	missing	missing	missing	missing	missing	missing	missing	missing	missing
Cicerbita alpina_LAC133	164-174	184	400-420	——	——	——	831-832	102-102	——	——

Sample name in the tree	*rpl32-trnL*: HS2	*rpl32-trnL*: HS3	*rpl32-trnL*: HS4	*rpl32-trnL*: HS5	*rpl32-trnL*: HS6	*rpl32-trnL*: HS7	*rpl32-trnL*: HS8	*trnQ-rps16*: HS1	*trnQ-rps16*: HS2	*trnQ-rps16*: HS3
Prenanthes purpurea_LAC013	166-175	185	401-411	——	——	——	803-804	102-102	——	885-891
Cicerbita azurea_LAC014	164-171	181	397-417	——	——	802-802	829-829	102-102	——	——
Cicerbita azurea_LAC015	164-171	181	397-417	——	——	802-802	829-829	102-102	——	——
Stenoseris auriculiformis_LAC-016	164-174	184	383-403	——	736-736	789-789	816-817	102-102	——	——
Stenoseris auriculiformis_LAC-017	164-174	184	383-403	——	736-736	789-789	816-817	102-102	——	——
Chaetoseris roborowskii_LAC-018	164-174	184	383-403	——	——	788-788	815-816	102-102	——	——
Chaetoseris roborowskii_LAC-019	164-174	184	383-403	——	——	788-788	815-816	102-102	——	——
Cicerbita oligolepis_LAC-020	163-169	179	395-415	——	——	800-801	828-829	102-102	——	——
Cicerbita oligolepis_LAC-021	163-169	179	395-415	——	——	800-801	828-829	102-102	——	——
Cicerbita oligolepis_LAC-022	163-169	179	395-415	——	——	800-801	828-829	102-102	——	——
Cicerbita oligolepis_LAC-023	163-169	179	395-415	——	——	800-801	828-829	102-102	——	——
Stenoseris leptantha_LAC-024	157-163	173	397-417	——	——	802-802	829-830	102-102	——	——
Stenoseris leptantha_LAC-025	157-163	173	397-417	——	——	802-802	829-830	102-102	——	——
Stenoseris triflora_LAC-026	157-163	173	397-417	——	——	802-802	829-830	102-102	——	——
Stenoseris triflora_LAC-027	157-163	173	397-417	——	——	802-802	829-830	102-102	——	——
Lactuca parishii_LAC028	164-175	——	400-420	763-770	——	836-837	864-864	102-102	——	——
Mulgedium umbrosum_LAC-029	164-175	——	400-420	740-747	——	813-813	840-840	102-102	——	——
Mulgedium umbrosum_LAC-030	164-175	——	400-420	740-747	——	813-814	841-841	102-102	——	——
Paraprenanthes luchunensis_LAC-031	164-165	175	391-411	731-738	752-752	793-793	820-820	102-102	——	——
Paraprenanthes multiformis_LAC-032	164-165	175	391-411	731-738	752-752	793-793	820-820	102-102	——	——
Paraprenanthes polypodifolia_LAC-033	164-165	175	391-411	731-738	752-752	796-796	823-823	102-102	——	——
Paraprenanthes glandulosissima_LAC-034	164-165	175	391-411	731-738	752-752	793-793	820-820	102-102	——	——
Paraprenanthes prenanthoides_LAC-035	164-165	175	391-411	731-738	752-752	793-793	820-820	102-102	——	——
Paraprenanthes hastata_LAC-036	164-171	181	397-417	737-744	758-758	799-799	826-826	102-102	——	——
Paraprenanthes pilipes_LAC-037	164-175	185	401-421	741-748	——	814-814	841-841	102-102	——	——
Paraprenanthes pilipes_LAC-038	164-175	185	401-421	741-748	——	814-814	841-841	102-102	——	——
Paraprenanthes diversifolia_LAC-039	164-173	183	399-419	739-746	760-760	801-801	828-828	102-102	——	——
Paraprenanthes heptantha_LAC-040	164-173	183	399-419	739-746	760-760	801-801	828-828	102-102	——	——
Paraprenanthes gracilipes_LAC-041	164-172	182	398-418	738-745	759-759	800-800	827-827	102-102	——	——
Paraprenanthes longiloba_LAC-042	131-138	148	364-384	704-711	——	761-761	788-788	102-102	——	——

Sample name in the tree	*rpl32-trnL*: HS2	*rpl32-trnL*: HS3	*rpl32-trnL*: HS4	*rpl32-trnL*: HS5	*rpl32-trnL*: HS6	*rpl32-trnL*: HS7	*rpl32-trnL*: HS8	*trnQ-rps16*: HS1	*trnQ-rps16*: HS2	*trnQ-rps16*: HS3
Paraprenanthes sagittiformis_LAC-043	131-139	149	365-385	705-712	——	762-762	789-789	102-102	——	——
Paraprenanthes sagittiformis_LAC-044	131-139	149	365-385	705-712	——	762-762	789-789	102-102	——	——
Paraprenanthes yunnanensis_LAC-045	131-139	149	365-385	705-712	——	762-762	789-789	102-102	——	——
Notoseris melanantha_LAC-046	164-172	182	398-418	738-745	——	811-811	838-838	102-102	——	——
Notoseris melanantha_LAC-047	164-172	182	398-418	738-745	——	811-811	838-838	102-102	——	——
Notoseris melanantha_LAC-048	164-172	182	398-418	738-745	——	811-811	838-838	102-102	——	——
Notoseris gracilipes_LAC-049	164-172	182	398-418	738-745	759-759	800-800	827-827	102-102	——	——
Notoseris gracilipes_LAC-050	164-172	182	398-418	738-745	759-759	800-800	827-827	102-102	——	——
Notoseris gracilipes_LAC-051	164-172	182	398-418	738-745	759-759	800-800	827-827	102-102	——	——
Prenanthes scandens_LAC-052	185-195	206	422-432	——	——	817-817	844-845	102-102	——	——
Prenanthes scandens * yakoensis_LAC-053	185-194	205	421-431	——	——	816-816	843-844	102-102	——	——
Prenanthes yakoensis_LAC-054	185-194	205	421-431	——	——	816-816	843-844	102-102	——	——
Prenanthes yakoensis_LAC-055	185-193	204	420-430	——	——	815-815	842-843	102-102	——	——
Notoseris porphyrolepis_LAC-056	164-175	185	401-421	746-753	——	819-819	846-846	102-102	——	——
Notoseris porphyrolepis_LAC-057	164-174	184	400-420	745-752	——	818-818	845-845	102-102	——	——
Notoseris triflora_LAC-058	164-175	185	401-421	746-753	——	819-819	846-846	102-102	——	——
Notoseris triflora_LAC-059	164-174	184	400-420	745-752	——	818-818	845-845	102-102	——	——
Notoseris triflora_LAC-060	164-174	184	400-420	745-752	——	818-819	846-846	102-102	——	——
Notoseris triflora_LAC-061	164-175	185	401-421	746-753	——	819-819	846-846	102-102	——	——
Notoseris henryi_LAC-062	164-175	185	401-421	746-753	——	819-819	846-846	102-102	——	——
Notoseris henryi_LAC-063	164-175	185	401-421	746-753	——	819-819	846-846	102-102	——	——
Notoseris psilolepis_LAC-064	164-175	185	401-421	746-753	——	819-819	846-846	102-102	——	——
Notoseris psilolepis_LAC-065	164-175	185	401-421	——	——	811-811	838-838	102-102	——	——
Notoseris rhombiformis_LAC-066	164-175	185	401-421	741-748	——	814-814	841-841	102-102	——	——
Notoseris rhombiformis_LAC-067	164-175	185	401-421	741-748	——	814-814	841-841	102-102	——	——
Notoseris rhombiformis_LAC-068	164-177	187	403-423	748-755	——	821-821	848-848	102-102	——	——
Parasyncalathium souliei_LAC-069	165-178	192	408-418	——	748-748	801-801	828-828	102-102	——	——
Parasyncalathium souliei_LAC-070	165-177	191	407-417	——	747-747	800-800	827-827	102-102	——	——
Melanoseris lessertiana_LAC071	164-174	184	400-420	——	753-753	806-806	833-833	102-102	——	——
Mulgedium bracteatum_LAC-072	——	148	364-384	——	——	776-776	803-803	102-102	——	——

Sample name in the tree	***rpl32-trnL*: HS2**	***rpl32-trnL*: HS3**	***rpl32-trnL*: HS4**	***rpl32-trnL*: HS5**	***rpl32-trnL*: HS6**	***rpl32-trnL*: HS7**	***rpl32-trnL*: HS8**	***trnQ-rps16*: HS1**	***trnQ-rps16*: HS2**	***trnQ-rps16*: HS3**
Cephalorrhynchus macrorhizus_LAC-073	164-172	182	398-408	——	——	800-800	827-827	102-102	746-751	——
Cephalorrhynchus macrorhizus_LAC-074	153-160	170	386-396	——	——	781-781	808-808	102-102	——	890-900
Chaetoseris macrantha_LAC075	164-174	184	400-420	——	753-753	——	832-832	102-102	——	890-900
Cicerbita sikkimensis_LAC-076	164-174	184	400-420	——	753-753	——	——	102-102	——	890-900
Cicerbita sikkimensis_LAC-077	164-174	184	400-420	——	753-753	——	826-826	102-102	——	890-900
Mulgedium qinghaicum_LAC-078	164-174	184	400-420	——	753-753	806-806	833-833	102-102	——	890-900
Mulgedium qinghaicum_LAC-079	164-173	183	399-419	——	752-752	805-805	832-832	102-102	——	890-900
Chaetoseris hastata_LAC-080	164-172	182	398-418	——	751-751	——	824-824	102-102	——	890-900
Chaetosris yunnanensis_LAC-081	164-172	182	398-418	——	751-751	——	824-824	102-102	——	890-900
Chaetosris yunnanensis_LAC-082	164-174	184	400-420	——	753-753	——	826-826	——	——	888-898
Chaetoseris cyanea_LAC-083	164-172	182	398-418	——	751-751	——	824-824	102-102	——	890-900
Chaetosris lutea_LAC-084	164-172	182	398-418	——	751-751	——	824-824	102-102	——	890-900
Chaetosris lutea_LAC-085	164-172	182	398-418	——	751-751	——	824-824	102-102	——	890-900
Chaetoseris sichuanensis_LAC-086	164-172	182	398-418	——	751-751	——	824-824	102-102	——	890-900
Chaetoseris lyriformis_LAC-087	164-172	182	398-418	——	751-751	——	824-824	102-102	——	890-900
Chaetoseris lyriformis_LAC-088	164-171	181	397-417	——	750-750	——	823-823	102-102	——	890-900
Chaetoseris cyanea_LAC-089	164-172	182	398-418	——	751-751	——	824-824	102-102	——	890-900
Chaetoseris hispida_LAC-090	164-172	182	398-418	——	751-751	——	824-824	102-102	746-751	896-906
Chaetoseris lyriformis_LAC-091	164-172	182	398-418	——	751-751	——	824-824	102-102	——	890-900
Chaetoseris sichuanensis_LAC-092	164-172	182	398-418	——	751-751	——	824-824	102-102	——	890-900
Chaetoseris *sp2*_LAC093	164-172	182	398-418	——	751-751	——	824-824	102-102	——	890-900
Chaetoseris *sp4*_LAC094	164-172	182	398-418	——	751-751	——	824-824	102-102	——	890-900
Chaetoseris *sp5*_LAC095	164-172	182	398-418	——	751-751	——	824-824	102-102	——	890-900
Chaetoseris *sp6*_LAC096	164-172	182	398-418	——	751-751	——	824-824	102-102	——	890-900
Chaetoseris *sp7*_LAC097	164-172	182	398-418	——	751-751	——	824-824	102-102	——	890-900
Chaetoseris likiangensis_LAC-098	164-173	183	398-419	——	758-758	811-811	——	102-102	——	890-900
Chaetoseris grandiflora_LAC-099	164-173	183	398-419	——	758-758	811-811	——	102-102	——	890-900
Chaetoseris taliensis_LAC-100	164-173	183	398-419	——	758-758	811-811	——	102-102	——	895-905
Chaetoseris taliensis_LAC-101	164-173	183	398-419	——	758-758	811-811	——	102-102	——	890-900
Chaetoseris *sp1*_LAC102	164-173	183	398-419	——	758-758	811-811	——	102-102	——	890-900

Sample name in the tree	*rpl32-trnL*: HS2	*rpl32-trnL*: HS3	*rpl32-trnL*: HS4	*rpl32-trnL*: HS5	*rpl32-trnL*: HS6	*rpl32-trnL*: HS7	*rpl32-trnL*: HS8	*trnQ-rps16*: HS1	*trnQ-rps16*: HS2	*trnQ-rps16*: HS3
*Chaetoseris sp3*_LAC103	164-173	183	398-419	——	758-758	811-811	——	102-102	——	——
Stenoseris tenuis_LAC-104	164-173	183	398-419	——	758-758	811-811	——	102-102	——	890-900
Stenoseris tenuis_LAC-105	164-173	183	398-419	——	758-758	811-811	——	102-102	——	890-900
Stenoseris tenuis_LAC-106	163-171	181	397-417	——	756-756	809-809	——	102-102	——	890-900
Stenoseris tenuis_LAC-107	164-173	183	398-419	——	758-758	811-811	——	102-102	——	890-900
Stenoseris *sp1*_LAC-108	164-173	183	398-419	——	758-758	811-811	——	102-102	——	890-900
Stenoseris *sp2*_LAC-109	164-173	183	398-419	——	758-758	811-811	——	102-102	——	890-900
Stenoseris graciliflora_LAC-110	164-173	183	398-419	——	758-758	811-811	——	102-102	——	890-900
Stenoseris taliensis_LAC-111	164-173	183	398-419	——	758-758	811-811	——	102-102	——	890-900
Stenoseris graciliflora_LAC-112	164-173	183	398-419	——	758-758	811-811	——	102-102	——	890-900
Lactuca perennis_LAC334	94-104	114	323-333	——	——	746-747	774-774	102-102	——	——
Lactuca undulata_LAC-114	128-136	146	355-375	——	——	760-760	787-787	102-102	——	——
Lactuca undulata_LAC-115	128-136	146	355-375	——	——	760-760	787-787	102-102	——	——
Lactuca dissecta_LAC-116	164-178	188	403-423	——	——	822-822	849-849	102-102	——	——
Lactuca dolichophylla_LAC-117	164-174	184	398-418	——	——	817-817	844-844	102-102	——	——
Steptorhamphus tuberosus_LAC-118	164-174	184	400-420	——	——	819-819	846-846	102-102	——	——
Lactuca inermis_LAC-119	155-165	175	402-412	——	——	766-766	800-800	102-102	——	——
Pterocypsela indica_LAC-120	164-173	183	399-419	——	——	805-805	832-832	102-102	746-751	——
Pterocypsela laciniata_LAC-121	164-173	183	399-419	——	——	805-805	832-832	102-102	746-751	——
Pterocypsela formosana_LAC-122	164-173	183	399-419	——	——	805-805	832-832	102-102	746-751	——
Pterocypsela sonchus_LAC-123	164-173	183	399-419	——	——	805-805	832-832	102-102	746-751	——
Pterocypsela raddeana_LAC-124	164-173	183	399-419	——	——	805-805	832-832	102-102	——	——
Pterocypsela elata_LAC-125	185-193	203	419-439	——	——	825-825	852-852	102-102	746-751	——
Scariola orientalis_LAC126	164-175	185	401-411	——	——	799-799	826-826	104-105	——	913-923
Scariola viminea_LAC-135	164-174	184	400-410	——	——	799-799	826-826	104-106	——	898-908
Mulgedium sibiricum_LAC-128	164-173	183	400-420	——	——	805-805	832-832	102-102	——	——
Mulgedium tataricum_LAC-129	160-172	182	400-421	——	——	806-806	833-833	102-102	——	——
Lactuca sativa_LAC-132	——	——	319-339	——	——	728-728	755-755	102-104	——	——
Lactuca serriola_LAC-131	——	——	319-339	——	——	745-745	772-772	102-104	——	——
