# Supplementary material for: Molecular Phylogeny of the Lactuca Alliance (Cichorieae Subtribe Lactucinae, Asteraceae) with Focus on Their Chinese Centre of Diversity Detects Potential Events of Reticulation and Chloroplast Capture
Source: PLoS One. 2013 Dec 20;8(12):e82692. doi: 10.1371/journal.pone.0082692 (PMC3871690; doi:10.1371/journal.pone.0082692)
Supplement: Appendix S3 — Indels coded in the phylogenetic analysis. For each marker, position, length [nt] and description of the coded indels are given according to the sequences alignment matrix. (PDF) [file pone.0082692.s003.pdf]

**Table S2. Indels coded in the phylogenetic analysis.** For each marker, position, length [nt] and description of the coded indels are given according to the sequences alignment matrix.

**ITS region**

| No. | Position | Length (nt) | Description |
|---|---|---|---|
| 1 | 4-4 | 1 | Gap in *Faberia sinensis*, *Prenanthes yakoensis* |
| 2 | 16-16 | 1 | Insertion of "A" in *Scariola orientalis*, gap in all other taxa |
| 3 | 40-40 | 1 | Insertion of "C" in *Prenanthes yakoensis*, gap in all other taxa |
| 4 | 40-41 | 2 | Gap in *Launaea sarmentosa* |
| 5 | 43-43 | 1 | Gap in *Prenanthes yakoensis* |
| 6 | 49-49 | 1 | Gap in *Crepis multicaulis* |
| 7 | 59-59 | 1 | Insertion of "T" in *Cicerbita oligolepis* and *Stenoseris triflora, Stenoseris leptantha*, gap in all other taxa |
| 8 | 59-60 | 2 | Gap in *Lactuca perennis* |
| 9 | 83-83 | 1 | Gap in *Soroseris erysimoides* and *Crepis multicaulis* |
| 10 | 85-85 | 1 | Gap in *Soroseris erysimoides* and *Prenanthes purpurea* |
| 11 | 87-87 | 1 | Gap in *Prenanthes purpurea* |
| 12 | 89-89 | 1 | Gap in *Prenanthes purpurea*, *Cicerbita oligolepis* |
| 13 | 91-91 | 1 | Gap in *Lactuca inermis*, all *Pterocypsela* except *P. raddeana*, Scariola *orientalis*, *S. viminea*, *Lagedium sibiricum*, *Mulgedium tataricum*, *L. sativa*, *L. serriola* |
| 14 | 94-94 | 1 | Insertion of "T(C)" in all *Pterocypsela*, Scariola *orientalis*, *S. viminea*, *Lagedium sibiricum*, *Mulgedium tataricum*, *L. sativa*, *L. serriola*, gap in all other taxa |
| 15 | 102-103 | 2 | Gap in *Scariola orientalis* and *S. viminea* |
| 16 | 103-103 | 1 | Insertion of "T" in *Parasyncalathium souliei*, gap in all other taxa |
| 17 | 112-112 | 1 | Gap in *Melanoseris lessertiana* |
| 18 | 118-119 | 2 | Gap in *Launaea sarmentosa*, *Crepis multicaulis*, *Soroseris erysimoides* |
| 19 | 119-119 | 1 | Insertion of "T" in *Faberia sinensis*, gap in all other taxa |
| 20 | 119-120 | 2 | Gap in *Lactuca sativa* and *L. serriola* |
| 21 | 122-122 | 1 | Gap in *Prenanthes purpurea* |
| 22 | 124-124 | 1 | Gap in *Leontodon tuberosus* |
| 23 | 128-129 | 2 | Gap in *Parasyncalathium souliei*, *Lactuc perennis*, *L. undulata*, *L. dissecta*, *L. dolichophylla*, *L. inermis*, *Steptorhamphus tuberosus* |
| 24 | 129-129 | 1 | Insertion of "G" in *Cicerbita azurea* and *Pterocypsela indica*, gap in all other taxa |
| 25 | 129-130 | 2 | Gap in *Launaea sarmentosa* and *Leontodon tuberosus* |
| 26 | 133-134 | 2 | Insertion of "AG" in *Crepis multicaulis*, gap in all other taxa |
| 27 | 141-142 | 2 | Gap in all *Lactuca* clade |
| 28 | 151-151 | 1 | Insertion of "G" in *Crepis multicaulis*, gap in all other taxa |
| 29 | 154-156 | 3 | Gap in *Faberiopsis nanchuanensis* |
| 30 | 165-165 | 1 | Insertion of "A" in *Leontodon tuberosus*,Scariola *orientalis*, *S. viminea*, *Lagedium sibiricum*, *Mulgeium tataricum*, *L. sativa*, *L. serriola*, gap in all other taxa |

**ITS region**

| No. | Position | Length (nt) | Description |
|---|---|---|---|
| 31 | 198-198 | 1 | Gap in *Prenanthes faberi*, *Cephalorrhynchus hispidus*, *Melanoseris lessertiana*, all *Lactuca* clade except *L. perennis* and *L. undulata* |
| 32 | 201-201 | 1 | Insertion of "A" in *Prenanthes purpurea*, gap in all other taxa |
| 33 | 227-236 | 9 | Gap in one of *Faberia sinensis* |
| 34 | 232-232 | 1 | Insertion of "T" in *Launaea sarmentosa* |
| 35 | 246-246 | 1 | Gap in *Lactuca parshii* and *Mulgedium umbrosum* |
| 36 | 249-249 | 1 | Gap in *Lactuca perennis* |
| 37 | 257-257 | 1 | Insertion of "A" in *Cephalorrhynchus hispidus*, gap in all other taxa |
| 38 | 443-443 | 1 | Insertion of "C" in *Lagedium sibiricum* and *Mulgedium tataricum*, gap in all other taxa |
| 39 | 449-449 | 1 | Gap in *Lagedium sibiricum* and *Mulgedium tataricum* |
| 40 | 449-450 | 2 | Gap in *Parasyncalathium souliei* |
| 41 | 459-460 | 2 | Insertion of "AT" in *Leontodon tuberosus* and "CT" in *Crepis multicaulis*, gap in all other taxa |
| 42 | 460-460 | 1 | Gap in *Launaea sarmentosa* |
| 43 | 463-463 | 1 | Insertion of "T" in *Leontodon tuberosus* and *"C" in Lactuca inermis*, gap in all other taxa |
| 44 | 463-465 | 3 | Gap in *Crepis multicaulis* |
| 45 | 465-465 | 1 | Gap in *Soroseris erysimoides* |
| 46 | 468-468 | 1 | Gap in *Notoseris triflora* |
| 47 | 472-476 | 5 | Gap in *Lactuca inermis* |
| 48 | 477-477 | 1 | Gap in *Launaea sarmentosa* and *Prenanthes purpurea* |
| 49 | 478-478 | 1 | Insertion of "T(C, A)" in *Launaea sarmentosa*, *Leontodon tuberosus*, *Crepis multicaulis*, *Prenanthes purpurea*, *Soroseris erysimoides*, all *Faberia*, gap in all other taxa |
| 50 | 483-485 | 3 | Gap in all *Faberia* |
| 51 | 484-484 | 1 | Insertion of "T" in *Soroseris erysimoides* and "C" in *Crepis multicaulis*, gap in all other taxa |
| 52 | 485-485 | 1 | Insertion of "A(T)" in *Launaea sarmentosa*, *Leontodon tuberosus*, *Crepis multicaulis*, *Soroseris erysimoides* and *Prenanthes purpurea*, gap in all other taxa |
| 53 | 487-487 | 1 | Gap in *Cicerbita alpina* |
| 54 | 515-516 | 2 | Gap in *Notoseris rhombiformis* |
| 55 | 516-517 | 2 | Gap in *Leontodon tuberosus*, *Cephalorrhynchus hispidus* and *Cicerbita alpina* |
| 56 | 540-540 | 1 | Gap in *Launaea sarmentosa*, *Leontodon tuberosus*, *Crepis multicaulis*, *Soroseris erysimoides*, all *Faberia*, *Prenanthes purpurea*, *Cephalorrhynchus hispidus*, *Cicerbita alpina* and one of *Stenoseris auriculiformis* |
| 57 | 544-544 | 1 | Insertion of "C" in *Launaea sarmentosa*, *Leontodon tuberosus*, *Crepis multicaulis*, *Soroseris erysimoides*, all *Faberia*, *Prenanthes purpurea*, *Cephalorrhynchus hispidus* and *Cicerbita* clade, gap in all other taxa |
| 58 | 590-590 | 1 | Gap in *Leontodon tuberosus* |
| 59 | 590-591 | 2 | Gap in *Cicerbita oligolepis* |

| ITS region | | | |
|---|---|---|---|
| **No.** | **Position** | **Length (nt)** | **Description** |
| 60 | 598-604 | 7 | Gap in *Lactuca inermis* |
| 61 | 619-619 | 1 | Insertion of "A" in *Faberiopsis nanchuanensis*, gap in all other taxa |
| 62 | 619-620 | 2 | Gap in *Launaea sarmentosa* and *Cephalorrhynchus hispidus* |
| 63 | 633-633 | 1 | Gap in *Launaea sarmentosa* |
| 64 | 637-637 | 1 | Insertion of "C(T)" in *Notoseris wilsonii* and *N.. melanantha*, gap in all other taxa |
| 65 | 640-640 | 1 | Insertion of "A" in one of *Notoseris melanantha*, gap in all other taxa |
| 66 | 650-651 | 2 | Gap in *Cicerbita sikkimensis* |

***petD* region**

| No. | Position | Length (nt) | Description |
|---|---|---|---|
| 1 | 6-11 | 6 | Inserton of "TATAGA" in *Soroseris erysimoides* and all *Faberia* (replicate), gap in all other taxa |
| 2 | 27-31 | 5 | Gap in *Mulgedium tataricum* |
| 3 | 152-162 | 11 | Gap in *Soroseris erysimoides* |
| 4 | 162-162 | 1 | Gap in all *Faberia*, insertion of "A" in all other taxa |
| 5 | 166-167 | 2 | Insertion of "TA" (replicate) in all *Faberia*, gap in all other taxa |
| 6 | 182-182 | 1 | Gap in *Lactuca perennis*, insertion of "T" in all other taxa |
| 7 | 256-256 | 1 | Insertion of "G" in *Launaea sarmentosa*, *Leontodon tuberosus*, *Crepis multicaulis*, *Soroseris erysimoides*, all *Faberia* and *Prenanthes purpurea*, gap in all other taxa |
| 8 | 349-353 | 5 | Gap in *Cephalorrhynchus hispidus* and *Cicerbita alpina*, insertion of "AGATA" in all other taxa |
| 9 | 418-418 | 1 | Insertion of "T" in *Lactuca sativa*, gap in all other taxa |
| 10 | 512-512 | 1 | Insertion of "C" in *Parasyncalathium souliei*, gap in all other taxa |
| 11 | 550-554 | 5 | Insertion of "TTTAT" (replicate) in *Mulgedium bracteatum*, gap in all other taxa |
| 12 | 561-575 | 15 | Gap in *Prenanthes yakoensis* and *P. scandens* |
| 13 | 565-571 | 7 | Insertion of "TAAAAAT" in *Launaea sarmentosa*, *Leontodon tuberosus*, *Crepis multicaulis*, *Soroseris erysimoides*, all *Faberia*, *Prenanthes purpurea*, *Cephalorrynchus tuberosus*, *Cicerbita alpina*, gap in all other taxa |
| 14 | 624-625 | 2 | Insertion of "TC" (replicate) in *Notoseris henryi*, *N. triflora*, *N. psilolepis*, gap in all other taxa |
| 15 | 765-770 | 6 | Gap in all *Faberia* |
| 16 | 776-779 | 4 | Insetion of "TATA" (replicate) in one small clade of *Melanoseris*, i.e. *Chaetoseris likiangensis*, *Ch. grandiflora*, *Stenoseris tenuis*, *S. graciliflora*, including hybrids between them |
| 17 | 801-801 | 1 | Insertion of "A" in *Mulgedium bracteatum*, gap in all other taxa |
| 18 | 819-819 | 1 | Insertion of "T" in *Launaea sarmentosa*, gap in all other taxa |
| 19 | 819-832 | 14 | Insertion of "TTTATATGGATTCA" (replicate) in *Launaea sarmentosa* |
| 20 | 915-915 | 1 | Gap in *Lactuca perennis* and Scariola *viminea*, insertion of "T" in all other taxa |

***psbA-trnH***

| No. | Position | Length (nt) | Description |
|---|---|---|---|
| 1 | 4-4 | 1 | Insertion of "C" in *Lactuca inermis*, gap in all other taxa |
| 2 | 25-25 | 1 | Insertion of "A" in *Stenoseris triflora* and *S. leptantha*, gap in all other taxa |
| 3 | 48-48 | 1 | Gap in *Leontodon tuberosus*, *Crepis multicaulis*, insertion of "A" in all other taxa |
| 4 | 83-83 | 1 | Gap in *Paraprenanthes diversifolia*, *P. prenanthoies*, *P. hastata* |
| 5 | 96-105 | 10 | Insertion of "ATTTTTTTTT" in *Notoseris wilsonii*, gap in all other taxa |
| 6 | 96-117 | 22 | Gap in all taxa except *Notoseris wilsonii*, *Lactuca inermis* |
| 7 | 106-117 | 12 | Insertion of "ATTTTTTTTTTT" in *Lactuca inermis*, gap in all other taxa |
| 8 | 122-127 | 6 | Gap in *Stenseris triflora* and *S. leptantha* |
| 9 | 122-430 | 309 | Gap in *Chaetoseris macrantha* |
| 10 | 155-155 | 1 | Gap in *Launaea sarmentosa* |
| 11 | 161-175 | 15 | Gap in one of *Parasyncalathium souliei* |
| 12 | 169-174 | 6 | Gap in *Cephalorrhynchus hispidus*, *Cicerbita alpina* |
| 13 | 169-175 | 7 | Gap in *Lactuca inermis* |
| 14 | 170-171 | 2 | Insertion of "TT" in *Leontodon tuberosus* |
| 15 | 195-210 | 16 | Insertion of "TTTCTCTTTGTATAAA" in *Launaea sarmentosa*, gap in all other taxa |
| 16 | 213-213 | 1 | Gap in *Scariola orientalis* and *S. viminea* |
| 17 | 252-252 | 1 | Gap in *Leontodon tuberosus* |
| 18 | 263-269 | 7 | Insertion of "GTTTTAT" in *Melanoseris lessertiana* and *Mulgedium qinghaicum*, gap in all other taxa |
| 19 | 263-273 | 11 | Gap in *Scariola viminea* |
| 20 | 314-325 | 12 | Gap in *Lactuca perennis* and *L. undulata* |
| 21 | 332-341 | 10 | Insertion of "ATTTTATTAT" in *Launaea sarmentosa*, gap in all other taxa |
| 22 | 332-350 | 19 | Gap in all taxa except *Launaea sarmentosa*, *Crepis multicaulis* |
| 23 | 342-350 | 9 | Insertion of "TTTACATTT" in *Crepis multicaulis*, gap in all other taxa |
| 24 | 356-386 | 31 | Gap in *Stenseris triflora* and *S. leptantha* |
| 25 | 365-369 | 5 | Insertion of "ATTTT" in *Cicerbita oligolepis*, gap in all other taxa |
| 26 | 374-381 | 8 | Insertion of "GAAAG(T)AAA" in *Scariola orientalis*, *S. viminea*, *Lactuca sativa* and *L. serriola* |
| 27 | 374-385 | 12 | Gap in *Lactuca perennis* and *L. undulata* |
| 28 | 390-416 | 27 | Insertion of "TATTACTTTGATTTCATAAATAAGAAA" in *Notoseris melanantha*, gap in all other taxa |
| 29 | 425-426 | 2 | Gap in one of *Lactuca undulata* |
| 30 | 431-431 | 1 | Gap in *Scariola orientalis and S. viminea* |
| 31 | 431-436 | 6 | Gap in *Lactuca perennis* |
| 32 | 432-432 | 1 | Gap in *Lactuca sativa* and *L. serriola* |
| 33 | 454-454 | 1 | Gap in *Pterocypsela indica* |

***trnL-F***

| No. | Position | Length (nt) | Description |
|---|---|---|---|
| 1 | 110-119 | 10 | Gap in *Soroseris erysimoides* |
| 2 | 182-218 | 37 | Gap in *Soroseris erysimoides* |
| 3 | 191-191 | 1 | Insertion of "T" in one of *Cephalorrhynchus macrorhizus*, gap in all other taxa |
| 4 | 191-196 | 6 | Gap in *Leontodon tuberosus* |
| 5 | 217-222 | 6 | Gap in *Leontodon tuberosus* |
| 6 | 248-251 | 4 | Gap in *Pterocypsela indica* and *P. laciniata* |
| 7 | 263-266 | 4 | Insertion of "CATA" (replicate) in *Crepis multicaulis*, gap in all other taxa |
| 8 | 290-290 | 1 | Gap in *Crepis multicaulis* |
| 9 | 292-295 | 4 | Gap in *Crepis multicaulis* |
| 10 | 308-308 | 1 | Gap in *Crepis multicaulis* |
| 11 | 472-487 | 16 | Gap in *Lagedium sibiricum* and *Mulgedium tataricum* |
| 12 | 484-487 | 4 | Insertion of "TTTA" in *Mulgedium bracteatum*, gap in all other taxa |
| 13 | 484-507 | 24 | Gap in *Launaea sarmentosa* |
| 14 | 484-508 | 25 | Gap in *Crepis multicaulis* |
| 15 | 494-506 | 13 | Gap in *Scariola orientalis* |
| 16 | 500-506 | 7 | Insertion of "CTTTATC" in *Prenanthes scandens* and *P. yakoensis* |
| 17 | 508-508 | 1 | Gap in *Soroseris erysimoides*, insertion of "T" in all other taxa |
| 18 | 570-571 | 2 | Gap in all *Pterocypsela* |
| 19 | 575-577 | 3 | Insertion of "TAA" in *Crepis multicaulis* |
| 20 | 575-588 | 14 | Gap in *Cicerbita azurea* |
| 21 | 575-589 | 15 | Gap in all but *Launaea sarmentosa*, *Leontodon tuberosus*, *Crepis multicaulis*, *Soroseris erysimoides*, all *Faberia*, *Prenanthes purpurea*, *Cicerbita alpina*, *C. azurea* |
| 22 | 596-597 | 2 | Insertion of "TT" in *Mulgedium bracteatum*, gap in all other taxa |
| 23 | 609-612 | 4 | Gap in *Lactuca perennis* |
| 24 | 610-611 | 2 | Insertion of "AT" (SSR) in *Lactuca inermis*, gap in all other taxa |
| 25 | 610-619 | 10 | Gap in *Crepis multicaulis*, *Soroseris erysimoides* and all *Faberia* |
| 26 | 643-647 | 5 | Insertion of "GGAAT" (replicate) in *Lagedium sibiricum* and *Mulgedium tataricum*, gap in all other taxa |
| 27 | 667-674 | 8 | Gap in *Crepis multicaulis* |
| 28 | 743-747 | 5 | Gap in *Scariola orientalis* |
| 29 | 789-794 | 6 | Gap in one of *Notoseris rhombiformis*, insertion of "ATGAAA" in all other taxa |
| 30 | 793-798 | 6 | Gap in *Crepis multicaulis* |
| 31 | 808-811 | 4 | Gap in all *Pterocypsela*, insertion of "ATGA" in all other taxa |
| 32 | 813-817 | 5 | Insertion of "GAATA" (replicate) in *Lactuca perennis* and *L. undulata*, gap in all other taxa |

***trnQ-rps16***

| No. | Position | Length (nt) | Description |
|---|---|---|---|
| 1 | 79-84 | 6 | Gap in *Crepis multicaulis* |
| 2 | 94-95 | 2 | Insertion of "AT" (replicate) in *Scariola orientalis* and *S. viminea*, gap in all other taxa |
| 3 | 115-130 | 16 | Insertion of "TTTTTCTTAGCTACAT" (replicate) in *Scariola orientalis*, gap in all other taxa |
| 4 | 139-152 | 14 | Insertion of "AAACATAGATAATC" in *Soroseris erysimoides*, gap in all other taxa |
| 5 | 161-161 | 1 | Insertion of "T" in *Cicerbita* clade, *Melanoseris* clade and *Lactuca* clade, gap in other clade |
| 6 | 161-162 | 2 | Gap in *Paraprenanthes longiloba* |
| 7 | 196-196 | 1 | Gap in *Crepis multicaulis*, *Lactuca sativa* and *L. serriola*, insertion of "T" in all other taxa |
| 8 | 209-215 | 7 | Gap in *Lagedium sibiricum* |
| 9 | 240-240 | 1 | Gap in *Crepis multicaulis* |
| 10 | 265-269 | 5 | Insertion of "AAAAT" in all *Faberia*, gap in all other taxa |
| 11 | 287-304 | 18 | Gap in *Lactuca perennis* |
| 12 | 317-317 | 1 | Insertion of "A" in *Cicerbita alpina*, gap in all other taxa |
| 13 | 332-339 | 8 | Insertion of "TTGACTCC" in *Crepis multicaulis* |
| 14 | 354-362 | 9 | Gap in *Prenanthes purpurea* |
| 15 | 359-362 | 4 | Insertion of "TCAA" in *Scariola orientalis*, *S. viminea*, *Lactuca sativa* and *L. serriola*, gap in all other taxa |
| 16 | 456-465 | 10 | Insertion of "ATCATTATTT" (replicate) in *Soroseris erysimoides*, gap in all other taxa |
| 17 | 472-476 | 5 | Insertion of "ATTTG" (replicate) in one of *Chaetoseris taliensis*, gap in all other taxa |
| 18 | 495-502 | 8 | Gap in *Launaea sarmentosa* |
| 19 | 495-503 | 9 | Insertion of "TTTTACAGT" (replicate) in *Soroseris erysimoides*, gap in all other taxa |
| 20 | 522-559 | 38 | Gap in all but *Mulgedium umbrosum* and *Lactuca dissecta* |
| 21 | 547-559 | 13 | Gap in *Lactuca dissecta* |
| 22 | 571-578 | 8 | Insertion of "ATAAGATC" in *Lactuca perennis*, gap in all other taxa |
| 23 | 571-586 | 16 | Gap in all but *Lactuca perennis*, *Notoseris henryi*, *N. triflora*, *N. psilolepis* |
| 24 | 579-586 | 8 | Insertion of "ATAAGAAA" in *Notoseris henryi*, *N. triflora*, *N. psilolepis*, gap in all other taxa |
| 25 | 614-617 | 4 | Insertion of "TCGA" in all *Paraprenanthes* but *P. pilipes*, *Notoseris melanantha*, gap in all other taxa |
| 26 | 639-643 | 5 | Insertion of "TTAAA" (replicate) in *Lactuca perennis* and *L. undulata*, gap in all other taxa |
| 27 | 668-673 | 6 | Insertion of "ATAGAT" (replicate) in *Lactuca dissecta* and *L. dolichophylla* |
| 28 | 698-710 | 13 | Insertion of "GTTAAATATTTAA" (replicate) in one of *Notoseris rhombiformis,* gap in all other taxa |
| 29 | 786-786 | 1 | Insertion of "T" in *Launaea sarmentosa* |
| 30 | 805-836 | 32 | Gap in all taxa but *Crepis multicaulis* and *Soroseris erysimoides* and all *Faberia* |
| 31 | 814-832 | 19 | Gap in *Soroseris erysimoides* |
| 32 | 814-836 | 23 | Gap in all taxa but *Crepis multicaulis* and *Soroseris erysimoides* |

***trnQ-rps16***

| No. | Position | Length (nt) | Description |
|---|---|---|---|
| 33 | 839-839 | 1 | Insertion of "T" in *Prenanthes purpurea* |
| 34 | 845-851 | 7 | Insertion of "CCTATAG" (replicate) in one of *Notoseris wilsonii*, gap in all other taxa |
| 35 | 857-857 | 1 | Gap in *Soroseris erysimoides* |
| 36 | 867-867 | 1 | Gap in *Crepis multicaulis* |
| 37 | 887-891 | 5 | Insertion of "TTTTG" (replicate) in *Lactuca parshii* and *Mulgedium umbrosum*, gap in all other taxa |
| 38 | 901-904 | 4 | Gap in *Steptorhamphus tuberosus*, insertion of "ACCT" in all other taxa |
| 39 | 947-951 | 5 | Gap in *Lactuca inermis*, insertion of "TTATC" in all other taxa |
| 40 | 963-965 | 3 | Gap in *Lactuca perennis* and *L. undulata*, insertion of "CAC" in all other taxa |
| 41 | 1014-1030 | 17 | Gap in *Crepis multicaulis* |
| 42 | 1024-1028 | 5 | Insertion of "AACTG" (replicate) in *Lactuca undulata*, gap in all other taxa |
| 43 | 1066-1079 | 14 | Insertion of "AAAAAAGAAAGAAG" (replicate) in *Pterocypsela indica*, gap in all other taxa |
| 44 | 1086-1109 | 24 | Gap in *Leontodon tuberosus* |
| 45 | 1087-1090 | 4 | Gap in *Mulgedium bracteatum* |
| 46 | 1097-1099 | 3 | Gap in *Cicerbita* clade II |
| 47 | 1110-1117 | 8 | Gap in *Prenanths yakoensis* and *P. scandens* |

***rpl32-trnL***

| No. | Position | Length (nt) | Description |
|---|---|---|---|
| 1 | 9-62 | 54 | Gap in *Paraprenanthes yunnanensis*, *P. saggitiformis*, *P. longiloba* |
| 2 | 42-62 | 21 | Insertionof "ATCAATACTTTTTTAATATAA" in *Prenanthes yakoensis*, *P. scandens*, gap in all other taxa |
| 3 | 73-81 | 9 | Gap in *Launaea sarmentosa* |
| 4 | 75-80 | 6 | Gap in *Stenoseris triflora*, *S. leptantha* |
| 5 | 75-85 | 11 | Gap in one of *Cephalorrhynchus macrorhizus* |
| 6 | 75-134 | 60 | Gap in *Lactuca perennis*, *L. undulata* |
| 7 | 78-86 | 9 | Gap in *Lactuca inermis* |
| 8 | 103-123 | 21 | Insertion of "CTATTCTAATCAATATGAACC" (replicate) in *Pterocypsela elata*, gap in all other taxa |
| 9 | 128-128 | 1 | Insertion of "A" in *Parasyncalathium souliei*, gap in all other taxa |
| 10 | 153-237 | 85 | Gap in *Lactuca sativa* and *L. serriola* |
| 11 | 157-162 | 6 | Insertion of "AGGTTT" (replicate) in *Launaea sarmentosa*, gap in all other taxa |
| 12 | 167-167 | 1 | Gap in *Launaea sarmentosa* |
| 13 | 181-222 | 42 | Gap in *Lactuca perennis* |
| 14 | 192-227 | 36 | Gap in *Mulgedium bracteatum* |
| 15 | 208-222 | 15 | Gap in *Mulgedium tataricum* |
| 16 | 209-222 | 14 | Gap in *Soroseris erysimoides* and all *Faberia* |
| 17 | 212-213 | 2 | Insertion of "GC" in *Leontodeon tuberosus*, gap in all other taxa |
| 18 | 212-222 | 11 | Gap in all taxa but *Leontodon tuberosus*, *Parasyncalathium souliei* |
| 19 | 212-227 | 16 | Gap in *Launaea sarmentosa* |
| 20 | 214-217 | 4 | Insertion of "ACAA' in *Parasyncalathium souliei*, gap in all other taxa |
| 21 | 218-222 | 5 | Insertion of "AGAAT" (replicate) in *Leontodon tuberosus*, gap in all other taxa |
| 22 | 259-266 | 8 | Insertion of "TTTTTTTT" in *Crepis multicaulis*, gap in all other taxa |
| 23 | 259-278 | 20 | Gap in all taxa except *Crepis multicaulis*, *Lactuca inermis* |
| 24 | 267-278 | 12 | Insertion of "GTAGTATATTTT" (replicate) in *Lactuca inermis*, gap in all other taxa |
| 25 | 300-307 | 8 | Insertion of "TTTTGTGG" (replicate) in *Stenoseris triflora* and *S. leptantha*, gap in all other taxa |
| 26 | 300-308 | 9 | Gap in *Lactuca inermis* |
| 27 | 314-317 | 4 | Insertion of "GGTG" (replicate) in *Lagedium sibiricum* and *Mulgedium tataricaum*, gap in all other taxa |
| 28 | 331-331 | 1 | Gap in *Lactuca dolichophylla* |
| 29 | 350-351 | 2 | Gap in *Lagedium sibiricum* |
| 30 | 354-360 | 7 | Gap in *Lactuca perennis* and *L. undulata* |
| 31 | 383-383 | 1 | Gap in *Lactuca dissecta* and *L. dolichophylla* |

***rpl32-trnL***

| No. | Position | Length (nt) | Description |
|---|---|---|---|
| 32 | 393-408 | 16 | Gap in *Launaea sarmentosa* |
| 33 | 400-400 | 1 | Gap in *Lagedium sibiricum and Mulgedium tataricaum* |
| 34 | 402-408 | 7 | Insertion of "AGTTTTT" in *Crepis multicaulis*, gap in all other taxa |
| 35 | 420-436 | 17 | Gap in *Stenoseris auriculiformis* and *Chaetoseris roborowskii* |
| 36 | 456-456 | 1 | Gap in *Launaea sarmentosa* |
| 37 | 502-524 | 23 | Insertion of "TTTTTAGTAATTACTATATGAAA" in *Lactuca parshii*, gap in all other taxa |
| 38 | 545-550 | 6 | Insertion of "AATTTT" (replicate) in in one small clade of *Melanoseris*, i.e. *Chaetoseris likiangensis*, *Ch. grandiflora*, *Stenoseris tenuis*, *S. graciliflora*, including hybrids between them, gap in all other taxa |
| 39 | 581-594 | 14 | Insertion of "ATTGTTGC(A)GATATT" in *Lactuca dissecta*, *L. dolichophylla*, *Steptorhamphus tuberosus*, gap in all other taxa |
| 40 | 598-643 | 46 | Gap in *Crepis multicaulis* |
| 41 | 603-630 | 28 | Gap in *Launaea sarmentosa* |
| 42 | 608-630 | 23 | Insertion of "AAAGAACTTCATTGTTGAGATAT" (replicate) in *Lactuca perennis*, gap in all other taxa |
| 43 | 674-710 | 37 | Gap in *Soroseris erysimoides* and *Prenanthes purpurea* |
| 44 | 690-695 | 6 | Gap in *Launaea sarmentosa*, *Leontodon tuberosus*, *Crepis multicaulis* |
| 45 | 693-693 | 1 | Insertion of "A" in all *Faberia*, gap in all other taxa |
| 46 | 696-701 | 6 | Insertion of "T(G)GGTTA" in *Launaea sarmentosa*, *Leontodon tuberosus*, *Crepis multicaulis*, *Cicerbita alpina*, gap in all other taxa |
| 47 | 696-706 | 11 | Gap in all taxa but *Launaea sarmentosa*, *Leontodon tuberosus*, *Crepis multicaulis*, *Cicerbita alpina*, all *Notoseris* taxa but two of *N. rhombiformis* |
| 48 | 702-706 | 5 | Insertion of all *Notoseris* taxa but two of *N. rhombiformis*, gap in all other taxa |
| 49 | 731-731 | 1 | Insertion of "C" in *Crepis multicaulis*, gap in all other taxa |
| 50 | 737-737 | 1 | Gap in *Scariola orientalis*, insertion of "T" in all other taxa |
| 51 | 743-743 | 1 | Insertion of "A" in *Soroseris erysimoides*, gap in all other taxa |
| 52 | 743-759 | 17 | Gap in all taxa but *Soroseris erysimoides* and all *Faberia* |
| 53 | 785-789 | 5 | Insertion of "AAATA" (replicate) in *Soroseris erysimoides*, gap in all other taxa |
| 54 | 802-806 | 5 | Insertion of "ATCAAGT" in *Scariola orientalis* and *S. viminea* |
| 55 | 802-813 | 12 | Gap in all taxa but *Cephalorrhynchus macrorhizus*, *Scariola orientalis*, *S. viminea* |
| 56 | 802-833 | 32 | Gap in *Launaea sarmentosa* |
| 57 | 807-813 | 7 | Insertion of "ATCAAGT" in *Cephalorrhynchus macrorhizus*, gap in all other taxa |
| 58 | 820-826 | 7 | Gap in *Cicerbita alpina* |
| 59 | 821-856 | 36 | Gap in *Lactuca inermis* |
| 60 | 832-832 | 1 | Insertion of "A" in *Leontondon tuberosus*, gap in all other taxa |
| 61 | 832-833 | 2 | Gap in *Crepis multicaulis* |
| 62 | 842-842 | 1 | Insertion of "T" in all *Pterocypsela* and *Cicerbita alpina*, gap in all other taxa |

***rpl32-trnL***

| No. | Position | Length (nt) | Description |
|---|---|---|---|
| 63 | 850-852 | 3 | Gap in *Parasyncalathium souliei* |
| 64 | 861-862 | 2 | Gap in *Lactuca inermis* |
| 65 | 891-897 | 7 | Gap in all taxa but *Prenanthes purpurea* and *Mulgedium bracteatum* |
| 66 | 897-897 | 1 | Gap in *Prenanths purpurea* |
| 67 | 903-907 | 5 | Insertion of "ATAAT" (replicate) in *Lactuca perennis*, gap in all other taxa |
| 68 | 930-951 | 22 | Gap in all taxa but *Lactuca sativa* and *L. serriola* |
| 69 | 935-951 | 17 | Gap in *Lactuca sativa* |
| 70 | 964-967 | 4 | Gap in *Paraprenanthes yunnanensis*, *P. saggitiformis*, *P. longiloba* |
| 71 | 965-965 | 1 | Gap in *Crepis multicaulis* |
| 72 | 966-966 | 1 | Gap in *Scariola orientalis*, *S. viminea*, *Lactuca sativa* and *L. serriola* |
| 73 | 971-973 | 3 | Insertion of "GAC" in *Paraprenanthes polypodifolia*, gap in all other taxa |
| 74 | 971-978 | 8 | Gap in all taxa except *Lactuca inermis* and *Paraprenanthes polypodifolia* |
| 75 | 974-978 | 5 | Insertion of "AGGAC" in *Lactuca inermis*, gap in all other taxa |
| 76 | 983-983 | 1 | Insertion of "C" in *Crepis multicaulis*, gap in all other taxa |
| 77 | 987-989 | 3 | Gap in all taxa except *Launaea sarmentosa*, *Leontodon tuberosus*, *Crepis multicaulis* |
| 78 | 988-989 | 2 | Gap in *Launaea sarmentosa* |
| 79 | 989-989 | 1 | Insertion of "A" in *Leontondon tuberosus*, gap in all other taxa |
| 80 | 1009-1020 | 12 | Gap in all *Paraprenanthes* but *P. pilipes* and *Notoseris melanantha* |
| 81 | 1013-1018 | 6 | Gap in *Cicerbita sikkimensis* and *Chaetoseris cyanea* complex |
| 82 | 1048-1054 | 7 | Insertion of "AATTTGA" (replicate) in *Lactuca inermis*, gap in all other taxa |
| 83 | 1086-1090 | 5 | Gap in *Lactuca undulata* |
| 84 | 1101-1101 | 1 | Insertion of "C" in *Stenoseris auriculiformis*, gap in all other taxa |
